# Ketamine Restores Thalamic-Prefrontal Cortex Functional Connectivity in a Mouse Model of Neurodevelopmental Disorder-Associated 2p16.3 Deletion

**DOI:** 10.1093/cercor/bhz244

**Published:** 2019-12-08

**Authors:** Rebecca B Hughes, Jayde Whittingham-Dowd, Rachel E Simmons, Steven J Clapcote, Susan J Broughton, Neil Dawson

**Affiliations:** 1 Division of Biomedical and Life Sciences, Faculty of Health and Medicine, Lancaster University, Lancaster LA1 4YQ, UK; 2 School of Biomedical Sciences, University of Leeds, Leeds LS2 9JT, UK

**Keywords:** autism, functional brain imaging, graph theory, NMDA receptor, schizophrenia

## Abstract

2p16.3 deletions, involving heterozygous *NEUREXIN1* (*NRXN1*) deletion, dramatically increase the risk of developing neurodevelopmental disorders, including autism and schizophrenia. We have little understanding of how *NRXN1* heterozygosity increases the risk of developing these disorders, particularly in terms of the impact on brain and neurotransmitter system function and brain network connectivity. Thus, here we characterize cerebral metabolism and functional brain network connectivity in *Nrxn1α* heterozygous mice (*Nrxn1α*^*+/−*^ mice), and assess the impact of ketamine and *dextro*-amphetamine on cerebral metabolism in these animals. We show that heterozygous *Nrxn1α* deletion alters cerebral metabolism in neural systems implicated in autism and schizophrenia including the thalamus, mesolimbic system, and select cortical regions. *Nrxn1α* heterozygosity also reduces the efficiency of functional brain networks, through lost thalamic “rich club” and prefrontal cortex (PFC) hub connectivity and through reduced thalamic-PFC and thalamic “rich club” regional interconnectivity. Subanesthetic ketamine administration normalizes the thalamic hypermetabolism and partially normalizes thalamic disconnectivity present in *Nrxn1α*^*+/−*^ mice, while cerebral metabolic responses to *dextro*-amphetamine are unaltered. The data provide new insight into the systems-level impact of heterozygous *Nrxn1α* deletion and how this increases the risk of developing neurodevelopmental disorders. The data also suggest that the thalamic dysfunction induced by heterozygous *Nrxn1α* deletion may be NMDA receptor-dependent.

## Introduction

Copy number variants (CNVs) are strongly implicated in the genetic etiology of schizophrenia (ScZ) and autism (ASD). Population-based studies show that deletions at 2p16.3, involving heterozygous deletion of the *NEUREXIN1* (*NRXN1*) gene, are associated with developmental delay, learning difficulties, and a dramatically increased risk of developing ASD (odds ratio = 14.9) (Matsunami et al. 2013; Yuen et al. 2017) and ScZ (odds ratio = 14.4) (Marshall et al. 2017). Individuals with *NRXN1* deletions also show symptoms of attention deficit hyperactivity disorder (ADHD) (Ching et al. 2010; Schaaf et al. 2012). Heterozygous *NRXN1α* deletions were first reported in small case studies of individuals with ASD (Friedman et al. 2006) and ScZ (Kirov et al. 2009) and have subsequently been identified in other cases (see Reichelt et al. 2012 for review). Most *NRXN1* deletions observed in ScZ and ASD localize to the promoter and initial exons of *NRXN1α* and leave the *NRXN1β* coding sequences intact (Reichelt et al. 2012); thus, they are predicted to impact on *NRXN1α* but not *NRXN1β* transcripts.

Neurexins function as presynaptic cell adhesion molecules forming trans-synaptic interaction complexes with a range of postsynaptic binding partners, including neuroligins, to regulate synaptic differentiation, maturation, and function (Reichelt et al. 2012; Sudhof 2008, 2017). Neurexins undergo extensive alternative splicing, which regulates their binding interactions, with isoforms being differentially expressed throughout the brain and across development (Ullrich et al. 1995; Schreiner et al. 2014; Treutlein et al. 2014).

Multiple rodent studies have been dedicated to elucidating the behavioral consequences of neurexin deficiency to establish whether these result in phenotypes relevant to ASD and ScZ. For example, *Nrxn1α* homozygous knockout (KO) mice display decreased social interaction and increased anxiety-like behavior (Grayton et al. 2013), which may relate to core symptoms of these disorders, along with a deficit in prepulse inhibition (Etherton et al. 2009) that mirrors the sensorimotor gating deficits seen in ASD and ScZ (Braff et al. 1999; Cheng et al. 2018). Studies undertaken in *Nrxn1α* KO rats also support a role for *Nrxn1α* in cognition and learning, but found no evidence for altered sensorimotor gating (Esclassan et al. 2015). As *NRXN1* deletions in ASD and ScZ are commonly heterozygous, other studies have focused on *Nrxn1α* heterozygous (*Nrxn1α^+/−^*) mice, reporting sex-dependent alterations in novelty responsiveness and habituation (Laarakker et al. 2012) and memory deficits (Dachtler et al. 2015). *Nrxn1α^+/−^* mice also show deficits in social memory, while effects on sociability and anxiety-like behavior appear to be limited (Grayton et al. 2013; Dachtler et al. 2015).

Evidence implicates glutamate neurotransmitter system and NMDA receptor (NMDA-R) dysfunction in ASD (Lee et al. 2015; Horder et al. 2018) and ScZ (Howes et al. 2015; Dauvermann et al. 2017), and the NMDA-R is proposed as a potential therapeutic target in both disorders. Drugs modulating NMDA-R function are hypothesized as potential treatments for ScZ (Dauvermann et al. 2017), while evidence suggests that either facilitating or reducing NMDA-R function may have therapeutic potential in ASD (Lee et al. 2015). For example, the NMDA-R antagonist, Memantine, shows clinical promise for some ASD symptoms (Chez et al. 2007; Ghaleiha et al. 2013), although positive effects are not always found (Aman et al. 2017). The reason for the disparity between studies is unknown but may relate to disease heterogeneity. Thus, predictive biomarkers of NMDA-R drug efficacy, to allow patient stratification and a personalized medicine approach, in ASD are urgently needed. There is also strong evidence to support monoamine (dopamine, serotonin, and noradrenaline) system dysfunction in ScZ and ASD (Selvaraj et al. 2014; Howes et al. 2015; Muller et al. 2016). For example, responses to the monoaminergic releasing stimulant *dextro*-amphetamine (*d*-amphetamine) are altered in ScZ (Breier et al. 1997; Swerdlow et al. 2018), while the drug is used therapeutically in ASD (Nickels et al. 2008; Cortese et al. 2012).

Our understanding of the impact of *Nrxn1α* heterozygosity on glutamate and monoamine neurotransmitter system function is incomplete. However, *Nrxn1α* has been shown to regulate glutamatergic synapse formation (Siddiqui et al. 2010), and complete ablation of *Nrxn1α* impairs excitatory, but not inhibitory, neurotransmission in the hippocampus (Etherton et al. 2009). Moreover, Nrxn1α’s interactions with leucine-rich repeat transmembrane proteins (LRRTMs) may act to regulate excitatory synapse formation, postsynaptic glutamate receptor levels (including the NMDA-R and AMPA-R), glutamatergic neurotransmission, and NMDA-R-dependent LTP (de Wit et al. 2009; Soler-Llavina et al. 2011; Siddiqui 2013, Um 2016). Nrxn1α may also influence glutamatergic neurotransmission through its interactions with cerebellins (C1qls), which bind with high affinity to postsynaptic kainite (GluK2, GluK4) and AMPA-R (GluA1) glutamate receptors (Cheng et al. 2016; Matsuda et al. 2016). Similarly, through its interactions with neuroligin 1 (Nrlgn1), Nrxn1α can also modulate glutamatergic synaptic function through the modification of NMDA-R and AMPA-R function (Chubykin et al. 2007; Barrow et al. 2009; Espinosa et al. 2015; Chanda et al. 2017). More recently, alternative splicing of presynaptic *Nrxn1*, at splice site 4 (SS4), has also been shown to regulate postsynaptic NMDA-R function (Dai et al. 2019), further linking *Nrxn1* to the regulation of the NMDA-R. In addition, *Nrxn1* regulates presynaptic neurotransmitter release, in part through the regulation of Ca^2+^ channels, presynaptic Ca^2+^ transients, and subsequent synaptic vesicle exocytosis (Missler et al. 2003; Pak et al. 2015; Chen et al. 2017; Tong et al. 2017; Brockhaus et al. 2018). Despite these observations, the impact of *Nrxn1α* heterozygosity on in vivo glutamate/NMDA-R and monoaminergic system function has not been characterized.

Between the relatively well-characterized molecular and behavioral effects of *Nrxn1*α, we have little understanding of the systems-level alterations that result from *Nrxn1a* heterozygosity. This includes the impact on brain function, brain network connectivity, and neurotransmitter system function. Here, we characterize the impact of *Nrxn1α* heterozygosity on cerebral metabolism and functional brain network connectivity. In addition, we characterize the cerebral metabolic response to ketamine and *d*-amphetamine to elucidate the impact of *Nrxn1a* heterozygosity on in vivo NMDA-R and monoaminergic system function.

## Materials and Methods

### Animals

Full animal details can be found in the study of Dachtler et al. (2014). In brief, male B6;129-*Nrxn3*^tm1Sud^/*Nrxn1*^tm1Sud^/*Nrxn2*^tm1Sud^/J mice (The Jackson Laboratory, Stock #006377) were purchased as heterozygous KO at *Nrxn1α*, homozygous KO at *Nrxn2,* and wild-type (WT) at *Nrxn3* and outbred to the C57BL/6NCrl strain (Charles River, Margate, UK) to obtain mice individually heterozygous for *Nrxn1α*. Experimental animals (*Nrxn1a^+/−^* and WT littermates, aged 10–22 weeks) were generated through cousin mating. Animals were group housed (4–6/cage) under standard conditions (individually ventilated cages, 21°C, 45–65% humidity, 12 h:12 h dark/light cycle, lights on at 06:00) with food and water access ad libitum. Experiments were carried out in compliance with the UK Animals (Scientific Procedures) Act 1986 and approved by the Lancaster University Animal Welfare and Ethics Review Board.

### ^14^C-2-Deoxyglucose Functional Brain Imaging

^14^C-2-deoxyglucose (^14^C-2-DG) functional brain imaging was conducted in accordance with published protocols (Dawson et al. 2015). In brief, local cerebral glucose utilization (LCGU) measurement was initiated by injection of ^14^C-2-DG (“intraperitoneally” [*i*.*p*.]), 4.625 MBq/kg in physiological saline at 2.5 mL/kg (American Radiolabelled Chemicals Inc.) 1 min after 25 mg/kg (R,S)-ketamine (Sigma-Aldrich) or 15 min after 5 mg/kg *d*-amphetamine (Sigma-Aldrich) administration (*i.p*. at 2 mL/kg in saline). Vehicle controls received saline vehicle administration (2 mL/kg *i.p.*) 1 or 15 min before ^14^C-2-DG injection (50% sample at each time). About 45 min after ^14^C-2-DG injection, animals were decapitated and a terminal blood sample collected, by torso inversion, into heparinized weigh boats. Plasma glucose concentrations (mmol/L) were detected from whole blood (Accu-Chek Aviva). The brain was then rapidly dissected out intact, frozen in isopentane (−40°C), and stored at (−80°C) until sectioning. Blood samples were centrifuged to isolate plasma, and plasma (20 μL) ^14^C concentrations were determined by liquid scintillation analysis (Packard). Frozen brains were sectioned (20 μm) in the coronal plane in a cryostat (−20°C). A series of three consecutive sections were retained from every 60 μm, thaw mounted onto slide covers, and rapidly dried on a hotplate (70°C). Autoradiograms were generated by apposing these sections, together with precalibrated ^14^C standards (39–1098 nCi/g tissue equivalents, American Radiolabelled Chemicals Inc) to X-ray film (Carestream BioMax MR film, Sigma-Aldrich, UK) for 7 days. Autoradiographic images were analyzed by a computer-based image analysis system (MCID/M5+, Interfocus). The local isotope concentration for each brain region of interest (RoI) was derived from the optical density of the autoradiographic images relative to that of the coexposed ^14^C standards. LCGU was determined in 59 brain regions of interest (RoI) across a range of neural systems (Supplementary Material, Supplementary Table 1) with reference to a stereotaxic mouse brain atlas (Paxinos and Franklin 2001). The rate of LCGU, in each RoI, was determined as the ratio of ^14^C present in that region relative to that of the whole brain ^14^C concentration in the same animal, referred to as the ^14^C-2-DG uptake ratio. Group sizes were WT: saline-treated *n* = 8 (male *n* = 5), ketamine-treated *n* = 9 (male *n* = 4), *d*-amphetamine-treated *n* = 9 (male *n* = 4); *Nrxn1a^+/−^*: saline-treated *n* = 10 (male *n* = 5), ketamine-treated *n* = 13 (male *n* = 6), *d*-amphetamine-treated *n* = 11 (male *n* = 6).

### Statistical Analysis

LCGU data were analyzed using ANOVA. In RoI where no significant genotype interactions (with sex or treatment) were detected, the main effect of genotype was accepted, using data from all experimental groups. For ketamine and *d*-amphetamine-induced effects, data were analyzed in two separate ANOVAs with sex, genotype, and treatment (saline, drug) as independent variables. Where significant interactions were found data were analyzed using post hoc pairwise *t*-test with Benjamini–Hochberg correction.

### Functional Brain Network Analysis

#### Global Network Properties and Regional Importance: Graph Theory Analysis

Global brain network properties and regional centrality were analyzed using data from saline-treated animals to avoid the confounding effect of drug treatment (Dawson et al. 2013, 2014a; Schwarz et al. 2007). The application of brain network analysis to ^14^C-2-DG imaging data has previously been described in detail (Dawson et al. 2012, 2013, 2014b). The algorithms applied allow us to define global brain network properties and the importance of each RoI in the network (regional centrality).

### Inter-regional Correlations and Functional Brain Networks

The inter-regional Pearson’s correlation coefficient was used as the metric of functional association between brain regions, generated from the ^14^C-2-DG uptake ratios for each RoI across all animals in the same experimental group (i.e., WT or *Nrxn1α^+/−^*). These correlations were Fisher *z*-transformed to give the data a more normal distribution. This resulted in the generation of a pair of {59 × 59} correlation matrices, each within-group matrix representing the specific association strength between each of the possible 1711 possible pairs of regions. From each correlation matrix (*R*), a binary adjacency matrix (*A*), where the functional connection between two regions (*a_i,j_* element) was zero if the Pearson’s correlation coefficient was lower than the defined threshold (*p_|i,j|_* < *T*) and unity if it was greater to or equal to the threshold (*p_|i,j|_* ≥ *T*), was generated. The adjacency matrix was then used for graph theory (network) analysis. The adjacency matrix can also be represented as an undirected graph *G*, where a line or edge represents the functional interaction between two RoI (also known as nodes) if the correlation coefficient exceeds the defined threshold value.

### Network Analysis

Network analysis was completed using the igraph package (Csardi and Nepsuz 2006) in R (R Core Team 2018). Network architecture was characterized at the global and regional scale, with global network architecture quantified in terms of the mean degree (<*k*>), average path length (*L*_p_), and mean clustering coefficient (*C*_p_) of the whole brain network. Regional properties were defined in terms of degree (*k_i_*), betweenness (*B_c_*), and eigenvector (*E_c_*) centrality. Global and regional metrics were determined on the binary adjacency matrices generated over a range of correlation thresholds (Pearson’s *r*, *T* = 0.49–0.59) that were selected on the basis that the maximum threshold yielded fully connected networks in each experimental group.

### Global Network Architecture

The degree of a network node (*k*), in this case a brain RoI, is simply the number of edges that connect the node to the network, with highly connected nodes having a high degree. The mean degree (<*k*>) is the average number of edges across all nodes in the network. A sparse network therefore has a low mean degree.

The minimum path length between two nodes in a graph (*L_i,j_*) is the smallest number of edges that must be traversed to make a connection between them. If two nodes are immediate neighbors, directly connected by a single edge, then *L_i,j_* = 1. The average path length (*L*_p_), or the average *L_i,j_* across all possible node pairs, is the average number of steps along the shortest paths across the network. This provides a measure of global network efficiency, where networks with low average path length are more efficient for information transfer.

The clustering coefficient of a node *i* (*C_i_*) is the ratio of the number of edges that exist between neighbors of that node relative to the maximum number of possible connections between them. This gives an indication of how well connected the neighborhood of a node is. The mean clustering coefficient (*C*_p_) is the average clustering of all nodes in the network, which provides a measure of local density, or the cliquishness, of the network. A high *C*_p_ suggests high clustering and so efficient local information transfer.

The significance of genotype-induced alterations in global network properties was determined by comparison of the real difference in each measure to that generated from 55 000 random permutations of the data (5000 permutations at each correlation threshold). Significance was set at *P* < 0.05.

### Regional Centrality

In this study, we considered node centrality in terms of degree (*k_i_*), betweenness (*B_c_*), and eigenvector (*E_c_*) centrality. Degree centrality (*k_i_*) simply measures the number of nodal connections (edges) a node has. Betweenness centrality (*B_c_*) is based on how many short paths go through a given node, with nodes having high betweenness centrality thus being more important in the network. Eigenvector centrality (*E_c_*) indicates the relative importance in the network of the nodes that the node of interest is connected to (i.e., the importance of a nodes connected neighbors) and thus gives an indication of a nodes influence in the network.

A brain region is considered to be an important network hub when it has a high k*_i_*, *B_c_* or *E_c_*. In this study, a RoI was defined as a hub in the brain network of the given experimental group if the regional centrality in the real network relative to that of calibrated Erdös–Rényi graphs (1000 graphs at each threshold, 11 000 in total) was *z* > 1.96 (Supplementary Material, Supplementary Table 2). The significance of genotype-induced alterations in regional centrality was determined by comparison of the *z-*score difference in the real networks to that in 55 000 random permutations of the real data (5000 per correlation threshold). Significance was set at *P* < 0.05.

### Regional Functional Connectivity: Partial Least Squares Regression

Following identification of *Nrxn1α^+/−^*-induced alterations in regional centrality (Table 1), we employed partial least squares regression (PLSR) analysis to determine the alterations in inter-regional connectivity underlying these observations. The application of PLSR to ^14^C-2DG imaging data has previously been outlined in detail (Dawson et al. 2013; Mouro et al. 2018) and was undertaken using the PLS package (Mevik and Wehrens 2007) in R. Significant regional connectivity to the “seed” RoI was considered to exist if the lower bound of the 95% confidence interval (CI) of the variable importance to the projection (VIP) statistic (estimated by jack-knifing) exceeded the 1.0 threshold. Altered connectivity in *Nrxn1a^+/−^* mice was determined by comparison of the VIP statistic to that in WT mice (t-test with Bonferroni correction). Lost connectivity was confirmed by a 95% VIP CI lower bound >1.0 in WT and <1.0 in *Nrxn1a^+/−^* mice, while gained connectivity was confirmed by a 95% VIP CI lower bound <1.0 in WT and >1.0 in *Nrxn1a^+/−^* mice.

**Table 1:** Regional centrality alterations in functional brain networks of *Nrxn1α^+/−^* mice

Brain region of interest (RoI)	Eigenvector (*E_c_*)	Betweenness (*B_c_*)	Degree (<*k_i_*>)
Prefrontal cortex			
Anterior prelimbic cortex (aPrL)	**−4.27** ^*^	−1.97	**−3.97** ^*^
Septum/DB			
Medial septum (MS)	**3.92** ^*^	0.60	2.07
Lateral septum (LS)	**3.31** ^*^	1.91	1.44
Vertical limb DB (VDB)	**4.08** ^*^	0.82	2.83
Horizontal limb DB (HDB)	**3.81** ^*^	−2.52	1.02
Thalamus			
Mediodorsal (MD)	**−3.67** ^*^	−0.83	−1.76
Centromedial (CM)	**−4.04** ^*^	−0.06	−3.27
Reuniens (Re)	**−3.69** ^*^	0.79	−0.69
Dorsal reticular (dRT)	**−3.86** ^*^	−0.69	−2.70
Ventral reticular (vRT)	**−3.57** ^*^	−0.96	−1.23
Basal ganglia			
Substantia nigra pars reticulata (SNR)	−0.30	**6.00** ^*^	2.41
Amygdala			
Central amygdala (CeA)	**2.89** ^*^	2.33	2.09
Dorsal hippocampus			
Cornu Ammonis 2 (CA2)	**3.37** ^*^	1.04	2.38
Mesolimbic system			
Nucleus accumbens shell (NacS)	**2.97** ^*^	1.91	1.84
Serotonergic system			
Median raphé (MR)	0.28	**6.85** ^*^	−0.84

Data shown as regional standardized z-score difference between Nrxn1alpha+/− and WT mice. Bold denotes a significant hub (+ve) or exteriority (−ve) region in the network of Nrxn1alpha+/− mice that does not have that status in WT controls. ^⋆^p<0.05 significant difference between Nrxn1alpha+/− and WT mice (55,000 data permutations).

As ketamine corrected thalamic metabolism in *Nrxn1a^+/−^* mice (Fig. 3), we employed PLSR analysis to characterize the impact of ketamine on the connectivity of these regions in *Nrxn1a^+/−^* mice. As these regions showed lost connectivity in saline-treated *Nrxn1a^+/−^* mice (Table 1, Fig. 2), we determined regional connectivity that was increased by ketamine administration in *Nrxn1a^+/−^* mice (*t*-test with Bonferroni correction and VIP 95% CI lower bound >1.0) and not significantly different to that seen in saline-treated WT mice (*t*-test with Bonferroni correction).

## Results

### *Nrxn1α* ^+/−^ Mice Show Thalamic, Mesolimbic, and Striatal Hypermetabolism with a Contrasting Cortical and Amygdala Hypometabolism

**Figure 1:**
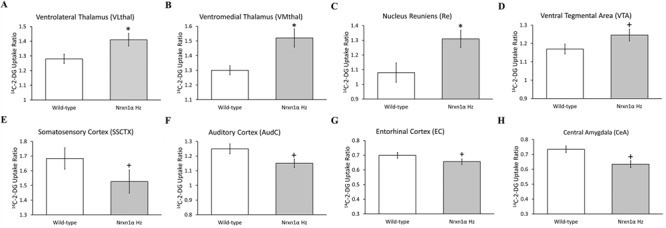
Constitutive cerebral metabolism is altered in the thalamus, mesolimbic system, cortex, and amygdala in *Nrxn1α*^+/−^ mice. *Nrxn1α*^+/−^ mice show hypermetabolism in the (*A*–*C*) thalamus and (*D*) ventral tegmental area with a contrasting hypometabolism in (*E*, *F*) primary sensory processing cortices, (*G*) entorhinal cortex, and (*H*) central amygdala. Data shown as mean ± SEM. ^+^*P* < 0.05 main effect of genotype, ANOVA. ^*^P < 0.05, pairwise *t*-test with Benjamini–Hochberg correction. VL, VM, and Re data from saline-treated animals only. VTA, SSCTX, AudC, EC, and CeA data are for the genotype effect across all treatment groups.

*Nrxn1α^+/−^* mice showed hypermetabolism in multiple thalamic nuclei, including the ventral reticular thalamus (vRT, *P* = 0.030), nucleus reuniens (Re, *P* = 0.007), and the ventrolateral (VL, *P* = 0.023) and ventromedial (VM, *P* = 0.005) thalamic nuclei (Fig. 1). *Nrxn1α^+/−^* mice also showed hypermetabolism in the ventral tegmental area (VTA, *F*_[1,54]_ = 4.21, *P* = 0.045) and ventromedial striatum (VMST, *F*_[1,54]_ = 4.59, *P* = 0.036).

By contrast, *Nrxn1α^+/−^* mice showed hypometabolism in select cortical regions, including primary sensory processing cortices (somatosensory (SSCTX, *F*_[1,54]_ = 5.20, *P* = 0.016) and auditory (AudC, *F*_[1,54]_ = 5.59, *P* = 0.022) cortex) and in the entorhinal cortex (EC, *F*_[1,53]_ = 4.50, *P* = 0.039), the cortical interface between the hippocampus and neocortex (Witter et al. 2017). In addition, *Nrxn1α^+/−^* mice showed hypometabolism in the central amygdala (CeA, *F*_[1,54]_ = 5.29, *P* = 0.025).

There was no evidence, in any RoI, that sex significantly influenced the impact of *Nrxn1α* heterozygosity on cerebral metabolism. Full data are shown in Supplementary Material, Supplementary Table 1.

### Average Path length (*L*_p_) Is Increased in Functional Brain Networks of *Nrxn1α*^+/−^ Mice

**Figure 2:**
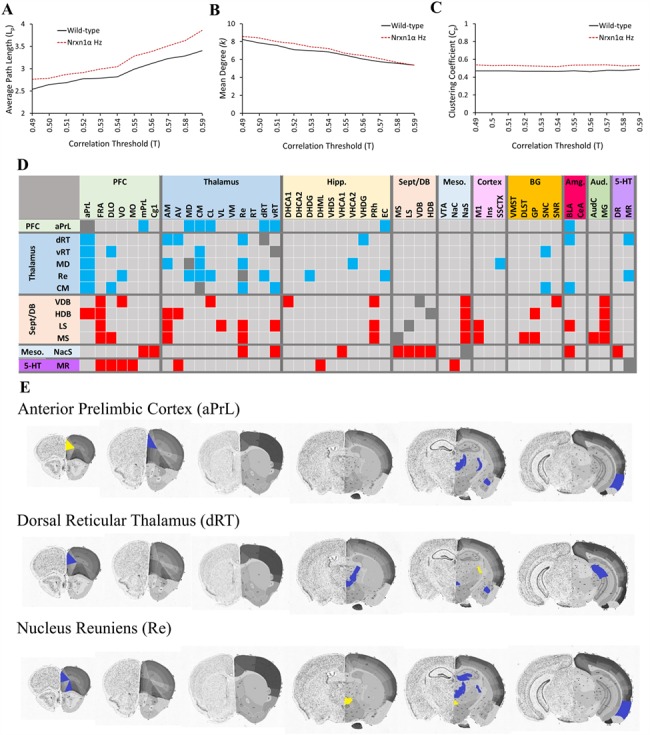
*Nrxn1α* heterozygosity alters functional brain network structure and inter-regional functional connectivity. (*A*) Average path length is significantly increased (*L*_p_, *P* = 0.041) in functional AQ9 brain networks of *Nrxn1α*^+/−^ mice, while (*B*) mean degree (<*k*>, *P* = 0.589) and (*C*) the global clustering coefficient (*C*_p_, *P* = 0.277) are not altered. Inter-regional functional connectivity alterations in *Nrxn1α*^+/−^ mice support reduced thalamic “rich club,” thalamic-PFC and abnormal septum/DB and raphé-PFC connectivity. (*D*) Heatmap showing significantly lost (blue) and abnormal/gained (red) inter-regional connectivity in *Nrxn1α*^+/−^ relative to WT mice, determined by comparison of the VIP statistic (*t*-test with Bonferroni correction) calculated through PLSR analysis. (*E*) Brain images showing the anatomical localization of altered inter-regional connectivity for the anterior prelimbic cortex (aPrL), dorsal reticular thalamus (dRT), and nucleus reuniens (Re) “seed” regions (yellow). Blue denotes functional connectivity present in WT mice (VIP 95% CI > 1.0) that is significantly lost in *Nrxn1α*^+/−^ mice (VIP 95% CI < 1.0, and *P* < 0.05 *t*-test with Bonferroni correction). 5-HT, serotonergic system; Amg, amygdala; Aud, auditory system; BG, basal ganglia; Hipp, hippocampus; Meso, mesolimbic system; PFC, prefrontal cortex; Sept/DB: septum/diagonal band of Broca. Brain images adapted from the Allen mouse brain atlas (mouse.brain-map.org/static/atlas).

In terms of global brain network properties, we found that the average path length (*L*_p_) was significantly increased (*P* = 0.041) in functional brain networks of *Nrxn1α^+/−^* mice (Fig. 2), suggesting that the ability of information to transmit across brain networks is significantly reduced in *Nrxn1α^+/−^* mice. We found no evidence that the number of connections (mean degree (<*k*>), *P* = 0.589) or clustering (clustering coefficient (*C*_p_), *P* = 0.277) was altered in functional brain networks of *Nrxn1α^+/−^* mice.

### Brain Region Importance Is Altered in Functional Brain Networks of *Nrxn1α*^+/−^ Mice

Through centrality analysis, we identified significant alterations in regional importance in the functional brain networks of *Nrxn1α^+/−^* mice (Table 1). Multiple thalamic regions, including the mediodorsal (MD), centromedial (CM), Re, dorsal reticular thalamus (dRT), and vRT, showed reduced centrality in *Nrxn1α^+/−^* mice (*E_c_*). The anterior prelimbic cortex (aPrL) also showed reduced centrality in *Nrxn1α^+/−^* mice (<*k_i_*> and *E_c_*).

By contrast, all nuclei of the septum/diagonal band (DB) of Broca system (lateral septum (LS), medial septum (MS), vertical (VDB) and horizontal (HDB) limbs of the diagonal band of Broca) showed increased centrality in *Nrxn1α^+/−^* mice (*E_c_*). The *E_c_* of the central amygdala (CeA), CA2 subfield of the dorsal hippocampus (DHCA2), the globus pallidus (GP), and nucleus accumbens shell (NacS) was also significantly increased in *Nrxn1α^+/−^* mice, while the substantia nigra pars reticulata (SNR) and serotonergic median raphé (MR) also showed increased *B_c_* in brain networks of *Nrxn1α^+/−^* mice (Table 1). Full data are shown in Supplementary Table 2.

### Compromised Thalamic “Rich-Club,” Thalamic-PFC and Abnormal Septum/DB, Mesolimbic and Raphé-PFC Connectivity in *Nrxn1α*^+/−^ Mice

Rich club architecture in brain networks indicates that highly connected regions (hubs) are also highly connected to each other. In saline-treated WT mice, centrality analysis identified multiple thalamic regions (MD, dRT, VL) as significant hubs (Supplementary Material, Supplementary Table 2). PLSR analysis in these animals identified functional connectivity between thalamic hubs in these animals (Supplementary Tables 4–8), supporting the rich club status of these thalamic nuclei, as previously reported in rats (Dawson et al. 2012) and humans (van den Heuvel and Sporns 2011). The rich club nature of thalamic nuclei in saline-treated WT mice was further confirmed through the application of algorithms specifically assessing rich club structure (Ma and Mondragon 2015). Using these algorithms, multiple thalamic nuclei (dRT, vRT, Re, CM, CL) and PFC subfields (aPrL, Cg1) were identified as members of the rich club core (RCC) in functional brain networks of saline-treated WT mice. These regions were not part of the RCC in saline-treated *Nrxn1a^+/−^* mice (supplemental material, RCC analysis). PLSR analysis confirmed lost interconnectivity between these thalamic nuclei in *Nrxn1α^+/−^* mice (Fig. 2), supporting compromised thalamic “rich club” connectivity in these animals. We also found significant evidence for lost PFC-thalamus functional connectivity in *Nrxn1α^+/−^* mice (Fig. 2). These reductions in inter-regional and “rich club” connectivity would contribute to the increased network average path length (Fig. 2) seen in *Nrxn1α^+/−^* mice.

PLSR analysis also identified abnormal inter-regional connectivity in *Nrxn1α^+/−^* mice that is not present in WT animals, contributing to the increased centrality of selected brain regions in the *Nrxn1α^+/−^* mice (Table 1). This included abnormal connectivity between the septum/DB and the PFC, thalamus, mesolimbic and auditory systems in *Nrxn1α^+/−^* mice that was not present in WT controls. In addition, *Nrxn1α^+/−^* mice had connectivity between the medial raphé (MR) and PFC that was not present in WT controls (Fig. 2). Full data are shown in Supplementary Tables 3–18.

### Subanesthetic Ketamine Administration Normalizes Thalamic Hyperactivity in *Nrxn1α*^+/−^ Mice

**Figure 3:**
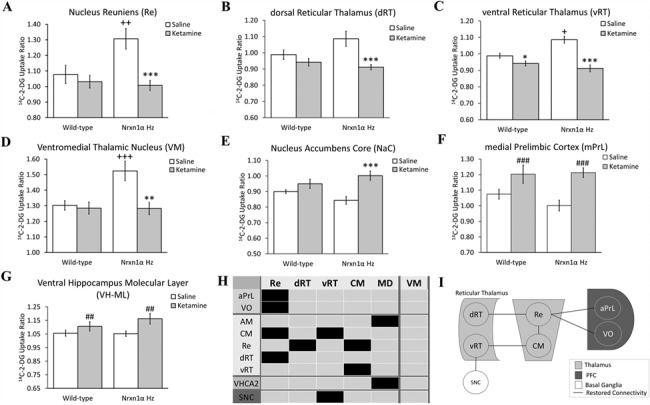
Subanesthetic ketamine administration normalizes thalamic metabolism and restores thalamic hub and reticular thalamus–nucleus reuniens–prefrontal cortex (RT–Re–PFC) circuit connectivity in *Nrxn1α*^+/−^ mice. (*A–D*) Subanesthetic ketamine administration normalizes thalamic hyperactivity in *Nrxn1α*^+/−^ mice. (*E*) *Nrxn1α*^+/−^ mice show an enhanced cerebral metabolic response to ketamine in the nucleus accumbens core (NacC). (*F*, *G*) The impact of ketamine on PFC and hippocampal function is not altered in *Nrxn1α*^+/−^ mice. Data shown as mean ± SEM. *Nrxn1α* Hz = *Nrxn1α*^+/−^ mice. ^*^P < 0.05, ^**^P < 0.01 and ^***^P < 0.001 ketamine effect within genotype and ^++^*P* < 0.01, ^+++^*P* < 0.001 genotype effect within treatment group (*t*-test with BH correction). ^##^*P* < 0.01, ^###^*P* < 0.001 ketamine effect (ANOVA) in regions where no significant genotype × treatment interaction found. (*H*) Summary connectivity map showing the functional connections of thalamic hub regions lost in *Nrxn1α*^+/−^ mice that are restored by ketamine administration. Black shading denotes lost connectivity in saline-treated *Nrxn1α*^+/−^ mice that is restored to the level seen in wild-type mice in ketamine-treated *Nrxn1α*^+/−^ mice. (*I*) Summary diagram of RT–Re–PFC circuit connectivity restored in *Nrxn1α*^+/−^ mice by subanesthetic ketamine administration. aPrL, anterior prelimbic cortex; CM, centromedial thalamus; dRT, dorsal reticular thalamus; MD, mediodorsal thalamus; Re, nucleus reuniens; VHCA2, ventral hippocampus CA2; VO, ventral orbital cortex; vRT, ventral reticular thalamus; SNC, substantia nigra pars compacta. Representative autoradiograms are shown in Supplementary Fig. 1.

In line with previous reports, ketamine administration increased LCGU in the PFC, hippocampus and striatum while reducing LCGU in thalamic nuclei (Dawson et al. 2013, 2015). Subanesthetic ketamine administration effectively reversed the constitutive thalamic hypermetabolism seen in *Nrxn1α^+/−^* mice, bringing metabolism to a similar level to that seen in WT controls and WT mice treated with ketamine (Fig. 3). Saline-treated *Nrxn1α^+/−^* mice displayed thalamic hyperactivity as compared to saline-treated WT animals in the VM (*P* = 0.005), Re (*P* = 0.007), vRT (*P* = 0.030), and dRT (trend, *P* = 0.062). While ketamine reduced LCGU in these regions in *Nrxn1α^+/−^* mice (VM, *P* = 0.002; Re, *P* < 0.001; dRT, *P* < 0.001; vRT, *P* < 0.001), it was not significantly altered in WT mice (VM, *P* = 0.961; Re, *P* = 0.650; dRT, *P* = 0.388), with the exception of the vRT (*P* = 0.018) where ketamine also reduced LCGU in WT mice. LCGU in these regions was not significantly different in ketamine-treated *Nrxn1α^+/−^* mice as compared to saline-treated WT mice (VM, *P* = 0.961; Re, *P* = 0.486; dRT, *P* = 0.123) with the exception of the vRT, which was significantly different from that in saline (*P* = 0.007) but not ketamine-treated (*P* = 0.800) WT mice. These effects were supported by a significant genotype × treatment interaction in each region (VM, *F*_[1,32]_ = 6.472, *P* = 0.016; Re, *F*_[1,32]_ = 6.703, *P* = 0.014; dRT, *F*_[1,32]_ = 5.057, *P* = 0.035; vRT, *F*_[1,32]_ = 4.246, *P* = 0.048).

In addition to these changes, ketamine increased LCGU in the nucleus accumbens core (NacC) of *Nrxn1α^+/−^* mice (*P* < 0.001) but not in WT animals (*P* = 0.234), with NacC metabolism in ketamine-treated *Nrxn1α^+/−^* mice being higher than that in saline-treated WT animals (*P* = 0.025). The altered NacC metabolic response to ketamine in *Nrxn1α*^+/−^ mice was supported by a significant genotype × treatment interaction (*F*_[1,32]_ = 4.527, *P* = 0.041).

In contrast to these modified responses, the impact of ketamine on LCGU in all PFC and hippocampal RoI was not altered in *Nrxn1α*^+/−^ mice (Fig. 3). Full data are available in Supplementary Table 1.

### Subanesthetic Ketamine Administration Restores Thalamic “Rich Club” Hub and Reticular Thalamus–Nucleus Reuniens–Prefrontal (RT–Re–PFC) Functional Connectivity in *Nrxn1α*^+/−^ Mice

To elucidate the impact of ketamine administration on thalamic connectivity in *Nrxn1α^+/−^* mice, we employed PLSR analysis to characterize the inter-regional functional connectivity of thalamic “rich club” regions showing decreased connectivity in saline-treated *Nrxn1α^+/−^* mice (Table 1, Fig. 2; Re, dRT, vRT, MD, CM). Given the ability of ketamine administration to normalize LCGU in thalamic regions in *Nrxn1α^+/−^* mice (Fig. 3), we determined the lost connectivity present in saline-treated *Nrxn1α^+/−^* mice restored by ketamine administration.

In *Nrxn1α^+/−^* mice, ketamine restored inter-regional functional connectivity between thalamic “rich club” brain regions and between the Re-PFC, effectively restoring connectivity in the reticular thalamus–nucleus reuniens–prefrontal cortex circuit (RT-Re-PFC, Fig. 3). In *Nrxn1α^+/−^* mice, ketamine also increased dRT-Re functional connectivity, bringing it to a similar level to that seen in WT control mice, re-establishing functional connectivity between these regions. Similarly, for the Re, ketamine increased connectivity to the CM thalamic nucleus and two PFC subfields (aPrL, VO) in *Nrxn1α^+/−^* mice, restoring it to a similar level to that seen in WT controls. For the vRT, ketamine increased functional connectivity to the CM and SNC in *Nrxn1α^+/−^* mice, restoring connectivity to a level similar to that seen in WT controls. When the CM was considered as the seed region in PLSR analysis, the restoration of functional connectivity to the Re and vRT was confirmed. Finally, when the MD was considered as the seed region, ketamine increased connectivity to the AM thalamic nucleus and CA2 subfield of the ventral hippocampus (VHCA2), restoring connectivity to a similar level seen in WT controls. Full PLSR connectivity data are shown in Supplementary Tables 3–18 and Supplementary Fig. 2.

Overall, these data suggest that subanesthetic ketamine administration effectively restores functional connectivity between thalamic “rich club” regions in *Nrxn1α^+/−^* mice and re-establishes thalamic-PFC functional connectivity (Re, Fig. 3). This suggestion is further supported by the observation that these thalamic nuclei (dRT, vRT, Re, MD, CM) form part of the RCC in ketamine-treated, but not saline-treated, *Nrxn1α^+/−^* mice (Supplemental Material, RCC analysis).

### The Cerebral Metabolic Response to Dextro-Amphetamine Is Not Altered in *Nrxn1α*^+/−^ Mice

**Figure 4:**
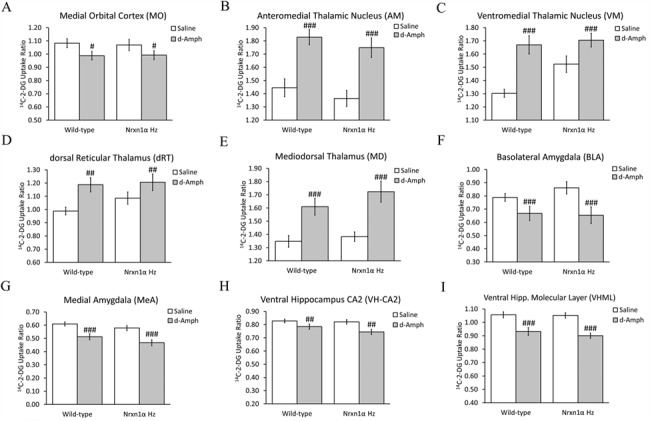
Cerebral metabolic responses to d-amphetamine are not altered in *Nrxn1α*^+/−^ mice. Data shown as mean ± SEM of the ^14^C-2-DG uptake ratio. d-Amphetamine (d-Amph) administration induces (*A*) medial orbital cortex hypometabolism, (*B*–*E*) thalamic hypermetabolism, and (*F*, *G*) amygdala and (*H*, *I*) hippocampal hypometabolism. We found no evidence, in any brain region where d-amphetamine modified cerebral metabolism, that the response was altered in *Nrxn1α*^+/−^ mice. Data shown as mean ± SEM. ^#^*P* < 0.05, ^##^*P* < 0.01 and ^###^*P* < 0.001 significant effect of d-amphetamine (ANOVA).

In keeping with previous observations, *d*-amphetamine administration induced hypometabolism in the PFC, amygdala, and ventral hippocampus along with hypermetabolism in the thalamus, substantia nigra (pars compacta, SNC), and retrosplenial (RSC) cortex (Ernst et al. 1997; Miyamoto et al. 2000). We found no evidence, in any RoI, that the LCGU response to *d*-amphetamine was significantly altered in *Nrxn1α^+/−^* mice (Fig. 4). Full data are shown in Supplementary Table 1.

## Discussion

We have, for the first time, identified the alterations in constitutive cerebral metabolism and functional brain network structure that result from heterozygous *Nrxn1α* deletion. These alterations have translational relevance to neurodevelopmental disorders for which *NRXN1* deletions are a genetic risk factor, including ASD and ScZ, and to individuals with 2p16.3 (*NRXN1*) deletions. The data show that ketamine administration can restore the thalamic metabolism and dysconnectivity, including the compromised thalamic “rich club” and RT–Re–PFC connectivity, which result from heterozygous *Nrxn1α* deletion. This suggests that altered NMDA-R activity may underlie the thalamic dysfunction seen in *Nrxn1α*^+/−^ mice, although this requires further investigation. Overall, the data suggest that ketamine administration and NMDA-R antagonism may be an effective strategy to restore the thalamic-PFC dysfunction that results from 2p16.3 (*NRXN1*) deletion. This may also have translational relevance to the reported therapeutic benefit of NMDA-R antagonists in ASD, although further characterization in *Nrxn1α*^+/−^ mice would be needed to establish this more firmly.

### Translational Relevance of the Brain Network Connectivity Deficits Present in *Nrxn1α*^+/−^ Mice

The alterations in cerebral metabolism and brain network connectivity seen in *Nrxn1α^+/−^* mice may contribute to the reported behavioral alterations seen in these animals and have translational relevance to the functional brain imaging deficits seen in ASD and ScZ.

Behavioral deficits previously reported in *Nrxn1α^+/−^* mice include hyperactivity, altered habituation, deficits in object recognition and social memory, and deficits in associative learning (as measured by passive avoidance) (Laarakker et al. 2012; Dachtler et al. 2015). While behavioral deficits in *Nrxn1α^+/−^* mice are not always found (Grayton et al. 2013), the published data generally support deficits in associative learning and recognition learning and memory as a consequence of *Nrxn1α* heterozygosity. In rodents, the neural circuitry for object recognition memory includes the PFC, hippocampus, thalamus, perirhinal cortex, and entorhinal cortex (Antunes and Biala 2012; Warburton and Brown 2015), while the neural circuitry for rodent social recognition includes the PFC, hippocampus, septum, and amygdala (Bicks et al. 2015). Intriguingly, we found evidence for regional metabolic dysfunction and altered connectivity in *Nrxn1α^+/−^* mice within these neural systems, which could contribute to the behavioral deficits seen in these animals. This includes entorhinal cortex (EC) and central amygdala (CeA) nucleus hypofunction along with altered thalamic-PFC and septum-PFC connectivity (Fig. 2). In addition, we also found evidence for compromised nucleus reuniens (Re) connectivity to both the PFC and hippocampus in *Nrxn1α^+/−^* mice. While a direct glutamatergic projection exists from the hippocampus to the PFC, information from the PFC to the hippocampus is relayed through the nucleus reuniens (Vertes 2002). Thus, compromised connectivity in the PFC–Re–hippocampal circuit in *Nrxn1α^+/−^* mice could contribute to the deficits in learning and memory seen in these animals. Interestingly, deficits in the functional connectivity of this neural circuit have been reported in another mouse model of genetic risk for ASD and ScZ (*Disc1,*Dawson et al. 2015), suggesting that this may be a common neural pathway affected by genetic mutations associated with ASD and ScZ.

We also found evidence for increased activity in motor thalamic nuclei (VL and VM) in *Nrxn1α^+/−^* mice, and our ketamine data suggest that NMDA-R dysfunction may contribute to the increased neuronal activity of these motor thalamic regions (Fig. 3). The VL and VM, along with other motor areas, including the cerebellum, basal ganglia, and motor cortex, play a key role in motor learning in rodents (Ding et al. 2002; Jeljeli et al. 2003). Intriguingly, we found evidence for enhanced motor learning abilities in *Nrxn1α^+/−^* mice in the accelerating rotarod test (Supplementary Material, Supplementary Fig. 3), which mirrors that previously reported in *Nrxn1α* knockout mice (Etherton et al. 2009). This suggests that enhanced neuronal activity and NMDA-R function in motor circuitry may contribute to the enhanced motor learning abilities of *Nrxn1α^+/−^* mice.

The alterations in brain function and network connectivity seen in *Nrxn1α^+/−^* mice may also have translational relevance to the functional brain imaging deficits seen in ASD and ScZ. For example, thalamic dysfunction is widely supported in both ASD and ScZ with thalamic hypofunction rather than hyperfunction, as seen in *Nrxn1α^+/−^* mice, generally reported (Buchsbaum et al. 1996; Hazlett et al. 2004; Haznedar et al. 2006). However, in human brain imaging studies, the small and discrete thalamic nuclei identified as hyperactive in *Nrxn1α^+/−^* mice, including the RT and Re, have not previously been resolved. Moreover, recent studies with greater anatomical resolution have identified complex patterns of thalamic dysfunction, including both regional hypoactivation and hyperactivation, in these disorders, dependent on the thalamic nuclei characterized and the cognitive state of patients during testing (Pergola et al. 2015; Mitelman et al. 2018). Interestingly, while studies of brain function in ASD rodent models are limited, the thalamic hyperfunction seen in *Nrxn1α^+/−^* mice parallels that recently reported in another rodent model relevant to the disorder (Cho et al. 2017). Other alterations in cerebral metabolism present in *Nrxn1α^+/−^* mice with translational alignment to those reported in ASD and ScZ include amygdala (CeA) and entorhinal cortex (EC, brodmans 28/34) hypofunction, which parallels the temporal lobe hypofunction and the direct hypofunction of these structures in these disorders (Carina Gilberg et al. 1993; Zakzanis et al. 2000; Aoki et al. 2015; Mitelman et al. 2018).

The data suggest that the RT is a primary locus of thalamic dysfunction as a consequence of *Nrxn1α* heterozygosity, evidenced by altered RT metabolism and connectivity in *Nrxn1α^+/−^* mice. While direct evidence from functional brain imaging studies to support RT dysfunction in ASD and ScZ is currently lacking, a range of evidence supports RT dysfunction in these disorders. For example, the RT plays a key role in processes dysfunctional in both ASD and ScZ, including sleep, the generation of brain oscillations, sensory integration, and cognition (Pratt and Morris 2015; Krol et al. 2018). Recent cellular evidence directly supports RT dysfunction in ScZ (Steullet et al. 2018), while direct evidence for RT dysfunction in ASD is currently lacking. However, a primary cell type in the RT are parvalbumin positive (PV+) interneurons (Tanahira et al. 2009), and PV+ expression is altered in both ASD (Lawrence et al. 2010; Hashemi et al. 2017) and ScZ (Beasley and Reynolds 1997; Zhang and Reynolds 2002). While these studies have characterized PV+ cells in the PFC and hippocampus, recent studies have also confirmed RT PV+ cell dysfunction in ScZ (Steullet et al. 2018). This remains to be characterized in ASD. However, the RT and PV+ neurons are known to be dysfunctional in other preclinical rodent models relevant to ASD and ScZ, including models involving NMDA-R dysfunction (Cochran et al. 2003; Dawson et al. 2012, 2015; Wells et al. 2016). Moreover, as NMDA-R hypofunction is able to induce both RT PV+ cell dysfunction and RT hypometabolism (Cochran et al. 2003; Dawson et al. 2012, 2013), and NMDA-Rs directly regulate the activity of PV+ neurons (Carlen et al. 2012; Miller et al. 2016), PV+ neuron dysfunction may contribute to the NMDA-R dependent thalamic dysfunction seen in *Nrxn1α^+/−^* mice. This suggestion is further supported by the observation that parvalbumin directly regulates neuronal activity in the RT (Alberi et al. 2012). As other thalamic (VPM, VPL) and cortical (SSCTX) regions found to be dysfunctional in *Nrxn1α^+/−^* mice also contain high levels of PV+ cells (Tanahira et al. 2009), dysfunction of this cell type may also contribute to the altered metabolism seen in these brain regions. Thus, given the potential translational relevance of PV+ cell deficits in *Nrxn1α^+/−^* mice, the possible contribution of PV+ cell dysfunction to the RT and other brain imaging deficits seen in *Nrxn1α^+/−^* mice certainly warrants further systematic investigation.

While *Nrxn1a* heterozygosity did not reproduce the overt PFC hypometabolism (hypofrontality) reported in ASD and ScZ (Ohnishi et al. 2000; Hill et al. 2004; Mitelman et al. 2018), PFC functional connectivity was compromised in *Nrxn1α^+/−^* mice. This mirrors the reduced PFC connectivity reported in ASD and ScZ and in other relevant genetic mouse models relevant to these disorders (Sigurdsson et al. 2010; Bertero et al. 2018; Liska et al. 2018). In *Nrxn1α^+/−^* mice, this includes reduced thalamic-PFC connectivity, mirroring the reduced functional and structural thalamic-PFC connectivity reported in ASD and ScZ (Nair et al. 2013; Woodward and Heckers 2016; Giraldo-Chica and Woodward 2017; Woodward et al. 2017). The reduced interconnectivity of thalamic nuclei in *Nrxn1α^+/−^* mice, contributing to the loss of thalamic “rich club” hubs, also parallels the decreased interconnectivity between thalamic nuclei in ASD (Tomasi and Volkow 2019) and ScZ (Tomasi and Volkow 2014) and mirrors the loss of brain network hubs in these disorders (Rubinov and Bullmore 2013; Itahashi et al. 2014). We also found broader evidence for alterations in functional brain network structure in *Nrxn1α^+/−^* mice that mirror those seen in ASD and ScZ. This includes an increase in the average path length of functional (Micheloyannis et al. 2006; Barttfeld et al. 2012; Boersma et al. 2013; Itahashi et al. 2014) and structural brain networks (Roine et al. 2015; Yeo et al. 2016) in these disorders, supporting decreased efficiency of information transfer across brain networks as a consequence of *Nrxn1α* heterozygosity and in these disorders. Moreover, the hypoconnectivity of functional brain networks in *Nrxn1α^+/−^* mice parallels that reported in other preclinical models relevant to ASD (Bertero et al. 2018; Liska et al. 2018) and ScZ (Dawson et al. 2014b).

### *Nrxn1α* Heterozygosity Alters In vivo Glutamate but Not General Monoaminergic Neurotransmitter System Function

Our data suggest that *Nrxn1α* heterozygosity induces NMDA-R dysfunction that contributes to disturbed neuronal activity in selected neural circuits, including the mesolimbic system, posterior thalamic nuclei (VM and VL), and the RT–Re–PFC circuit (Fig. 3). Intriguingly, our data show that administration of a subanesthetic dose of the NMDA-R antagonist ketamine corrects the thalamic hypermetabolism and RT–Re–PFC dysfunction present in *Nrxn1α^+/−^* mice. The regulation of glutamatergic and NMDA-R function by Nrxn1α is supported by a diverse range of studies (Chubykin et al. 2007; Barrow et al. 2009; de Wit et al. 2009; Etherton et al. 2009; Espinoza et al. 2015; Dai et al. 2019). The role of NMDA-R dysfunction in ASD is complex, with both NMDA-R agonists and antagonists reported as improving ASD symptoms (Lee et al. 2015). NMDA-R antagonists, such as Memantine and Amantadine, have been shown to have positive therapeutic effects in ASD (Chez et al. 2007; Ghaleiha et al. 2013; Nikvarz et al. 2017). Our data suggest that these effects may be mediated through the correction of abnormal thalamic and mesolimbic function and the restoration of the RT-Re-PFC circuit and that thalamic hyperactivity in individuals with ASD may offer a biomarker for NMDA-R antagonist efficacy in the disorder. This conjecture certainly warrants further systematic investigation. While our data suggest that the correction of thalamic hypermetabolism in *Nrxn1α^+/−^* mice by ketamine administration may have translational relevance to the therapeutic impact of NMDA-R antagonists in ASD, these effects would need to be confirmed in relation to the behavioral alterations seen in *Nrxn1α^+/−^* mice (Laaraker et al. 2012; Dachtler et al. 2015). Two concerns with regard to behavioral testing using the dose of ketamine applied in our imaging study are its ability to induce locomotor hyperactivity (Irifune et al. 1991), which is likely to impact on performance in many behavioral tests, and its ability to disrupt PFC and hippocampal function in *Nrxn1α^+/−^* mice (Supplementary Table 1), which is likely to disrupt behaviors dependent upon these neural systems, including several of the behaviors reported to be altered in *Nrxn1α^+/−^* mice (Laaraker et al. 2012; Dachtler et al. 2015). Thus, future studies should be dedicated to testing the efficacy of lower doses of ketamine, in relation to both the behavioral and brain imaging alterations seen in *Nrxn1α^+/−^* mice, identifying doses that do not significantly disrupt locomotor activity but may act to restore behaviors and thalamic function in these animals.

Another limitation to our study is the mixed pharmacology of ketamine, which not only acts as an NMDA-R antagonist but also displays biological activity at other targets including hyperpolarization-activated cyclic nucleotide-gated channels (HCN), cholinergic receptors, dopamine-2 receptors (D_2_R), opioid receptors, and voltage-gated sodium channels (VGSCs). The actions of ketamine are further complicated by the complex biological activity of its metabolites in vivo (Zanos et al. 2018). Thus, the suggestion that the ability of ketamine to restore thalamic function in *Nrxn1α^+/−^* mice relies on its activity at NMDA-Rs is made with caution, and further molecular characterization is needed to more strongly support this contention. Nevertheless, studies showing that *Nrxn1α* influences NMDA-R function further support the plausibility of this mechanism (Chubykin et al. 2007; Barrow et al. 2009; de Wit et al. 2009; Etherton et al. 2009; Espinoza et al. 2015; Dai et al. 2019).

We also found that general monoaminergic neurotransmitter system functional responses, as evidenced by the LCGU response to *d*-amphetamine, were not altered in *Nrxn1α^+/−^* mice (Fig. 4). This suggests that *Nrxn1α* heterozygosity does not reproduce the enhanced response to *d*-amphetamine seen in ScZ (Breier et al. 1997; Swerdlow et al. 2018) and that the impact on general monoaminergic system function is limited. However, the suggestion that these systems are not altered in *Nrxn1α^+/−^* mice is made with caution. Whether the function of the individual monoaminergic systems (dopamine, serotonin, noradrenaline) is altered in *Nrxn1α^+/−^* mice remains to be adequately tested. In fact, our observation of increased functional connectivity between the serotonergic median raphé (MR) and PFC in *Nrxn1α^+/−^* mice (Fig. 2) suggests that serotonergic neurotransmitter system function may be altered in these animals.

Interestingly, stimulants including *d*-amphetamine are used to treat ADHD-like symptoms (impaired attention, hyperactivity and impulsivity) in ASD (Nickels et al. 2008; Cortese et al. 2012). However, we found no evidence that *d*-amphetamine normalized any of the dysfunctional cerebral metabolism present in *Nrxn1α^+/−^* mice. While hyperactivity is reported in *Nrxn1α^+/−^* mice (Laarakker et al. 2012), impaired attention or impulsivity is yet to be adequately tested. Our data suggest that *Nrxn1α^+/−^* mice may not provide a useful model of stimulant-based treatment of ADHD-like symptoms in ASD. However, given the ability of ketamine to restore RT–Re–PFC circuit function in *Nrxn1α^+/−^* mice, a key circuit involved in attentional processing (Prasad et al. 2013; Kim et al. 2016; Wells et al. 2016), NMDA-R antagonists may be able to restore attentional deficits, if found to be present in these animals. Thus, there appears to be a degree of pharmacological selectivity with regard to the potential predictive utility of *Nrxn1α^+/−^* mice as a model of therapeutic efficacy in ASD.

## Conclusion

In conclusion, we have identified a range of brain imaging functional and connectivity deficits in *Nrxn1α^+/−^* mice that have translational relevance to those seen in ASD and ScZ. This includes the hyperactivity and dysconnectivity of multiple thalamic nuclei that are partially normalized by subanesthetic ketamine administration. Thus, *Nrxn1α*^+/−^ mice may provide a translational model relevant to the therapeutic efficacy of NMDA-R antagonists in ASD, while their translational utility in relation to stimulant compounds in ASD appears to be limited.

## Notes

Conflict of Interest: None declared.

## Funding

Faculty of Health and Medicine, Lancaster University PhD studentship (to N.D. and S.J.B.) and by The Royal Society (RG140134 to N.D.). ND is also supported by the UK Medical Research Council (MR/N012704/1).

## Supplementary Material

Supplemental_bhz244

Figure_S1_bhz244

Figure_S2_bhz244

Figure_S3_bhz244

Table_S1_bhz244

Table_S2_bhz244

Tables_S3_bhz244
