# Supplementary material for: Ketamine Restores Thalamic-Prefrontal Cortex Functional Connectivity in a Mouse Model of Neurodevelopmental Disorder-Associated 2p16.3 Deletion
Source: Cereb Cortex. 2019 Dec 8;30(4):2358–71. doi: 10.1093/cercor/bhz244 (PMC7175007; doi:10.1093/cercor/bhz244)
Supplement: Supplemental_bhz244 [file supplemental_bhz244.pdf]

Supplement Material : Characterisation of Rich Club Core (RCC) Structure in Functional Brain Networks of *Nrxn1α*$^{+/-}$ Mice

Members of the Rich club core (RCC) in functional brain networks were identified using the Rich Core algorithm developed by Ma & Mondragon, 2015 implemented in the “brainGraph” package (Watson, 2018) in R (R Core Team, 2018 https://www.R-project.org/). This algorithm allows the identification of those regions/nodes in the brain network that form part of a core of brain regions that are part of the rich club (have a high degree and are highly connected to other brain regions with a high degree).

In wild-type (WT) mice treated with saline the RCC identified consisted of thalamic (6 out of 19 regions), prefrontal cortex (3 out of 19 regions) and hippocampal regions (3 out of 19 regions, Table S19). The median raphé (MR) and components of the auditory system (MG, AudC) were also identified as being members of the RCC in functional brain networks in saline-treated WT animals.

In *Neurexin-1α* heterozygous (*Nrxn1α*$^{+/-}$) mice treated with saline the RCC consisted largely of regions of the septum/DB, with all 4 septum/DB regions analysed being identified as part of the RCC in these animals. There was little overlap between the regions identified as being part of the RCC in saline-treated *Nrxn1α*$^{+/-}$ mice and those seen in saline-treated WT mice, with only the centrolateral thalamic nucleus (CL) being identified as a member of the RCC in both groups. The thalamic and prefrontal cortex regions identified as being part of the RCC in WT animals treated with saline were not members of the RCC in the functional brain networks of saline treated *Nrxn1α*$^{+/-}$ mice (Table S19).

In *Nrxn1α*$^{+/-}$ mice treated with ketamine more brain regions were identified as being part of the RCC (Table S19). Members of the RCC seen in WT mice treated with saline were also identified as part of the RCC in *Nrxn1α* $^{+/-}$ mice treated with ketamine, including multiple thalamic regions (MD, dRT, CM, Re, CL, vRT) and four subfields of the hippocampus (ML, DG, EC, PRh). These regions were not present as members of the RCC in saline-treated *Nrxn1α*$^{+/-}$ mice.

In WT mice treated with ketamine many more hippocampal brain regions (9 out of 16 hippocampal regions, Table S19) were identified as being part of the RCC than were seen in saline treated WT mice and more prefrontal cortex (PFC) regions were also identified as members of the RCC in functional brain networks of ketamine treated WT mice (6 regions; VO, IL, mPrL, aPrL, Cg1, FRA). This aligns with the reported ability of subanaesthetic ketamine administration to enhance hippocampal and PFC functional connectivity in WT mice (Dawson et al. 2013, 2015). Interestingly, in contrast to observations seen in ketamine-treated *Nrxn1α*$^{+/-}$ mice, the dRT, CM, Re, CL and vRT were not identified as being members of the RCC in ketamine treated WT mice, suggesting that ketamine's ability to restore the RCC membership of these thalamic regions was specific to *Nrxn1α*$^{+/-}$ mice.

Table S19. Rich club core (RCC) details for functional brain networks in *Nrxn1α*$^{+/-}$ mice and wild-type controls

Experimental Group	Core Size (no. of regions)	Rank	K.r	Members
Wild-type Saline	0.328 (19)	19	7	MD (12), dRT (11), CM (9), Re (9), aPrL (8), Cg1 (8), CL (8), BLA (8), DHML (8), AudC (8), MG (8), DG (8), ML (8), MR (8), mPrL (7), M1 (7), Piri (7), HDB (7), vRT (7)
Wild-type Ketamine	0.448 (26)	26	8	DHML (13), DS (13), VO (12), MS (12), CA1 (12), ML (12), IL (11), DLST (11), mPrL (10), AM (10), MD (10), MeA (10), Hab (10), CA2 (10), MB (10), PRh (10), aPrL (10), Cg1 (9), SSCTX (9), MG (9), DG (9), FRA (9), RSC (8), DHCA2 (8), CA3(8), SNC(8)
Nrxn1α$^{+/-}$ Saline	0.190 (11)	11	8	VDB (13), MS (10), HDB (10), CeA (10), DHCA2 (10), NaS (9), AM (9), AV (9), CL (9), Ins (8), LS (8)
Nrxn1α$^{+/-}$ Ketamine	0.483 (28)	28	4	CA2 (9), SNC (9), ML (8), PRh (8), EC (8), vRT (7), BLA (7), DHCA1 (7), DG (7), NaS (6), Ins (6), CM (6), CL (6), dRT (6), MeA (6), SNR (6), AV (6), MD (5), VM (5), Re (5), DS (5), CA3 (5), IL (4), M1 (4), DLST (4), DHML (4), MG (4), CA1 (4)

Rich club core (RCC) membership is altered in Nrxn1α $^{+/-}$ mice, and partially restored by subanaesthetic ketamine administration. Core size indicates the size of the rich club core (RCC) relative to the whole network, with the number of nodes included in the core (out of a total of 58 brain regions) shown in parenthesis. Regions (nodes) are ranked on the basis of their degree (number of connections). In the table Rank indicates the rank node position, with nodes ordered from greatest degree to the least, that is considered to be at the boundary of the RCC. K.r indicates the degree of the node at the boundary of the RCC. Members denotes those brain regions considered to be part of the RCC. Regions are presented in order of their rank in the network and with their degree in parenthesis. Functional brain networks were analysed at a threshold of T=0.59 using the Rich core algorithm as outlined in Ma & Mondragon, 2015. For region identities see supplemental Table S1.

To further characterise Rich Club membership we calculated the rich club coefficient (ϕ) [6] and used this to generate subgraphs showing rich club membership for the different groups (Figure S2). The results gained using this approach are closely aligned with those gained through the characterisation of the RCC (Table S19). Network summary statistics are shown in Table S20 with the Rich Club subgraphs shown in Figure S2.

Table S20. Summary metrics for the Rich Club subgraphs shown in Figure S2.

**Experimental Group**	Rich Club Subgraph	Rich Club Coefficient (ϕ)	N_k	E_k
Wild-type Saline	Fig 1A	0.308	14	28
Nrxn1α$^{+/-}$ Saline	Fig 1B	0.639	9	23
Nrxn1α$^{+/-}$ Ketamine	Fig 1C	0.229	22	53
Wild-type Ketamine	Fig 1D	0.371	21	78
Nrxn1α$^{+/-}$ Saline	Fig 1E	0.410	15	43
Nrxn1α$^{+/-}$ Ketamine	Fig 1F	1	5	10
Wild-type Ketamine	Fig 1G	0.298	26	97

Calculated on the basis of the minimum node degree identified as the boundary cut off degree by RCC analysis (Table S19, Figures 1 A-D) and in subgraphs with a similar degree threshold to that applied in Wild-type mice treated with saline (Figure S2 E-G). The Rich club coefficient (φ) indicates the magnitude of rich club-ness within the subgraphs, and indicates that each of these display a rich club structure. N_k denotes the number of regions included in the rich club subgraph and E_k the number of connections/edges.

Figure S2. Rich Club subgraphs from the different experimental groups

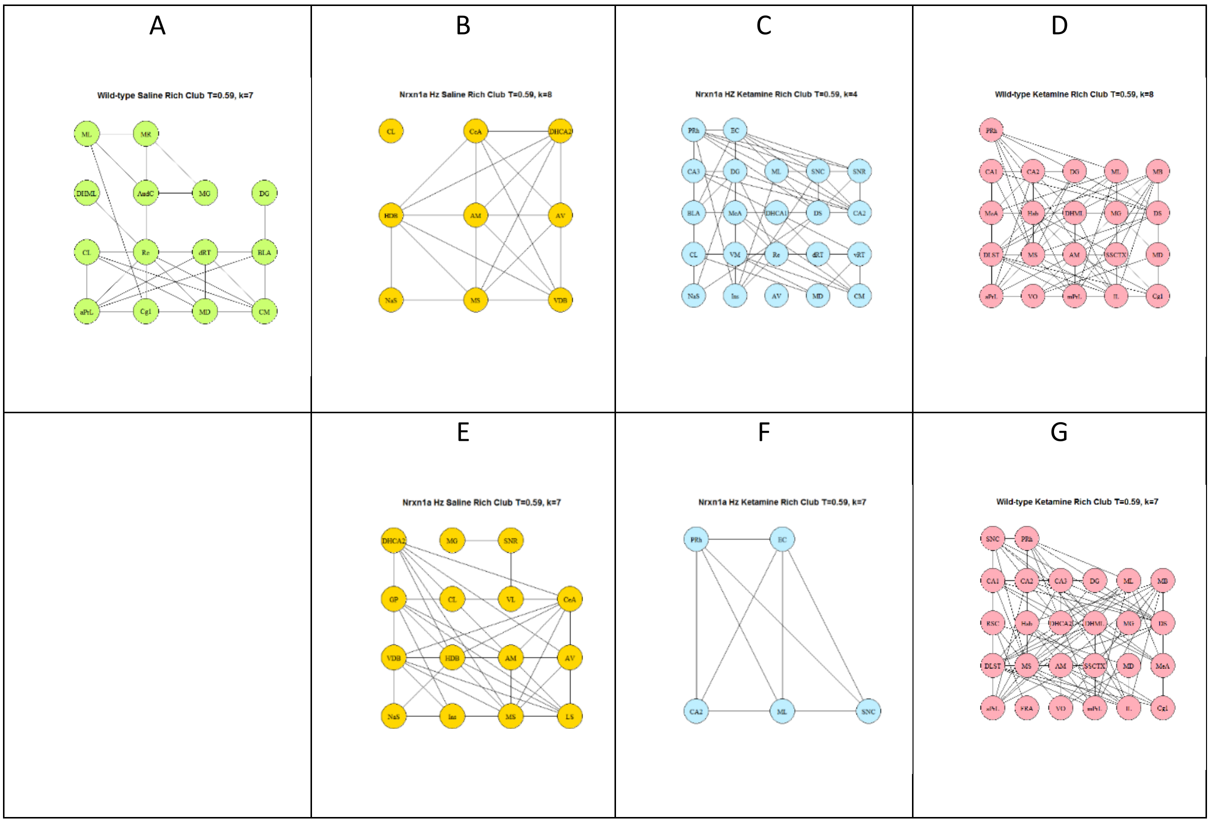

Regions included in the rich club subgraphs of functional brain networks from the different experimental groups. All networks were threshold at T=0.59. In ***A-D*** *the minimum degree (k) used for the inclusion of a brain region was based on that gained for the boundary vertex between the rich club and non-rich club nodes, calculated by the RCC algorithm (Table S19). In* ***E-G****, the rich club subgraphs gained in Nrxn1α$^{+/-}$ (Nrxn1α$^{+/-}$ Hz) mice at the same degree threshold applied in saline-treated WT mice (k=7) is shown for direct comparison. Rich club subgraphs included regions previously identified through the application of the RCC algorithm, indicating that thalamic-prefrontral regions form a rich club core in WT mice, but not in Nrxn1α$^{+/-}$ mice (saline-treated), and that the membership of these regions to the rich club is restored by ketamine administration in Nrxn1α$^{+/-}$ mice. By contrast, these regions do not form part of the rich club in ketamine treated WT mice. For region identities see supplemental Table S1.*

References

Dawson N, Morris BJ, Pratt JA. 2013. Subanaesthetic ketamine treatment alters prefrontal cortex connectivity with thalamus and ascending subcortical systems. Schizophr Bull. 39: 366.

Dawson N, McDonald M, Higham DJ, Morris BJ, Pratt JA. 2015. Subanaesthetic ketamine treatment promotes abnormal interactions between neural subsystems and alters the properties of functional brain networks. Neuropsychopharmacology. 39: 1786-1798.

Ma A, Mondragon RJ. 2015. Rich cores in networks. PLoS One. 10: e0119678.

R Core Team. 2018. R: A language and environment for statistical computing. R Foundation for Statistical Computing, Vienna, Austria. URL https://www.R-project.org/.

Watson CG. 2018. brainGraph: graph theory analysis of brain MRI data. R package version 2.2.0. https://CRAN.R-project.org/package=brainGraph.

Zhou S, Mondragon RJ. 2004. The rich-club phenomenon in the internet topology. IEEE Comm Lett. 8: 180-182.
