# Supplementary material for: Ketamine Restores Thalamic-Prefrontal Cortex Functional Connectivity in a Mouse Model of Neurodevelopmental Disorder-Associated 2p16.3 Deletion
Source: Cereb Cortex. 2019 Dec 8;30(4):2358–71. doi: 10.1093/cercor/bhz244 (PMC7175007; doi:10.1093/cercor/bhz244)
Supplement: Figure_S1_bhz244 [file figure_s1_bhz244.pdf]

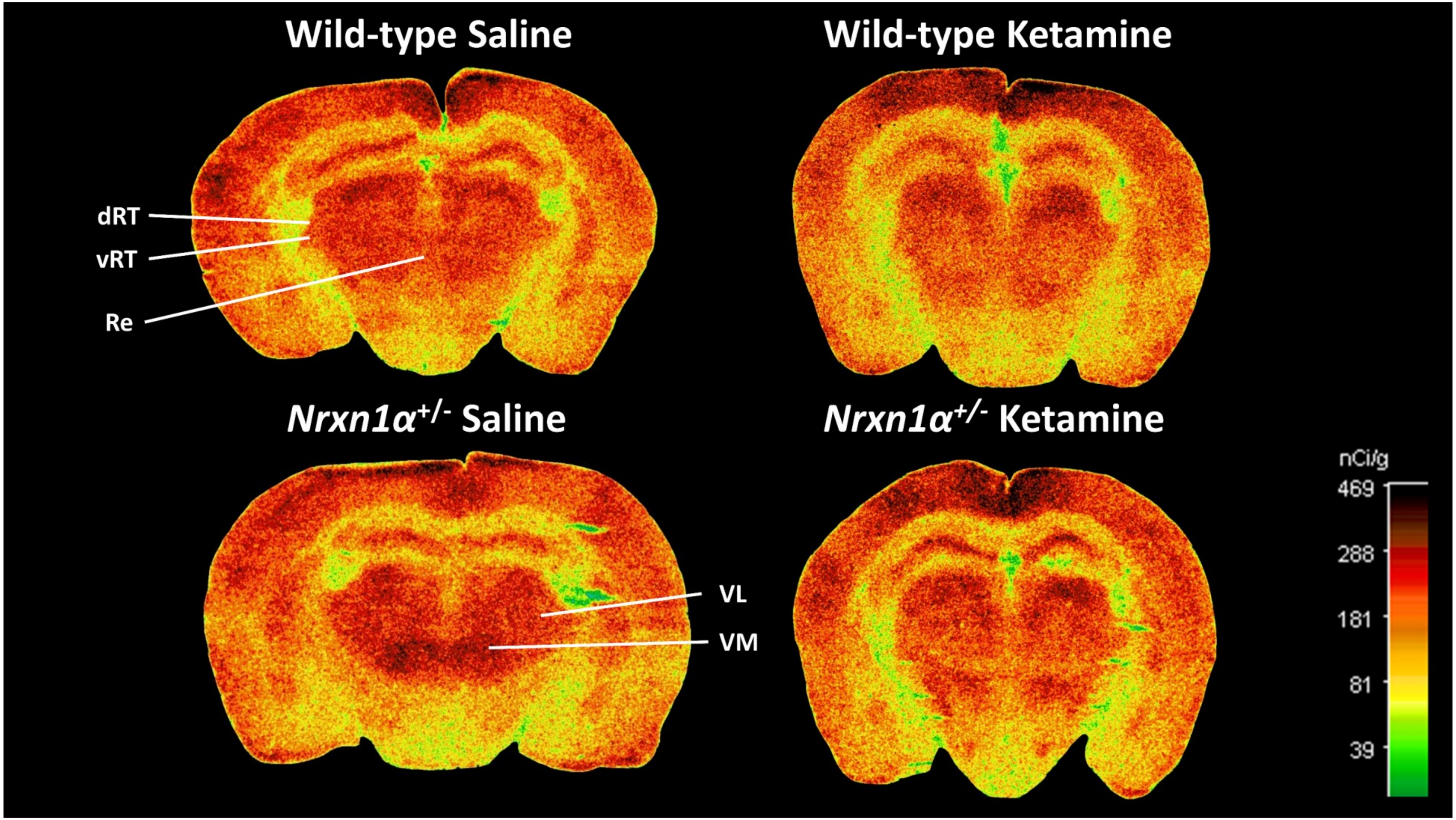

Figure S1. Representative pseudocolour autoradiograms at the level of the dorsal hippocampus showing tissue ^{14}C-2-deoxyglucose concentrations (nCi/g) in ketamine-treated and saline-treated *Nrxn1α*$^{+/-}$ mice and wild-type controls. *Nrxn1α*$^{+/-}$ mice show higher rates of glucose metabolism (reflected by higher tissue ^{14}C-2-deoxyglucose levels) in several thalamic nuclei that is reduced by ketamine (25mg/kg, *i.p.*) administration. Warm colours (red/dark orange) show higher levels of tissue ^{14}C-2-deoxyglucose levels and cooler colours (green/yellow) denote lower levels of tissue ^{14}C-2-deoxyglucose. Animals for the images shown had similar whole brain average ^{14}C-2-deoxyglucose concentrations (Wild-type Saline = 232 nCi/g; Wild-type Ketamine = 238 nCi/g, *Nrxn1α*$^{+/-}$ Saline = 234 nCi/g; *Nrxn1α*$^{+/-}$ Ketamine = 231 nCi/g). dRT = dorsal reticular thalamus, Re = nucleus reuniens, VL = ventrolateral thalamus, VM = ventromedial thalamus, vRT = ventral reticular thalamus.
