# Supplementary material for: Ketamine Restores Thalamic-Prefrontal Cortex Functional Connectivity in a Mouse Model of Neurodevelopmental Disorder-Associated 2p16.3 Deletion
Source: Cereb Cortex. 2019 Dec 8;30(4):2358–71. doi: 10.1093/cercor/bhz244 (PMC7175007; doi:10.1093/cercor/bhz244)
Supplement: Figure_S2_bhz244 [file figure_s2_bhz244.pdf]

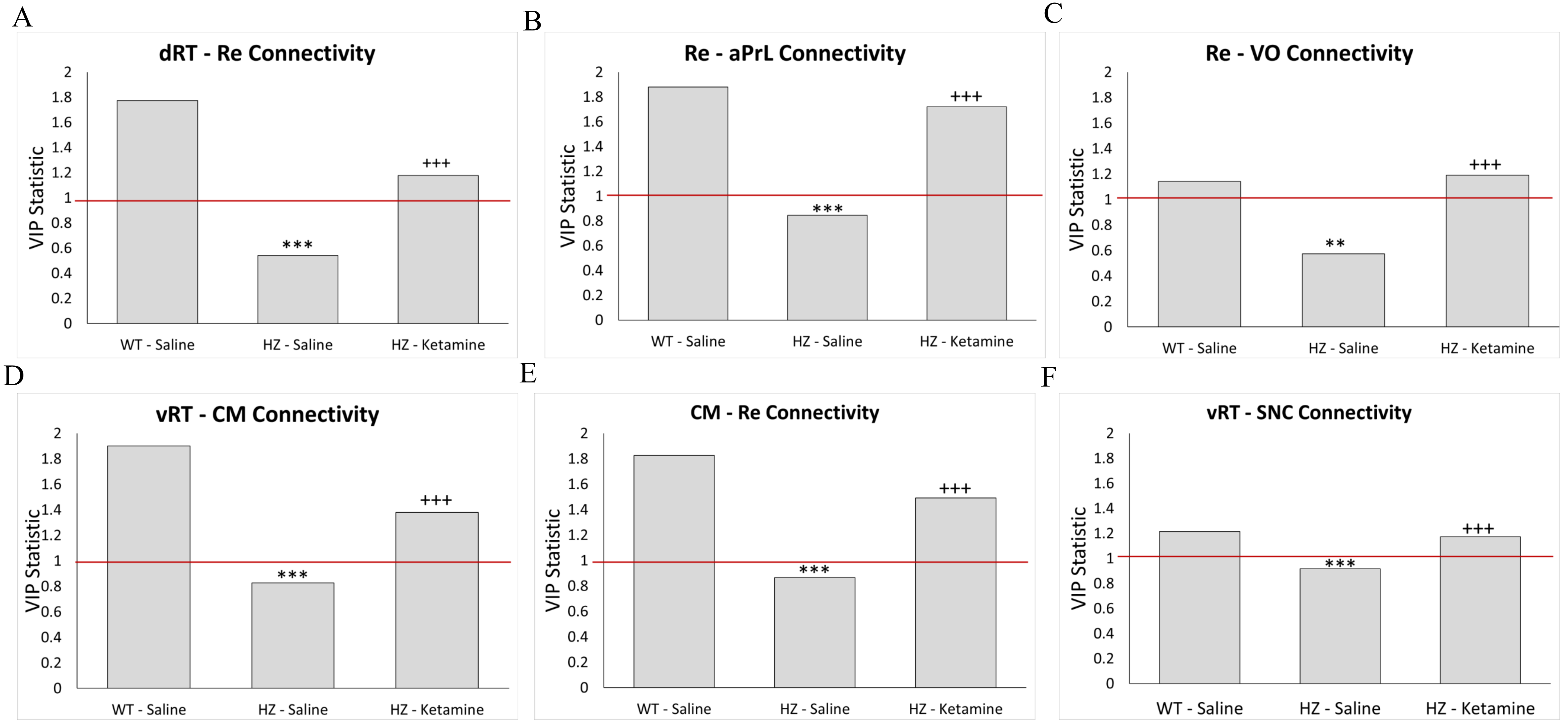

Figure S2. Subanaesthetic ketamine administration restores thalamic hub and reticular-reuniens-prefrontal cortex (RT-Re-PFC) circuit functional connectivity in *Nrxn1α*$^{+/-}$ mice. (A-F) Subanaesthetic Ketamine administration restores inter-regional connectivity of thalamic hubs in *Nrxn1α*$^{+/-}$ mice. Data shown for selected functional connections restored in the RT-Re-PFC circuit. Data shown as the lower bound of the 95% CI (estimated by Jack-Knifing) of the VIP statistic. VIP > 1.0 (red line) denotes a significant functional connection. **p<0.01, ***p<0.001 significant difference from wild-type saline-treated animals (WT – Saline). $^{+++}$p<0.001 significant difference from saline-treated *Nrxn1α*$^{+/-}$ mice (HZ – Saline, t-test with Bonferroni correction). aPrL=anterior prelimbic cortex, CM=centromedial thalamus, dRT=dorsal reticular thalamus, Re=nucleus reuniens, vRT=ventral reticular thalamus, SNC=substantia nigra pars compacta. Full data shown in Tables S3-S18.
