# Supplementary material for: Ketamine Restores Thalamic-Prefrontal Cortex Functional Connectivity in a Mouse Model of Neurodevelopmental Disorder-Associated 2p16.3 Deletion
Source: Cereb Cortex. 2019 Dec 8;30(4):2358–71. doi: 10.1093/cercor/bhz244 (PMC7175007; doi:10.1093/cercor/bhz244)
Supplement: Figure_S3_bhz244 [file figure_s3_bhz244.pdf]

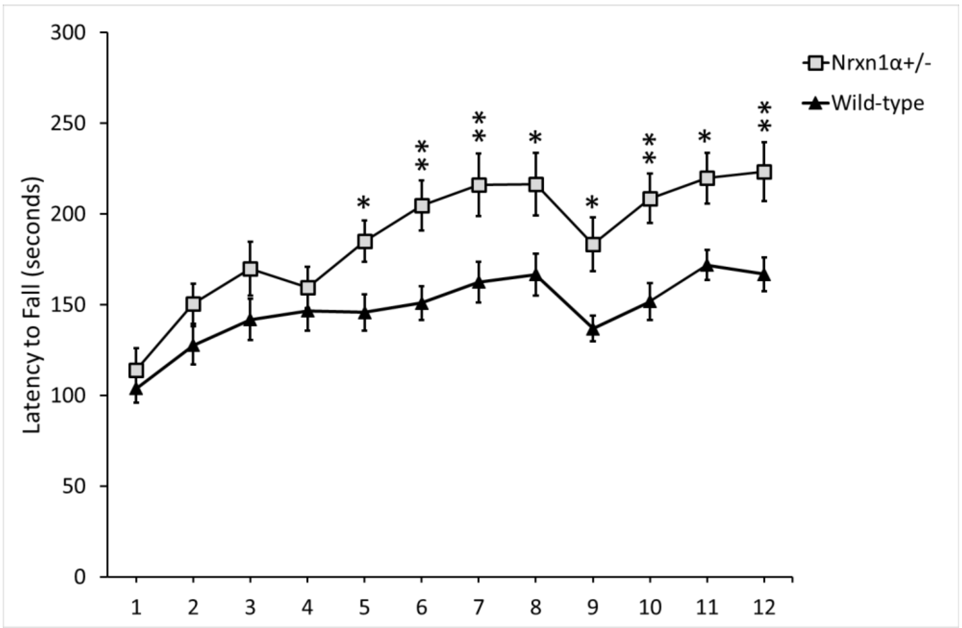

Figure S3. *Nrxn1α$^{+/-}$* mice show enhanced motor locomotor learning in comparison to wild-type controls. Data shown as Mean ± SEM latency to fall from an accelerating rotarod (4-45 rpm in 5 minutes). Mice were tested in 12 trials, with four trials per day and a 5 minute inter-trial interval between trials conducted on the same day. The time to fall off or to turn one full revolution was measured, as outlined in previous studies (Etherton et al. 2009. PNAS. 42:17998-18003). Data was analysed using repeated measures ANOVA with a significant genotype x trial interaction supported ($F_{(11,405)}$=1.974, p=0.0297), along with a significant sex x trial interaction ($F_{(11,405)}$=2.596, p=0.003) and a significant effect of trial itself ($F_{(11,405)}$=16.557, p<0.001). *Post-hoc* testing confirmed that while initial locomotor abilities were not significantly different between *Nrxn1α$^{+/-}$* and wild-type mice (trials 1-4), there was a significant difference between the two genotypes by trial 5, with *Nrxn1α$^{+/-}$* mice showing a greater latency to fall. This difference persisted across all remaining trials. *P<0.05, **P<0.01 significant difference from wild-type (t-test with Bonferonni-Holm correction). *Nrxn1α$^{+/-}$* n=19 (male n=6), wild-type n=23 (male=12).
