# Supplementary material for: Ketamine Restores Thalamic-Prefrontal Cortex Functional Connectivity in a Mouse Model of Neurodevelopmental Disorder-Associated 2p16.3 Deletion
Source: Cereb Cortex. 2019 Dec 8;30(4):2358–71. doi: 10.1093/cercor/bhz244 (PMC7175007; doi:10.1093/cercor/bhz244)
Supplement: Table_S1_bhz244 [file table_s1_bhz244.pdf]

Genotype / Treatment	Wild-type Saline Mean	SE	Neurexin1α Hz Saline Mean	SE	Wild-type Ketamine Mean	SE	Neurexin1α Hz Ketamine Mean	SE	Ketamine main effect sign.	Wild-type d-Amphetamine Mean	SE	Neurexin1α Hz d-Amphetamine Mean	SE	d-Amphetamine main effect sign.	Wild-type ALL Treatments Mean	SE	Neurexin1α Hz ALL Treatments Mean	SE
**Prefrontal Cortex**																		
anterior Prelimbic Cortex (aPrL)	1.12	± 0.02	1.09	± 0.02	1.27	± 0.03	1.29	± 0.03	p<0.001	1.12	± 0.03	1.10	± 0.03		1.17	± 0.02	1.17	± 0.02
Frontal Association Area (FRA)	1.20	± 0.02	1.23	± 0.03	1.21	± 0.03	1.22	± 0.04		1.16	± 0.02	1.17	± 0.10		1.19	± 0.02	1.21	± 0.03
Dorsolateral Orbital Cortex (DLO)	0.93	± 0.02	0.94	± 0.05	1.02	± 0.03	1.00	± 0.02	p=0.039	0.84	± 0.03	0.79	± 0.03	p=0.002	0.93	± 0.02	0.91	± 0.02
Ventral Orbital Cortex (VO)	1.69	± 0.05	1.60	± 0.05	1.72	± 0.05	1.67	± 0.04		1.61	± 0.04	1.69	± 0.06		1.67	± 0.03	1.66	± 0.03
Medial Orbital Cortex (MO)	1.08	± 0.03	1.07	± 0.04	1.19	± 0.04	1.13	± 0.03	p=0.041	0.99	± 0.03	0.99	± 0.03	p=0.032	1.09	± 0.03	1.07	± 0.02
medial Prelimbic Cortex (mPrL)	1.07	± 0.03	1.00	± 0.03	1.20	± 0.06	1.21	± 0.03	p<0.001	0.98	± 0.04	0.99	± 0.04		1.09	± 0.03	1.08	± 0.03
Infralimbic Cortex (IL)	0.95	± 0.02	0.88	± 0.02	1.04	± 0.03	1.00	± 0.02	p<0.001	0.84	± 0.03	0.84	± 0.02	p=0.007	0.94	± 0.02	0.91	± 0.02
Cingualte Cortex (Cg1)	1.23	± 0.04	1.20	± 0.03	1.31	± 0.02	1.32	± 0.02	p<0.001	1.16	± 0.05	1.15	± 0.05		1.23	± 0.03	1.23	± 0.02
**Thalamus**																		
Anteromedial Thalamus (AM)	1.44	± 0.07	1.36	± 0.06	1.37	± 0.04	1.41	± 0.03		1.83	± 0.06	1.75	± 0.07	p<0.001	1.55	± 0.05	1.51	± 0.04
Anteroventral Thalamus (AV)	1.76	± 0.08	1.72	± 0.11	1.58	± 0.05	1.69	± 0.04		2.40	± 0.09	2.48	± 0.12	p<0.001	1.92	± 0.08	1.95	± 0.08
Mediodoersal Thalamus (MD)	1.35	± 0.04	1.38	± 0.04	1.27	± 0.03	1.26	± 0.02	p=0.003	1.61	± 0.06	1.72	± 0.08	p<0.001	1.41	± 0.04	1.45	± 0.04
Centromedial Thalamus (CM)	1.04	± 0.03	1.13	± 0.05	1.01	± 0.03	0.99	± 0.03	p=0.032	1.22	± 0.07	1.13	± 0.06		1.09	± 0.03	1.08	± 0.03
Centrolateral Thalamus (CL)	1.11	± 0.03	1.16	± 0.03	1.15	± 0.02	1.17	± 0.03		1.23	± 0.06	1.21	± 0.04		1.17	± 0.02	1.18	± 0.02
Ventrolateral Thalamus (VL)	1.28	± 0.03	1.41*	± 0.04	$1.15^{\$}$	± 0.02	$1.17^{\$\$\$\#}$	± 0.03	p<0.001	1.78	± 0.12	1.99	± 0.09	p<0.001	1.41	± 0.07	1.51	± 0.07
Ventromedial Thalamus (VM)	1.30	± 0.03	1.52*	± 0.06	1.28	± 0.04	$1.28^{\$\$\#}$	± 0.04	p<0.003	1.67	± 0.07	1.71	± 0.05	p<0.001	1.42	± 0.05	1.49	± 0.04
Nucleus Reuniens (Re)	1.08	± 0.06	1.31*	± 0.07	1.03	± 0.04	$1.01^{\$\$\$\#}$	± 0.03	p<0.001	1.40	± 0.14	1.29	± 0.12		1.17	± 0.06	1.19	± 0.05
anterior Reticular Thalamus (RT)	1.30	± 0.03	1.44	± 0.05	1.14	± 0.04	1.13	± 0.03	p<0.001	1.66	± 0.11	1.80	± 0.09	p<0.001	1.37	± 0.06	1.44	± 0.06
dorsal Reticular Thalamus (dRT)	0.99	± 0.03	1.09	± 0.05	0.94	± 0.02	$0.91^{\$\$\$\#}$	± 0.02	p<0.001	1.19	± 0.05	1.21	± 0.06	p=0.005	1.04	± 0.03	1.06	± 0.03
ventral Reticular Thalamus (vRT)	1.00	± 0.03	1.10*	± 0.04	$0.89^{\$}$	± 0.01	$0.89^{\$\$\$\#}$	± 0.02	p<0.001	1.14	± 0.04	1.15	± 0.04	p=0.026	1.01	± 0.03	1.03	± 0.03
Medial Geniculate (MG)	1.22	± 0.06	1.20	± 0.03	1.02	± 0.02	1.06	± 0.03	p<0.001	1.21	± 0.03	1.24	± 0.04		1.15	± 0.03	1.16	± 0.02
**Mesolimbic**																		
Ventral Tegmental Area (VTA)	1.14	± 0.04	1.24	± 0.05	1.09	± 0.01	1.14	± 0.02	p=0.036	1.28	± 0.05	1.38	± 0.06	p=0.026	1.17	± 0.03	1.25^{+}	± 0.03
Nucleus Accumbens Core (NaC)	0.90	± 0.01	0.84	± 0.03	0.95	± 0.03	$1.00^{\$\$\$\#}$	± 0.03		0.82	± 0.02	0.80	± 0.03	p=0.016	0.89	± 0.02	0.89	± 0.02
Nucleus Accumbens Shell (NaS)	1.02	± 0.02	1.00	± 0.02	1.05	± 0.02	1.07	± 0.02	p=0.004	0.91	± 0.03	0.91	± 0.02	p<0.001	0.99	± 0.02	1.00	± 0.02
**Cortex**																		
Motor Cortex (M1)	1.37	± 0.04	1.42	± 0.04	1.18	± 0.04	1.19	± 0.04	p<0.001	1.24	± 0.04	1.24	± 0.06	p=0.002	1.26	± 0.03	1.27	± 0.03
Piriform Cortex (Piri)	1.60	± 0.05	1.61	± 0.04	1.41	± 0.04	1.43	± 0.04	p<0.001	1.50	± 0.04	1.45	± 0.04	p=0.005	1.50	± 0.03	1.49	± 0.03
Insular Cortex (Ins)	0.83	± 0.01	0.81	± 0.03	0.92	± 0.01	0.89	± 0.02	p<0.001	0.72	± 0.02	0.70	± 0.02	p<0.001	0.83	± 0.02	0.80	± 0.02
Somatosensory Cortex (SSCTX)	1.41	± 0.05	1.61	± 0.08	1.22	± 0.02	1.31	± 0.04	p<0.001	1.94	± 0.11	2.19	± 0.11	p<0.001	1.53	± 0.07	1.68^{+}	± 0.08
Retrosplenial Cortex (RSC)	1.64	± 0.06	1.63	± 0.08	1.61	± 0.06	1.60	± 0.04		1.75	± 0.05	1.89	± 0.11	p=0.022	1.67	± 0.03	1.70	± 0.05
Perirhinal Cortex (PRh)	0.89	± 0.02	0.87	± 0.01	1.01	± 0.03	0.99	± 0.04	p<0.001	0.79	± 0.02	0.77	± 0.01	p<0.001	0.90	± 0.02	0.88	± 0.02
Entorhinal Cortex (EC)	0.74	± 0.01	0.67	± 0.02	0.76	± 0.03	0.73	± 0.04		0.61	± 0.02	0.55	± 0.01	p<0.001	0.70	± 0.02	0.66^{+}	± 0.02
Auditory Cortex (AudC)	1.39	± 0.07	1.20	± 0.06	1.14	± 0.03	1.15	± 0.04	p=0.015	1.24	± 0.05	1.11	± 0.05		1.25	± 0.03	1.15^{+}	± 0.03
**Basal Ganglia**																		
Ventromedial Striatum (VMST)	1.22	± 0.01	1.25	± 0.03	1.23	± 0.02	1.28	± 0.02		1.25	± 0.03	1.31	± 0.03		1.23	± 0.01	1.28^{+}	± 0.02
Dorsolateral Striatum (DLST)	1.32	± 0.03	1.35	± 0.04	1.30	± 0.02	1.33	± 0.02		1.24	± 0.03	1.28	± 0.03	p=0.047	1.29	± 0.02	1.32	± 0.02
Globus Pallidus (GP)	0.85	± 0.02	0.90	± 0.02	0.78	± 0.01	0.82	± 0.02	p<0.001	0.91	± 0.02	0.89	± 0.01		0.85	± 0.01	0.86	± 0.01
Substantia Nigra pars Compacta (SNC)	0.80	± 0.04	0.75	± 0.02	0.74	± 0.02	0.72	± 0.02		0.87	± 0.05	0.93	± 0.05	p=0.006	0.80	± 0.02	0.80	± 0.02
Substantia Nigra pars Reticulata (SNR)	0.91	± 0.04	0.96	± 0.03	0.85	± 0.02	0.86	± 0.02	p=0.012	1.01	± 0.05	1.01	± 0.05		0.92	± 0.03	0.94	± 0.02
**Septum/Diagonal Band of Broca**																		
Medial Septum (MS)	0.90	± 0.02	0.92	± 0.03	0.87	± 0.02	0.87	± 0.02		0.99	± 0.03	0.96	± 0.03	p=0.045	0.92	± 0.02	0.91	± 0.01
Lateral Septum (LS)	0.92	± 0.02	0.89	± 0.02	0.77	± 0.02	0.78	± 0.02	p<0.001	1.00	± 0.02	0.96	± 0.02	p=0.001	0.90	± 0.02	0.87	± 0.02
Ventral Limb of the Diagonal Band of Broca (VDB)	0.92	± 0.03	0.95	± 0.05	0.77	± 0.03	0.79	± 0.02	p<0.001	0.88	± 0.04	0.89	± 0.04		0.86	± 0.02	0.87	± 0.02
Horizontal Limb of the Diagonal Band of Broca (HDB)	1.00	± 0.01	0.99	± 0.04	0.82	± 0.02	0.83	± 0.01	p<0.001	1.12	± 0.04	1.03	± 0.05		0.98	± 0.03	0.94	± 0.02
**Amygdala**																		
Basolateral Amygdala (BLA)	0.79	± 0.04	0.86	± 0.04	0.74	± 0.03	0.80	± 0.04		0.67	± 0.05	0.65	± 0.02	p<0.001	0.73	± 0.02	0.77	± 0.02
Medial Amygdala (MeA)	0.61	± 0.02	0.58	± 0.02	0.63	± 0.03	0.62	± 0.03		0.51	± 0.02	0.47	± 0.02	p<0.001	0.58	± 0.02	0.56	± 0.02
Central Amygdala (CeA)	0.70	± 0.04	0.62	± 0.04	0.79	± 0.03	0.69	± 0.04		0.62	± 0.03	0.58	± 0.04		0.70	± 0.02	0.63^{+}	± 0.02
**Dorsal Hippocampus (DH)**																		
Cornu Ammonis 1 (DHCA1)	0.77	± 0.02	0.76	± 0.03	0.77	± 0.02	0.79	± 0.03		0.80	± 0.04	0.78	± 0.03		0.78	± 0.02	0.78	± 0.02
Cornu Ammonis 2 (DHCA2)	0.82	± 0.02	0.82	± 0.03	0.74	± 0.03	0.74	± 0.03	p=0.008	0.78	± 0.02	0.77	± 0.03		0.78	± 0.01	0.77^{+}	± 0.02
Dentate Gyrus (DHDG)	0.72	± 0.02	0.70	± 0.03	0.79	± 0.02	0.82	± 0.03	p<0.001	0.68	± 0.02	0.65	± 0.03		0.73	± 0.02	0.73	± 0.02
Molecular Layer (DHML)	1.22	± 0.04	1.24	± 0.05	1.45	± 0.10	1.65	± 0.08	p<0.001	1.33	± 0.06	1.39	± 0.08	p=0.040	1.34	± 0.05	1.44	± 0.05
**Ventral Hippocampus (VH)**																		
Dorsal Subiculum (DS)	1.15	± 0.03	1.18	± 0.06	1.26	± 0.04	1.32	± 0.07	p=0.027	1.30	± 0.06	1.37	± 0.07	p=0.009	1.24	± 0.03	1.29	± 0.04
VH Cornu Ammonis 1 (VHCA1)	0.85	± 0.02	0.84	± 0.02	0.84	± 0.03	0.88	± 0.04		0.85	± 0.02	0.81	± 0.02		0.84	± 0.01	0.85	± 0.02
VH Cornu Ammonis 2 (VHCA2)	0.83	± 0.01	0.82	± 0.02	0.78	± 0.02	0.81	± 0.03		0.78	± 0.02	0.74	± 0.02	p=0.002	0.80	± 0.01	0.79	± 0.01
VH Cornu Ammonis 3 (VHCA3)	0.69	± 0.01	0.64	± 0.03	0.66	± 0.02	0.68	± 0.02		0.57	± 0.03	0.56	± 0.02	p<0.001	0.64	± 0.02	0.63	± 0.02
VH Dentate Gyrus (VHDG)	0.63	± 0.02	0.61	± 0.01	0.70	± 0.03	0.69	± 0.03	p<0.001	0.55	± 0.02	0.52	± 0.02	p<0.001	0.62	± 0.02	0.61	± 0.02
VH Molecular Layer (VHML)	1.06	± 0.02	1.05	± 0.02	1.11	± 0.03	1.16	± 0.04	p=0.003	0.93	± 0.03	0.90	± 0.02	p<0.001	1.03	± 0.02	1.05	± 0.03
**Raphé**																		
Dorsal Raphé (DR)	0.91	± 0.03	0.87	± 0.03	0.82	± 0.02	0.85	± 0.02		0.97	± 0.03	0.91	± 0.01		0.90	± 0.02	0.88	± 0.01
Median Raphé (MR)	1.17	± 0.03	1.19	± 0.06	0.99	± 0.02	1.06	± 0.03	p<0.001	1.15	± 0.03	1.20	± 0.05		1.10	± 0.02	1.15	± 0.03
**Multimodal**																		
Ventral Tegmental Nucleus (VTg)	1.33	± 0.06	1.34	± 0.05	1.10	± 0.02	1.19	± 0.04	p<0.001	1.53	± 0.08	1.58	± 0.08	p=0.002	1.32	± 0.05	1.36	± 0.04
Habenula (Hab)	1.20	± 0.02	1.28	± 0.07	1.11	± 0.05	1.10	± 0.04	p=0.006	0.98	± 0.07	0.90	± 0.06	p<0.001	1.09	± 0.03	1.08	± 0.04
Mamillary Body (MB)	2.01	± 0.09	1.85	± 0.09	1.83	± 0.08	2.01	± 0.08		2.07	± 0.08	2.58	± 0.21	p=0.011	1.95	± 0.05	2.12	± 0.09

Table S1. Alterations in Local Cerebral Glucose Utilisation (LCGU) induced by *Neurexin1 α* heterozygosity, 25mg/kg ketamine and 5mg/kg dextro-amphetamine administration. Data shown as Mean ± SEM. *p<0.05 significant effect of genotype within treatment group (pairwise t-test with Benjamini-Hochberg correction). $^{+}$denotes p<0.05 significant main effect of genotype (ANOVA all treatment groups; no significant sex x genotype or treatment x genotype interactions). $^{\#}$p<0.05 significant genotype x treatment interaction (ANOVA). $^{\$}$p<0.05, $^{\$\$}$p<0.01 and $^{\$\$\$}$p<0.001 significant treatment effect (within genotype, pairwise t-test with Benjamini-Hochberg correction). Significant main effects of drug treatment are also shown (ANOVA).
