# Supplementary material for: Ketamine Restores Thalamic-Prefrontal Cortex Functional Connectivity in a Mouse Model of Neurodevelopmental Disorder-Associated 2p16.3 Deletion
Source: Cereb Cortex. 2019 Dec 8;30(4):2358–71. doi: 10.1093/cercor/bhz244 (PMC7175007; doi:10.1093/cercor/bhz244)
Supplement: Table_S2_bhz244 [file table_s2_bhz244.pdf]

Region	Degree Centrality ($<k_i>$)			Betweenness Centrality (B_c)			Eigenvector Centrality (E_c)		
	Wild-type (WT)	Nrxn1α Hz	Difference	Wild-type (WT)	*Nrxn1 α* Hz	Difference	Wild-type (WT)	*Nrxn1 α* Hz	Difference
anterior Prelimbic Cortex (aPrL)	1.51	**-2.46**	**-3.97***	0.59	-1.37	-1.97	1.81	**-2.46**	**-4.27***
Frontal Association Area (FRA)	-1.29	-0.07	1.22	0.08	1.51	1.43	**-2.20**	**-2.36**	-0.17
Dorsolateral Orbital Cortex (DLO)	-0.46	-0.60	-0.13	-0.93	**3.15**	4.08	-0.42	**-2.35**	-1.94
Ventral Orbital Cortex (VO)	-1.20	-1.31	-0.11	0.51	-0.64	-1.15	**-2.10**	**-2.44**	-0.34
Medial Orbital Cortex (MO)	-1.55	-1.56	-0.01	-1.16	-1.30	-0.14	**-2.33**	**-2.16**	0.17
medial Prelimbic Cortex (mPrL)	1.10	-1.73	-2.82	**3.62**	-1.05	-4.67	0.18	-1.68	-1.86
Infralimbic Cortex (IL)	-0.60	**-1.99**	-1.39	-0.54	-1.38	-0.84	-1.44	-1.89	-0.44
Nucleus Accumbens Core (NaC)	-1.51	0.04	1.56	-0.43	0.01	0.44	**-2.22**	-0.27	1.96
Nucleus Accumbens Shell (NaS)	-0.06	1.78	1.84	-0.51	1.91	2.42	-1.80	1.17	**2.97***
Cingualte Cortex (Cg1)	1.09	-1.19	-2.27	**3.15**	-1.37	-4.52	-1.16	-0.94	0.22
Motor Cortex (M1)	0.39	-1.70	-2.09	**3.45**	0.80	-2.65	-1.74	**-2.43**	-0.69
Piriform Cortex (Piri)	0.74	0.04	-0.71	0.80	**2.38**	1.57	-1.55	-0.69	0.86
Insular Cortex (Ins)	-0.24	0.59	0.84	0.36	1.02	0.66	**-1.98**	-0.26	1.72
Ventromedial Striatum (VMST)	-0.49	**-2.35**	-1.86	-0.06	-1.39	-1.32	**-2.09**	**-2.17**	-0.08
Dorsolateral Striatum (DLST)	-0.31	0.37	0.68	0.87	1.72	0.85	**-1.99**	0.31	2.31
Medial Septum (MS)	-0.38	1.69	2.07	-0.91	-0.31	0.60	-1.94	**1.98**	**3.92***
Lateral Septum (LS)	-0.10	1.34	1.44	-0.98	0.93	1.91	-1.81	1.50	**3.31***
Ventral Limb of the Diagonal Band of Broca (VDB)	0.21	**3.04**	2.83	1.00	1.82	0.82	-1.64	**2.43**	**4.08***
Horizontal Limb of the Diagonal Band of Broca (HDB)	0.44	1.46	1.02	**1.97**	-0.55	-2.52	-1.91	1.90	**3.81***
Anteromedial Thalamus (AM)	-0.12	1.41	1.52	0.42	0.95	0.52	-0.15	1.82	1.97
Anteroventral Thalamus (AV)	0.23	1.30	1.06	-0.31	**2.28**	2.60	0.30	0.76	0.45
Somatosensory Cortex (SSCTX)	-1.36	-1.13	0.23	-0.40	0.72	1.12	-1.88	**-2.30**	-0.43
Globus Pallidus (GP)	-1.03	1.26	2.29	0.16	-0.06	-0.22	-1.51	1.58	**3.09***
Mediodoersal Thalamus (MD)	**2.41**	0.65	-1.76	**2.71**	1.88	-0.83	**2.35**	-1.32	**-3.67***
Centromedial Thalamus (CM)	1.48	-1.79	-3.27	0.15	0.09	-0.06	1.91	**-2.13**	**-4.04***
Centrolateral Thalamus (CL)	1.22	1.38	0.16	0.38	**3.16**	2.77	1.50	-1.68	-3.18
Ventrolateral Thalamus (VL)	0.29	1.21	0.92	**4.31**	**4.00**	-0.31	-0.49	-1.95	-1.46
Ventromedial Thalamus (VM)	0.75	0.68	-0.07	0.44	1.26	0.81	0.32	-1.92	-2.24
Nucleus Reuniens (Re)	0.99	0.30	-0.69	-0.68	0.11	0.79	1.51	**-2.18**	**-3.69***
dorsal Reticular Thalamus (dRT)	**2.45**	-0.25	-2.70	**2.25**	1.56	-0.69	**2.38**	-1.48	**-3.86***
ventral Reticular Thalamus (vRT)	0.82	-0.41	-1.23	-0.35	-1.31	-0.96	1.44	**-2.13**	**-3.57***
Basolateral Amygdala (BLA)	0.88	0.65	-0.23	1.38	-0.15	-1.53	0.65	**-2.08**	-2.73
Medial Amygdala (MeA)	-0.61	-0.18	0.43	-0.28	1.59	1.87	-1.49	-1.45	0.05
Central Amygdala (CeA)	-0.45	1.64	2.09	0.87	**3.20**	2.33	-1.15	1.74	**2.89***
Retrosplenial Cortex (RSC)	0.07	-1.20	-1.27	0.53	-0.01	-0.54	0.26	**-2.12**	-2.37
Habenula (Hab)	-1.71	0.53	2.24	-0.47	0.89	1.36	**-2.17**	**-2.04**	0.13
Cornu Ammonis 1 (DHCA1)	-1.39	0.23	1.62	-1.00	0.85	1.85	-1.23	-0.27	0.97
Cornu Ammonis 2 (DHCA2)	-1.05	1.33	2.38	-0.63	0.41	1.04	-1.85	1.52	**3.37***
Dentate Gyrus (DHDG)	0.88	-0.04	-0.92	-0.03	1.19	1.22	0.25	-0.55	-0.80
Molecular Layer (DHML)	1.12	-0.67	-1.79	**4.18**	1.22	-2.96	0.39	-1.20	-1.60
Auditory Cortex (AudC)	0.83	-0.41	-1.23	0.16	1.22	1.06	-1.54	**-2.38**	-0.84
Medial Geniculate (MG)	0.82	1.07	0.26	**2.08**	0.70	-1.38	-1.61	**-2.28**	-0.67
Dorsal Subiculum (DS)	0.71	0.20	-0.51	1.46	-0.47	-1.93	-1.38	**-2.29**	-0.90
VH Cornu Ammonis 1 (VHCA1)	-0.57	-1.02	-0.46	0.53	-0.93	-1.46	-1.59	**-2.34**	-0.75
VH Cornu Ammonis 2 (VHCA2)	**-2.31**	-0.29	2.02	-1.37	-0.44	0.92	**-2.28**	**-2.25**	0.03
VH Cornu Ammonis 3 (VHCA3)	0.57	-1.53	-2.10	**3.39**	0.04	-3.34	-0.98	**-2.29**	-1.31
VH Dentate Gyrus (VHDG)	0.92	-0.09	-1.01	**2.24**	1.75	-0.49	-0.72	-1.65	-0.92
VH Molecular Layer (VHML)	0.90	0.67	-0.23	0.32	0.51	0.20	-1.66	**-2.25**	-0.59
Mamillary Body (MB)	-0.90	0.39	1.29	-0.15	0.91	1.06	**-2.14**	**-2.29**	-0.15
Ventral Tegmental Area (VTA)	0.50	-0.07	-0.57	1.95	**2.66**	0.72	-1.31	**-2.24**	-0.93
Substantia Nigra pars Compacta (SNC)	-0.90	-0.66	0.24	**2.05**	0.83	-1.23	**-2.20**	**-2.39**	-0.19
Substantia Nigra pars Reticulata (SNR)	-0.87	1.54	2.41	0.02	**6.01**	**6.00***	-1.90	**-2.20**	-0.30
Dorsal Raphé (DR)	-1.92	**-2.47**	-0.55	-1.35	-1.39	-0.04	**-2.28**	**-2.47**	-0.19
Median Raphé (MR)	0.59	-0.25	-0.85	-0.29	**6.57**	**6.85***	-1.61	-1.33	0.28
Ventral Tegmental Nucleus (VTg)	0.75	0.34	-0.42	**2.41**	0.32	-2.09	-1.27	**-2.28**	-1.02
Perirhinal Cortex (PRh)	-0.14	0.38	0.52	-0.93	0.92	1.85	-1.78	**-2.36**	-0.58
anterior Reticular Thalamus (RT)	-1.45	0.61	2.06	-0.15	**4.66**	4.80	**-2.16**	-0.89	1.27
Entorhinal Cortex (EC)	-0.59	-0.73	-0.13	-0.92	**3.28**	4.20	-0.64	**-2.23**	-1.60

Table S2. Regional centrality alterations in functional brain networks of *Nrxn1 α* $^{+/-}$ mice. Data shown for region degree (k_i), betweenness (B_c) and eigenvector (E_c) centrality. Hub brain regions identified for each experimental group and centrality measure was determined by calculation of the standardised *z* -score from the measurement in the real brain networks relative to that in 11,000 calibrated Erdös-Rényi random networks. $z > 1.96$ identifies a significant regional hub in the brain networks, while $z < -1.96$ identifies regions that are significant exteriorities in the brain networks (bold). The difference in these *z* -scores between experimental groups is also shown. *denotes $p<0.05$ significant difference in regional centrality between the experimental groups (55,000 random permutations of the data).
