# Supplementary material for: Ketamine Restores Thalamic-Prefrontal Cortex Functional Connectivity in a Mouse Model of Neurodevelopmental Disorder-Associated 2p16.3 Deletion
Source: Cereb Cortex. 2019 Dec 8;30(4):2358–71. doi: 10.1093/cercor/bhz244 (PMC7175007; doi:10.1093/cercor/bhz244)
Supplement: Tables_S3_bhz244 [file tables_s3_bhz244.pdf]

Table S3. PLSR defined connectivity of the anterior Prelimbic cortex (aPrL) in saline-treated wild-type (WT) mice and *Nrxn1α*$^{+/-}$ mice.

Genotype	Wild-type (WT)			*Neurexin1 α* $^{+/-}$		
**Treatment**	**Saline**			**Saline**		
**Region**	**Mean ± SE**			**Mean ± SE**		
Frontal Association Area (FRA)	0.85	±	0.10	**1.16**	**±**	**0.04**
Dorsolateral Orbital Cortex (DLO)	0.68	±	0.08	**1.18**	**±**	**0.05**
Ventral Orbital Cortex (VO)	0.75	±	0.06	0.77	±	0.03
Medial Orbital Cortex (MO)	0.80	±	0.07	0.66	±	0.05
medial Prelimbic Cortex (mPrL)	**1.45**	**±**	**0.04**	0.77***	±	0.04
Infralimbic Cortex (IL)	0.48	±	0.05	0.64	±	0.04
Nucleus Accumbens Core (NaC)	0.76	±	0.12	0.66	±	0.06
Nucleus Accumbens Shell (NaS)	0.63	±	0.07	0.58	±	0.06
Cingualte Cortex (Cg1)	0.70	±	0.07	**1.64**	**±**	**0.08**
Motor Cortex (M1)	0.86	±	0.09	**1.83**	**±**	**0.09**
Piriform Cortex (Piri)	0.98	±	0.08	0.60	±	0.06
Insular Cortex (Ins)	0.53	±	0.09	0.92	±	0.06
Ventromedial Striatum (VMST)	0.48	±	0.11	**1.19**	**±**	**0.10**
Dorsolateral Striatum (DLST)	0.84	±	0.06	**1.22**	**±**	**0.05**
Medial Septum (MS)	1.03	±	0.10	0.89	±	0.07
Lateral Septum (LS)	0.76	±	0.07	**1.18**	**±**	**0.06**
Ventral Limb of the Diagonal Band of Broca (VDB)	0.44	±	0.06	0.71	±	0.06
Horizontal Limb of the Diagonal Band of Broca (HDB)	0.60	±	0.03	**1.38**	**±**	**0.03**
Anteromedial Thalamus (AM)	**1.30**	**±**	**0.07**	**1.19**	**±**	**0.05**
Anteroventral Thalamus (AV)	0.69	±	0.08	**1.20**	**±**	**0.04**
Somatosensory Cortex (SSCTX)	0.44	±	0.12	0.94	±	0.10
Globus Pallidus (GP)	0.84	±	0.08	0.67	±	0.07
Mediodoersal Thalamus (MD)	**1.35**	**±**	**0.09**	0.77*	±	0.08
Centromedial Thalamus (CM)	**1.73**	**±**	**0.06**	0.83***	±	0.07
Centrolateral Thalamus (CL)	**1.38**	**±**	**0.07**	0.56***	±	0.05
Ventrolateral Thalamus (VL)	0.71	±	0.09	0.49	±	0.07
Ventromedial Thalamus (VM)	0.55	±	0.09	0.52	±	0.05
Nucleus Reuniens (Re)	**1.95**	**±**	**0.05**	**1.05**	**±**	**0.07**
dorsal Reticular Thalamus (dRT)	**1.88**	**±**	**0.04**	0.90***	±	0.05
ventral Reticular Thalamus (vRT)	**1.31**	**±**	**0.12**	0.67*	±	0.06
Basolateral Amygdala (BLA)	**1.75**	**±**	**0.05**	0.73***	±	0.09
Medial Amygdala (MeA)	0.83	±	0.10	**1.16**	**±**	**0.09**
Central Amygdala (CeA)	0.84	±	0.10	**1.47**	**±**	**0.04**
Retrosplenial Cortex (RSC)	0.87	±	0.11	0.59	±	0.07
Habenula (Hab)	0.71	±	0.07	0.86	±	0.07
Cornu Ammonis 1 (DHCA1)	0.99	±	0.06	0.73	±	0.05
Cornu Ammonis 2 (DHCA2)	0.57	±	0.05	0.82	±	0.05
Dentate Gyrus (DHDG)	**1.24**	**±**	**0.06**	0.81	±	0.07
Molecular Layer (DHML)	0.49	±	0.13	0.92	±	0.09
Auditory Cortex (AudC)	0.87	±	0.08	0.92	±	0.05
Medial Geniculate (MG)	0.71	±	0.05	0.69	±	0.04
Dorsal Subiculum (DS)	0.51	±	0.07	0.81	±	0.05
VH Cornu Ammonis 1 (VHCA1)	0.61	±	0.10	0.39	±	0.05
VH Cornu Ammonis 2 (VHCA2)	0.70	±	0.16	0.55	±	0.07
VH Cornu Ammonis 3 (VHCA3)	0.53	±	0.07	0.80	±	0.08
VH Dentate Gyrus (VHDG)	0.96	±	0.06	**1.16**	**±**	**0.05**
VH Molecular Layer (VHML)	0.75	±	0.09	**1.03**	**±**	**0.04**
Mamillary Body (MB)	0.47	±	0.06	**1.12**	**±**	**0.03**
Ventral Tegmental Area (VTA)	0.92	±	0.09	0.79	±	0.07
Substantia Nigra pars Compacta (SNC)	0.49	±	0.08	**1.77**	**±**	**0.08**
Substantia Nigra pars Reticulata (SNR)	0.61	±	0.04	0.62	±	0.11
Dorsal Raphé (DR)	0.68	±	0.09	1.00	±	0.09
Median Raphé (MR)	**1.59**	**±**	**0.06**	**1.18**	**±**	**0.08**
Ventral Tegmental Nucleus (VTg)	**1.40**	**±**	**0.10**	**1.18**	**±**	**0.04**
Perirhinal Cortex (PRh)	1.04	±	0.12	0.83	±	0.05
anterior Reticular Thalamus (RT)	0.46	±	0.08	**1.37**	**±**	**0.07**
Entorhinal Cortex (EC)	**1.42**	**±**	**0.07**	0.97*	±	0.04

*Table S3. Data shown as the Mean ± SEM variable importance to the projection (VIP) statistic. VIPs with a 95% confidence interval (CI) >1.0 are considered to be connected to the aPrL ("seed") region (bold). *denotes p<0.05 and ***denotes p<0.001 significant difference from saline-treated WT mice (t-test with Bonferroni correction).*

Table S4. PLSR defined connectivity of the dorsal reticular thalamus (dRT) in saline-treated wild-type (WT) and *Nrxn1α*$^{+/-}$ mice and ketamine-treated *Nrxn1α*$^{+/-}$ mice.

**Genotype**	**Wild-type**			***Neurexin1 α*** $^{+/-}$			***Neurexin1 α*** $^{+/-}$		
**Treatment**	**Saline**			**Saline**			**Ketamine**		
**Region**	**Mean**	**±**	**SE**	**Mean**	**±**	**SE**	**Mean**	**±**	**SE**
anterior Prelimbic Cortex (aPrL)	**1.86**	±	**0.03**	0.79***	±	0.05	0.70**	±	0.04
Frontal Association Area (FRA)	0.82	±	0.11	**1.15**	±	**0.07**	0.70^{+}	±	0.05
Dorsolateral Orbital Cortex (DLO)	**1.05**	±	**0.09**	0.94	±	0.06	0.40***	±	0.03
Ventral Orbital Cortex (VO)	0.62	±	0.07	**1.32**	±	**0.08**	**1.21*** **	±	**0.03**
Medial Orbital Cortex (MO)	0.89	±	0.09	0.84	±	0.05	0.84	±	0.04
medial Prelimbic Cortex (mPrL)	**1.68**	±	**0.04**	**1.13**	±	**0.03**	0.71^{+++}***	±	0.03
Infralimbic Cortex (IL)	0.75	±	0.07	**1.43**	±	**0.05**	0.45^{+++}	±	0.04
Nucleus Accumbens Core (NaC)	0.50	±	0.04	0.67	±	0.04	0.88	±	0.02
Nucleus Accumbens Shell (NaS)	0.36	±	0.07	0.72	±	0.03	0.74	±	0.04
Cingualte Cortex (Cg1)	0.86	±	0.14	0.87	±	0.05	0.35	±	0.03
Motor Cortex (M1)	0.62	±	0.09	0.60	±	0.04	0.94	±	0.05
Piriform Cortex (Piri)	0.47	±	0.05	**1.30**	±	**0.09**	**1.94*** **	±	**0.02**
Insular Cortex (Ins)	0.35	±	0.07	0.64	±	0.05	**1.29^{+++}*** **	±	**0.02**
Ventromedial Striatum (VMST)	0.43	±	0.07	0.91	±	0.06	0.45	±	0.03
Dorsolateral Striatum (DLST)	0.78	±	0.09	0.81	±	0.04	0.49	±	0.03
Medial Septum (MS)	0.67	±	0.09	0.86	±	0.03	0.87	±	0.04
Lateral Septum (LS)	0.66	±	0.05	0.71	±	0.03	0.53	±	0.05
Ventral Limb of the Diagonal Band of Broca (VDB)	0.41	±	0.04	**1.08**	±	**0.05**	**1.19*** **	±	**0.04**
Horizontal Limb of the Diagonal Band of Broca (HDB)	0.60	±	0.06	0.85	±	0.04	0.59	±	0.02
Anteromedial Thalamus (AM)	**1.41**	±	**0.11**	0.78***	±	0.04	0.91*	±	0.04
Anteroventral Thalamus (AV)	**1.54**	±	**0.05**	0.96**	±	0.08	0.46***	±	0.04
Somatosensory Cortex (SSCTX)	0.50	±	0.09	0.88	±	0.05	0.49	±	0.04
Globus Pallidus (GP)	0.41	±	0.05	**1.15**	±	**0.07**	**1.44*** **	±	**0.04**
Mediodoersal Thalamus (MD)	**1.79**	±	**0.05**	**1.51**	±	**0.07**	**1.07**	±	**0.04**
Centromedial Thalamus (CM)	**1.95**	±	**0.03**	**1.51**	±	**0.05**	**1.93**	±	**0.02**
Centrolateral Thalamus (CL)	**1.38**	±	**0.09**	**1.40**	±	**0.06**	**1.07**	±	**0.03**
Ventrolateral Thalamus (VL)	0.51	±	0.08	**1.06**	±	**0.05**	**1.13****	±	**0.05**
Ventromedial Thalamus (VM)	0.95	±	0.13	**1.23**	±	**0.04**	**1.33**	±	**0.03**
Nucleus Reuniens (Re)	**1.80**	±	**0.04**	0.57***	±	0.04	**1.18^{+++}**	±	**0.02**
ventral Reticular Thalamus (vRT)	**1.66**	±	**0.04**	**1.14**	±	**0.08**	**1.75**	±	**0.02**
Basolateral Amygdala (BLA)	**1.45**	±	**0.06**	0.88**	±	0.07	0.42***	±	0.03
Medial Amygdala (MeA)	0.59	±	0.09	0.51	±	0.05	**1.27*** **	±	**0.02**
Central Amygdala (CeA)	0.67	±	0.09	**1.47**	±	**0.05**	0.96^{+++}	±	0.03
Retrosplenial Cortex (RSC)	**1.34**	±	**0.09**	**1.86**	±	**0.09**	0.57^{+++}	±	0.03
Habenula (Hab)	0.35	±	0.05	0.81	±	0.05	**1.72^{+++}**	±	**0.05**
Cornu Ammonis 1 (DHCA1)	**1.22**	±	**0.12**	**1.09**	±	**0.08**	**1.63**	±	**0.02**
Cornu Ammonis 2 (DHCA2)	0.55	±	0.05	**1.08**	±	**0.08**	**1.07*** **	±	**0.03**
Dentate Gyrus (DHDG)	1.01	±	0.13	0.86	±	0.04	**1.04**	±	**0.03**
Molecular Layer (DHML)	**1.19**	±	**0.08**	**1.10**	±	**0.03**	**1.07**	±	**0.03**
Auditory Cortex (AudC)	0.78	±	0.06	1.02	±	0.05	**1.12**	±	**0.06**
Medial Geniculate (MG)	0.48	±	0.08	0.89	±	0.06	0.93	±	0.04
Dorsal Subiculum (DS)	0.44	±	0.12	1.00	±	0.04	0.68	±	0.04
VH Cornu Ammonis 1 (VHCA1)	0.40	±	0.09	0.81	±	0.10	0.69	±	0.03
VH Cornu Ammonis 2 (VHCA2)	0.69	±	0.10	0.59	±	0.08	0.35	±	0.04
VH Cornu Ammonis 3 (VHCA3)	0.95	±	0.12	0.63	±	0.06	0.73	±	0.04
VH Dentate Gyrus (VHDG)	**1.22**	±	**0.07**	0.59***	±	0.05	0.66***	±	0.03
VH Molecular Layer (VHML)	0.59	±	0.09	0.83	±	0.07	**1.35^{+++}*** **	±	**0.02**
Mamillary Body (MB)	0.43	±	0.06	0.67	±	0.08	0.96	±	0.05
Ventral Tegmental Area (VTA)	0.48	±	0.06	0.60	±	0.10	0.49	±	0.04
Substantia Nigra pars Compacta (SNC)	0.88	±	0.06	0.50	±	0.05	0.71	±	0.03
Substantia Nigra pars Reticulata (SNR)	0.42	±	0.05	0.81	±	0.03	0.74	±	0.03
Dorsal Raphé (DR)	**1.09**	±	**0.11**	0.50	±	0.06	0.70	±	0.04
Median Raphé (MR)	**1.36**	±	**0.06**	0.63***	±	0.04	0.86***	±	0.03
Ventral Tegmental Nucleus (VTg)	0.68	±	0.06	0.63	±	0.06	0.72	±	0.03
Perirhinal Cortex (PRh)	0.74	±	0.09	**1.04**	±	**0.05**	0.97	±	0.04
anterior Reticular Thalamus (RT)	0.35	±	0.07	**1.39**	±	**0.05**	0.70^{+++}	±	0.04
Entorhinal Cortex (EC)	0.67	±	0.07	0.86	±	0.07	0.88	±	0.00

*Table S4. Data shown as the Mean ± SEM variable importance to the projection (VIP) statistic. VIPs with a 95% confidence interval (CI) >1.0 are considered to be connected to the dRT ("seed") region (bold). *p<0.05, **p<0.01 and ***p<0.001 significant difference from saline-treated WT mice (t-test with Bonferroni correction).* $^{+}$*p<0.05,* $^{++}$*p<0.01 and* $^{+++}$*p<0.001 significant difference from saline-treated Nrxn1α*$^{+/-}$ *mice (t-test with Bonferroni correction).*

Table S5. PLSR defined connectivity of the ventral reticular thalamus (vRT) in saline-treated wild-type (WT) and *Nrxn1α$^{+/-}$* mice and ketamine-treated *Nrxn1α$^{+/-}$* mice

**Genotype**	**Wild-type**			***Neurexin1 α*** $^{+/-}$			***Neurexin1 α*** $^{+/-}$		
**Treatment**	**Saline**			**Saline**			**Ketamine**		
**Region**	**Mean ± SE**			**Mean ± SE**			**Mean ± SE**		
anterior Prelimbic Cortex (aPrL)	**1.33**	±	**0.11**	0.59**	±	0.05	0.55**	±	0.03
Frontal Association Area (FRA)	0.48	±	0.12	0.67	±	0.03	0.65	±	0.04
Dorsolateral Orbital Cortex (DLO)	**1.26**	±	**0.07**	0.55***	±	0.07	0.69**	±	0.06
Ventral Orbital Cortex (VO)	0.62	±	0.10	0.56	±	0.05	0.66	±	0.04
Medial Orbital Cortex (MO)	0.74	±	0.12	**1.04**	±	**0.04**	**1.03**	±	**0.02**
medial Prelimbic Cortex (mPrL)	0.68	±	0.06	**1.78**	±	**0.03**	0.56^{+++}	±	0.02
Infralimbic Cortex (IL)	0.35	±	0.06	**1.81**	±	**0.03**	0.75^{+++}***	±	0.03
Nucleus Accumbens Core (NaC)	0.94	±	0.14	**1.40**	±	**0.04**	0.56^{+++}	±	0.03
Nucleus Accumbens Shell (NaS)	0.93	±	0.09	**1.53**	±	**0.03**	0.62^{+++}	±	0.04
Cingualte Cortex (Cg1)	0.45	±	0.06	0.82	±	0.08	0.68	±	0.03
Motor Cortex (M1)	0.63	±	0.08	0.59	±	0.03	0.68	±	0.03
Piriform Cortex (Piri)	0.68	±	0.09	0.59	±	0.04	**1.26^{+++}*** **	±	**0.03**
Insular Cortex (Ins)	**1.11**	±	**0.08**	0.91	±	0.05	**1.54^{+++}**	±	**0.02**
Ventromedial Striatum (VMST)	0.48	±	0.08	0.54	±	0.07	0.59	±	0.03
Dorsolateral Striatum (DLST)	0.56	±	0.08	0.59	±	0.05	0.80	±	0.02
Medial Septum (MS)	0.34	±	0.11	**1.06**	±	**0.05**	**1.04*** **	±	**0.02**
Lateral Septum (LS)	0.37	±	0.06	**1.24**	±	**0.04**	0.47^{+++}	±	0.04
Ventral Limb of the Diagonal Band of Broca (VDB)	**1.10**	±	**0.07**	0.89	±	0.05	**1.08**	±	**0.03**
Horizontal Limb of the Diagonal Band of Broca (HDB)	0.94	±	0.10	**1.14**	±	**0.04**	0.73^{+++}	±	0.02
Anteromedial Thalamus (AM)	0.60	±	0.11	0.73	±	0.04	**1.75^{+++}**	±	**0.02**
Anteroventral Thalamus (AV)	**1.09**	±	**0.09**	0.56	±	0.07	0.60*	±	0.03
Somatosensory Cortex (SSCTX)	0.35	±	0.10	0.94	±	0.06	0.67	±	0.04
Globus Pallidus (GP)	0.32	±	0.06	0.84	±	0.04	0.89	±	0.04
Mediodoersal Thalamus (MD)	**1.86**	±	**0.05**	**1.05**	±	**0.06**	**1.57**	±	**0.02**
Centromedial Thalamus (CM)	**1.91**	±	**0.03**	0.85***	±	0.05	**1.39^{+++}**	±	**0.03**
Centrolateral Thalamus (CL)	**1.65**	±	**0.04**	**1.39**	±	**0.04**	**1.31**	±	**0.03**
Ventrolateral Thalamus (VL)	**1.20**	±	**0.05**	**1.68**	±	**0.03**	**1.19**	±	**0.04**
Ventromedial Thalamus (VM)	**1.39**	±	**0.06**	**1.45**	±	**0.03**	**1.21**	±	**0.02**
Nucleus Reuniens (Re)	**1.80**	±	**0.05**	**1.54**	±	**0.04**	0.63^{+++}***	±	0.03
dorsal Reticular Thalamus (dRT)	**1.71**	±	**0.04**	**1.07**	±	**0.09**	**1.74**	±	**0.03**
Basolateral Amygdala (BLA)	**1.06**	±	**0.06**	**1.35**	±	**0.06**	0.73^{+++}	±	0.04
Medial Amygdala (MeA)	0.44	±	0.06	0.96	±	0.03	0.79	±	0.02
Central Amygdala (CeA)	0.86	±	0.11	0.60	±	0.05	0.94	±	0.03
Retrosplenial Cortex (RSC)	**1.10**	±	**0.12**	1.04	±	0.08	0.97	±	0.04
Habenula (Hab)	0.89	±	0.11	**1.30**	±	**0.07**	**1.49** **	±	**0.03**
Cornu Ammonis 1 (DHCA1)	0.80	±	0.13	0.71	±	0.02	**1.42^{+++}** **	±	**0.02**
Cornu Ammonis 2 (DHCA2)	0.70	±	0.08	0.73	±	0.03	**1.04^{+++}** **	±	**0.02**
Dentate Gyrus (DHDG)	0.83	±	0.09	0.71	±	0.04	**1.23^{+++}**	±	**0.02**
Molecular Layer (DHML)	**1.55**	±	**0.05**	1.00	±	0.05	0.81***	±	0.03
Auditory Cortex (AudC)	0.53	±	0.05	0.63	±	0.03	0.87	±	0.04
Medial Geniculate (MG)	0.70	±	0.13	0.80	±	0.05	0.65	±	0.03
Dorsal Subiculum (DS)	0.43	±	0.10	0.98	±	0.02	0.97	±	0.05
VH Cornu Ammonis 1 (VHCA1)	0.98	±	0.12	0.92	±	0.06	0.60	±	0.04
VH Cornu Ammonis 2 (VHCA2)	**1.71**	±	**0.08**	**1.04**	±	**0.04**	0.90	±	0.04
VH Cornu Ammonis 3 (VHCA3)	0.79	±	0.09	0.62	±	0.09	0.49	±	0.03
VH Dentate Gyrus (VHDG)	0.60	±	0.09	0.74	±	0.06	0.45	±	0.03
VH Molecular Layer (VHML)	0.57	±	0.08	0.90	±	0.03	**1.59^{+++}**	±	**0.01**
Mamillary Body (MB)	0.99	±	0.08	0.59	±	0.04	0.49	±	0.03
Ventral Tegmental Area (VTA)	1.01	±	0.11	0.64	±	0.03	0.48	±	0.06
Substantia Nigra pars Compacta (SNC)	**1.25**	±	**0.07**	0.62***	±	0.03	**1.18^{+++}**	±	**0.05**
Substantia Nigra pars Reticulata (SNR)	0.58	±	0.09	0.94	±	0.04	1.01	±	0.03
Dorsal Raphé (DR)	0.48	±	0.05	0.66	±	0.07	**1.28^{+++}*** **	±	**0.03**
Median Raphé (MR)	0.84	±	0.08	0.88	±	0.06	0.82	±	0.04
Ventral Tegmental Nucleus (VTg)	0.46	±	0.05	0.90	±	0.05	0.46	±	0.02
Perirhinal Cortex (PRh)	0.41	±	0.06	0.70	±	0.04	**1.29^{+++}*** **	±	**0.03**
anterior Reticular Thalamus (RT)	0.76	±	0.09	0.99	±	0.05	0.40	±	0.04
Entorhinal Cortex (EC)	0.51	±	0.05	0.79	±	0.06	**1.41^{+++}*** **	±	**0.00**

*Table S5. Data shown as the Mean ± SEM variable importance to the projection (VIP) statistic. VIPs with a 95% confidence interval (CI) >1.0 are considered to be connected to the vRT ("seed") region (bold). *p<0.05, **p<0.01 and ***p<0.001 significant difference from saline-treated WT mice (t-test with Bonferroni correction). $^{+}$p<0.05, $^{++}$p<0.01 and $^{+++}$p<0.001 significant difference from saline-treated Nrxn1α$^{+/-}$ mice (t-test with Bonferroni correction).*

Table S6. PLSR defined connectivity of the mediodorsal thalamus (MD) in saline-treated wild-type (WT) and *Nrxn1α*$^{+/-}$ mice and ketamine-treated *Nrxn1α*$^{+/-}$ mice

Genotype	Wild-type (WT)	Neurexin1α Hz	Neurexin1α Hz
Treatment	Saline	Saline	Ketamine
Region	Mean ± SE	Mean ± SE	Mean ± SE
anterior Prelimbic Cortex (aPrL)	**1.40** ± **0.08**	0.50*** ± 0.06	0.70*** ± 0.03
Frontal Association Area (FRA)	0.33 ± 0.08	0.97 ± 0.08	0.91 ± 0.03
Dorsolateral Orbital Cortex (DLO)	**1.12** ± **0.09**	**1.40** ± **0.06**	0.50**$^{+++}$ ± 0.05
Ventral Orbital Cortex (VO)	0.44 ± 0.06	**1.38** ± **0.07**	0.52^{+++} ± 0.04
Medial Orbital Cortex (MO)	0.57 ± 0.06	**1.66** ± **0.05**	0.88^{+++} ± 0.03
medial Prelimbic Cortex (mPrL)	0.70 ± 0.06	**1.01** ± **0.09**	0.93 ± 0.05
Infralimbic Cortex (IL)	0.46 ± 0.07	**1.38** ± **0.07**	0.75^{+++} ± 0.03
Nucleus Accumbens Core (NaC)	0.32 ± 0.06	0.64 ± 0.07	0.42 ± 0.03
Nucleus Accumbens Shell (NaS)	0.31 ± 0.04	0.51 ± 0.10	0.58 ± 0.03
Cingualte Cortex (Cg1)	0.47 ± 0.09	0.43 ± 0.05	0.89 ± 0.03
Motor Cortex (M1)	0.79 ± 0.09	0.43 ± 0.05	**1.07**$^{+++}$ ± **0.02**
Piriform Cortex (Piri)	0.31 ± 0.08	**1.15** ± **0.10**	0.90 ± 0.04
Insular Cortex (Ins)	0.57 ± 0.07	0.83 ± 0.08	**1.66*****$^{+++}$ ± **0.02**
Ventromedial Striatum (VMST)	0.90 ± 0.12	0.53 ± 0.04	0.89 ± 0.03
Dorsolateral Striatum (DLST)	0.53 ± 0.10	0.91 ± 0.07	0.92 ± 0.03
Medial Septum (MS)	0.51 ± 0.05	0.41 ± 0.07	**1.04****$^{+++}$ ± **0.05**
Lateral Septum (LS)	0.49 ± 0.05	0.60 ± 0.05	1.00 ± 0.08
Ventral Limb of the Diagonal Band of Broca (VDB)	0.52 ± 0.10	0.62 ± 0.07	0.84 ± 0.03
Horizontal Limb of the Diagonal Band of Broca (HDB)	0.42 ± 0.09	0.58 ± 0.08	0.96 ± 0.05
Anteromedial Thalamus (AM)	**1.56** ± **0.08**	0.61*** ± 0.05	**1.50**$^{+++}$ ± **0.03**
Anteroventral Thalamus (AV)	**1.72** ± **0.06**	**1.33** ± **0.07**	0.75***$^{+++}$ ± 0.04
Somatosensory Cortex (SSCTX)	**1.20** ± **0.05**	0.76* ± 0.07	0.62*** ± 0.06
Globus Pallidus (GP)	0.44 ± 0.05	0.48 ± 0.10	0.83 ± 0.04
Centromedial Thalamus (CM)	**1.63** ± **0.09**	1.00* ± 0.07	**1.09**** ± **0.05**
Centrolateral Thalamus (CL)	**1.78** ± **0.06**	**1.90** ± **0.04**	**1.69** ± **0.03**
Ventrolateral Thalamus (VL)	**1.41** ± **0.05**	**1.09** ± **0.05**	**1.69***$^{+++}$ ± **0.03**
Ventromedial Thalamus (VM)	**1.74** ± **0.07**	**1.22** ± **0.07**	**1.49** ± **0.04**
Nucleus Reuniens (Re)	**1.53** ± **0.06**	0.48* ± 0.06	0.55*** ± 0.03
dorsal Reticular Thalamus (dRT)	**1.87** ± **0.05**	**1.42** ± **0.08**	**1.11***** ± **0.04**
ventral Reticular Thalamus (vRT)	**1.89** ± **0.04**	**1.07** ± **0.08**	**1.61****$^{+++}$ ± **0.02**
Basolateral Amygdala (BLA)	0.47 ± 0.06	0.74 ± 0.06	0.71 ± 0.03
Medial Amygdala (MeA)	0.54 ± 0.12	**1.27** ± **0.03**	0.66^{+++} ± 0.03
Central Amygdala (CeA)	0.79 ± 0.09	0.95 ± 0.08	0.59 ± 0.03
Retrosplenial Cortex (RSC)	**1.63** ± **0.07**	**1.79** ± **0.04**	0.66***$^{+++}$ ± 0.03
Habenula (Hab)	0.44 ± 0.14	**1.30** ± **0.03**	0.92^{+++} ± 0.03
Cornu Ammonis 1 (DHCA1)	1.03 ± 0.14	0.45 ± 0.07	0.81 ± 0.04
Cornu Ammonis 2 (DHCA2)	0.40 ± 0.03	0.89 ± 0.06	0.78 ± 0.04
Dentate Gyrus (DHDG)	0.75 ± 0.05	0.65 ± 0.03	0.78 ± 0.03
Molecular Layer (DHML)	**1.67** ± **0.05**	**1.68** ± **0.07**	0.60***$^{+++}$ ± 0.02
Auditory Cortex (AudC)	0.79 ± 0.07	0.78 ± 0.04	0.76 ± 0.04
Medial Geniculate (MG)	0.63 ± 0.09	0.45 ± 0.13	0.66 ± 0.03
Dorsal Subiculum (DS)	0.38 ± 0.10	0.82 ± 0.07	0.86 ± 0.02
VH Cornu Ammonis 1 (VHCA1)	**1.10** ± **0.09**	0.70 ± 0.06	0.84 ± 0.03
VH Cornu Ammonis 2 (VHCA2)	**1.47** ± **0.09**	0.42* ± 0.05	**1.30**$^{+++}$ ± **0.04**
VH Cornu Ammonis 3 (VHCA3)	0.22 ± 0.05	0.83 ± 0.08	0.59 ± 0.04
VH Dentate Gyrus (VHDG)	0.38 ± 0.07	0.77 ± 0.05	0.58 ± 0.04
VH Molecular Layer (VHML)	0.52 ± 0.10	0.83 ± 0.09	**1.38*****$^{+++}$ ± **0.05**
Mamillary Body (MB)	**1.15** ± **0.09**	0.76 ± 0.11	0.78 ± 0.06
Ventral Tegmental Area (VTA)	0.57 ± 0.13	0.70 ± 0.09	0.88 ± 0.11
Substantia Nigra pars Compacta (SNC)	0.38 ± 0.05	0.55 ± 0.06	**1.20*****$^{+++}$ ± **0.06**
Substantia Nigra pars Reticulata (SNR)	0.62 ± 0.06	0.40 ± 0.08	**1.01*****$^{+++}$ ± **0.02**
Dorsal Raphé (DR)	0.68 ± 0.09	0.46 ± 0.06	**1.41*****$^{+++}$ ± **0.04**
Median Raphé (MR)	**1.22** ± **0.06**	**1.35** ± **0.06**	0.69*** ± 0.03
Ventral Tegmental Nucleus (VTg)	0.37 ± 0.08	0.64 ± 0.08	0.49 ± 0.03
Perirhinal Cortex (PRh)	0.79 ± 0.05	0.71 ± 0.06	**1.31****$^{++}$ ± **0.07**
anterior Reticular Thalamus (RT)	0.47 ± 0.06	**1.63** ± **0.04**	0.95^{+++} ± 0.07
Entorhinal Cortex (EC)	0.36 ± 0.05	**1.14** ± **0.08**	**1.58***** ± **0.00**

*Table S6. Data shown as the Mean ± SEM variable importance to the projection (VIP) statistic. VIPs with a 95% confidence interval (CI) >1.0 are considered to be connected to the MD ("seed") region (bold). *p<0.05, **p<0.01 and ***p<0.001 significant difference from saline-treated WT mice (t-test with Bonferroni correction). $^{+}$p<0.05, $^{++}$p<0.01 and $^{+++}$p<0.001 significant difference from saline-treated Nrxn1α$^{+/-}$ mice (t-test with Bonferroni correction).*

Table S7. PLSR defined connectivity of the nucleus reuniens (Re) in saline-treated wild-type (WT) and *Nrxn1α*$^{+/-}$ mice and ketamine-treated *Nrxn1α*$^{+/-}$ mice

Genotype	Wild-type (WT)			Neurexin1α Hz			Neurexin1α Hz		
**Treatment**	**Saline**			**Saline**			**Ketamine**		
**Region**	**Mean ± SE**			**Mean ± SE**			**Mean ± SE**		
anterior Prelimbic Cortex (aPrL)	**1.90**	±	**0.05**	0.90***	±	0.09	**1.73^{+++}**	±	**0.03**
Frontal Association Area (FRA)	0.78	±	0.13	1.03	±	0.06	**1.13**	±	**0.03**
Dorsolateral Orbital Cortex (DLO)	0.85	±	0.11	0.86	±	0.08	0.78	±	0.03
Ventral Orbital Cortex (VO)	**1.20**	±	**0.09**	0.60**	±	0.04	**1.20^{+++}**	±	**0.03**
Medial Orbital Cortex (MO)	1.00	±	0.10	0.64	±	0.05	0.53	±	0.03
medial Prelimbic Cortex (mPrL)	**1.01**	±	**0.09**	**1.27**	±	**0.07**	0.89^{+}	±	0.04
Infralimbic Cortex (IL)	0.42	±	0.04	**1.29**	±	**0.05**	0.87^{+++}	±	0.03
Nucleus Accumbens Core (NaC)	0.65	±	0.07	**1.11**	±	**0.04**	**1.15*** **	±	**0.03**
Nucleus Accumbens Shell (NaS)	0.37	±	0.09	**1.29**	±	**0.03**	**1.06*** **	±	**0.03**
Cingualte Cortex (Cg1)	0.45	±	0.06	0.70	±	0.05	0.71	±	0.02
Motor Cortex (M1)	0.73	±	0.07	**1.07**	±	**0.07**	**1.57*** **	±	**0.02**
Piriform Cortex (Piri)	0.66	±	0.09	0.53	±	0.04	1.00	±	0.03
Insular Cortex (Ins)	0.57	±	0.08	0.83	±	0.04	1.01	±	0.04
Ventromedial Striatum (VMST)	0.53	±	0.11	**1.06**	±	**0.06**	0.66^{++}	±	0.03
Dorsolateral Striatum (DLST)	1.00	±	0.07	0.66	±	0.05	0.81	±	0.04
Medial Septum (MS)	0.52	±	0.04	**1.33**	±	**0.04**	**1.02*** **	±	**0.02**
Lateral Septum (LS)	0.57	±	0.05	**1.42**	±	**0.03**	0.97^{+++}	±	0.04
Ventral Limb of the Diagonal Band of Broca (VDB)	0.76	±	0.08	**1.08**	±	**0.03**	0.49^{+++}	±	0.04
Horizontal Limb of the Diagonal Band of Broca (HDB)	0.91	±	0.08	**1.47**	±	**0.04**	0.22^{+++}	±	0.03
Anteromedial Thalamus (AM)	0.75	±	0.12	**1.07**	±	**0.04**	0.35^{+++}	±	0.04
Anteroventral Thalamus (AV)	0.56	±	0.11	0.74	±	0.06	**1.04**	±	**0.05**
Somatosensory Cortex (SSCTX)	0.38	±	0.11	0.71	±	0.05	0.90	±	0.03
Globus Pallidus (GP)	0.83	±	0.08	0.88	±	0.04	0.76	±	0.02
Mediodorsal Thalamus (MD)	**1.43**	±	**0.06**	0.53***	±	0.05	0.45***	±	0.03
Centromedial Thalamus (CM)	**1.83**	±	**0.04**	0.79***	±	0.05	**1.45^{+++}**	±	**0.01**
Centrolateral Thalamus (CL)	**1.77**	±	**0.05**	0.97***	±	0.05	0.72***	±	0.04
Ventrolateral Thalamus (VL)	0.37	±	0.07	**1.58**	±	**0.04**	0.44^{+++}	±	0.06
Ventromedial Thalamus (VM)	0.64	±	0.06	**1.41**	±	**0.07**	0.99	±	0.06
dorsal Reticular Thalamus (dRT)	**1.75**	±	**0.05**	0.47***	±	0.05	**1.13^{+++}**	±	**0.02**
ventral Reticular Thalamus (vRT)	**1.73**	±	**0.06**	**1.53**	±	**0.03**	0.57^{***+++}	±	0.03
Basolateral Amygdala (BLA)	**1.66**	±	**0.06**	**1.62**	±	**0.03**	**1.21**	±	**0.04**
Medial Amygdala (MeA)	1.01	±	0.09	0.71	±	0.05	**2.03^{***+++}**	±	**0.01**
Central Amygdala (CeA)	0.76	±	0.05	**1.14**	±	**0.05**	0.43^{+++}	±	0.03
Retrosplenial Cortex (RSC)	0.62	±	0.07	0.58	±	0.04	0.77	±	0.03
Habenula (Hab)	0.86	±	0.10	**1.21**	±	**0.04**	**1.32***	±	**0.02**
Cornu Ammonis 1 (DHCA1)	1.01	±	0.10	0.83	±	0.05	**1.24^{+++}**	±	**0.02**
Cornu Ammonis 2 (DHCA2)	0.80	±	0.08	0.92	±	0.05	0.94	±	0.04
Dentate Gyrus (DHDG)	**1.50**	±	**0.04**	0.86***	±	0.04	**1.03**	±	**0.03**
Molecular Layer (DHML)	0.58	±	0.10	0.52	±	0.08	0.39	±	0.03
Auditory Cortex (AudC)	1.02	±	0.08	0.78	±	0.02	**1.31^{+++}**	±	**0.01**
Medial Geniculate (MG)	0.58	±	0.06	0.82	±	0.03	**1.64^{***+++}**	±	**0.02**
Dorsal Subiculum (DS)	0.50	±	0.09	0.85	±	0.04	0.60	±	0.03
VH Cornu Ammonis 1 (VHCA1)	0.57	±	0.07	**1.25**	±	**0.08**	**1.69*** **	±	**0.02**
VH Cornu Ammonis 2 (VHCA2)	0.78	±	0.10	**1.27**	±	**0.05**	**1.13*** **	±	**0.04**
VH Cornu Ammonis 3 (VHCA3)	0.62	±	0.08	0.54	±	0.11	**1.11^{**+}**	±	**0.04**
VH Dentate Gyrus (VHDG)	0.96	±	0.07	0.80	±	0.06	**1.70^{***+++}**	±	**0.01**
VH Molecular Layer (VHML)	**1.12**	±	**0.07**	0.80	±	0.05	0.39***	±	0.03
Mamillary Body (MB)	0.80	±	0.09	**1.18**	±	**0.03**	0.72^{+++}	±	0.04
Ventral Tegmental Area (VTA)	0.65	±	0.07	0.81	±	0.04	0.31	±	0.05
Substantia Nigra pars Compacta (SNC)	0.90	±	0.09	0.99	±	0.04	0.55	±	0.04
Substantia Nigra pars Reticulata (SNR)	0.58	±	0.04	**1.27**	±	**0.03**	0.44^{+++}	±	0.02
Dorsal Raphé (DR)	0.59	±	0.04	0.72	±	0.07	0.37	±	0.02
Median Raphé (MR)	**1.57**	±	**0.03**	0.56***	±	0.08	0.57***	±	0.03
Ventral Tegmental Nucleus (VTg)	0.99	±	0.05	0.75	±	0.05	**1.10^{++}**	±	**0.03**
Perirhinal Cortex (PRh)	0.57	±	0.12	0.86	±	0.02	0.44	±	0.04
anterior Reticular Thalamus (RT)	0.43	±	0.07	0.44	±	0.05	0.97	±	0.04
Entorhinal Cortex (EC)	**1.45**	±	**0.05**	0.60***	±	0.09	0.44***	±	0.03

*Table S7. Data shown as the Mean ± SEM variable importance to the projection (VIP) statistic. VIPs with a 95% confidence interval (CI) >1.0 are considered to be connected to the Re ("seed") region (bold). *p<0.05, **p<0.01 and ***p<0.001 significant difference from saline-treated WT mice (t-test with Bonferroni correction). $^{+}$p<0.05, $^{++}$p<0.01 and $^{+++}$p<0.001 significant difference from saline-treated Nrxn1α$^{+/-}$ mice (t-test with Bonferroni correction).*

Table S8. PLSR defined connectivity of the centromedial thalamus (CM) in saline-treated wild-type (WT) and *Nrxn1α*$^{+/-}$ mice and ketamine-treated *Nrxn1α*$^{+/-}$ mice

Genotype	Wild-type (WT)			Neurexin1α Hz			Neurexin1α Hz		
**Treatment**	**Saline**			**Saline**			**Ketamine**		
**Region**	**Mean ± SE**			**Mean ± SE**			**Mean ± SE**		
anterior Prelimbic Cortex (aPrL)	**1.69**	±	**0.05**	0.73***	±	0.06	0.84***	±	0.04
Frontal Association Area (FRA)	0.63	±	0.08	0.59	±	0.10	0.86	±	0.04
Dorsolateral Orbital Cortex (DLO)	**1.54**	±	**0.09**	0.63**	±	0.11	0.50***	±	0.02
Ventral Orbital Cortex (VO)	0.56	±	0.03	0.95	±	0.10	**1.15*****	±	**0.02**
Medial Orbital Cortex (MO)	0.57	±	0.07	0.64	±	0.06	0.33	±	0.02
medial Prelimbic Cortex (mPrL)	**1.17**	±	**0.03**	**1.58**	±	**0.09**	0.73***$^{+++}$	±	0.05
Infralimbic Cortex (IL)	0.74	±	0.07	**1.70**	±	**0.10**	0.32^{+++}	±	0.03
Nucleus Accumbens Core (NaC)	0.71	±	0.07	**1.20**	±	**0.05**	0.70^{++}	±	0.06
Nucleus Accumbens Shell (NaS)	0.62	±	0.05	0.50	±	0.06	0.45	±	0.04
Cingualte Cortex (Cg1)	0.35	±	0.08	0.66	±	0.05	0.38	±	0.02
Motor Cortex (M1)	0.66	±	0.09	0.68	±	0.04	**1.31**$^{+++}$**	±	**0.05**
Piriform Cortex (Piri)	0.48	±	0.06	**1.20**	±	**0.10**	**1.89*****	±	**0.02**
Insular Cortex (Ins)	0.36	±	0.05	**1.11**	±	**0.07**	**1.31*****	±	**0.02**
Ventromedial Striatum (VMST)	0.60	±	0.09	0.98	±	0.07	0.26	±	0.05
Dorsolateral Striatum (DLST)	**1.10**	±	**0.08**	0.84	±	0.08	0.38***	±	0.03
Medial Septum (MS)	0.34	±	0.02	0.77	±	0.09	0.95	±	0.04
Lateral Septum (LS)	0.48	±	0.04	0.83	±	0.06	**1.05*****	±	**0.04**
Ventral Limb of the Diagonal Band of Broca (VDB)	0.52	±	0.07	0.95	±	0.06	**1.19*****	±	**0.03**
Horizontal Limb of the Diagonal Band of Broca (HDB)	**1.12**	±	**0.07**	0.67	±	0.09	0.49***	±	0.02
Anteromedial Thalamus (AM)	0.95	±	0.12	**1.20**	±	**0.08**	0.95	±	0.04
Anteroventral Thalamus (AV)	1.00	±	0.10	0.68	±	0.11	0.54	±	0.04
Somatosensory Cortex (SSCTX)	0.43	±	0.10	**1.30**	±	**0.06**	0.78^{+++}	±	0.05
Globus Pallidus (GP)	0.37	±	0.05	0.66	±	0.05	0.90	±	0.05
Mediodorsal Thalamus (MD)	**1.53**	±	**0.08**	**1.08**	±	**0.08**	**1.03**	±	**0.04**
Centrolateral Thalamus (CL)	**1.53**	±	**0.07**	**1.43**	±	**0.07**	**1.14**	±	**0.05**
Ventrolateral Thalamus (VL)	0.56	±	0.07	0.75	±	0.04	**1.14**$^{+}$**	±	**0.06**
Ventromedial Thalamus (VM)	0.93	±	0.09	0.83	±	0.06	**1.71***$^{+++}$**	±	**0.03**
Nucleus Reuniens (Re)	**1.84**	±	**0.03**	0.90***	±	0.06	**1.50^{+++}**	±	**0.01**
dorsal Reticular Thalamus (dRT)	**1.91**	±	**0.03**	**1.62**	±	**0.07**	**1.91**	±	**0.02**
ventral Reticular Thalamus (vRT)	**1.83**	±	**0.04**	0.92***	±	0.04	**1.38^{+++}**	±	**0.02**
Basolateral Amygdala (BLA)	**1.45**	±	**0.07**	0.77***	±	0.06	0.51***	±	0.04
Medial Amygdala (MeA)	0.83	±	0.09	0.71	±	0.04	**1.50***$^{+++}$**	±	**0.02**
Central Amygdala (CeA)	0.94	±	0.07	0.55	±	0.10	0.35	±	0.03
Retrosplenial Cortex (RSC)	**1.24**	±	**0.11**	**1.10**	±	**0.08**	0.51***$^{+++}$	±	0.02
Habenula (Hab)	0.92	±	0.12	**1.53**	±	**0.04**	**1.28**	±	**0.06**
Cornu Ammonis 1 (DHCA1)	1.05	±	0.10	**1.41**	±	**0.09**	**1.77*****	±	**0.01**
Cornu Ammonis 2 (DHCA2)	0.83	±	0.06	0.86	±	0.05	**1.04**	±	**0.04**
Dentate Gyrus (DHDG)	**1.40**	±	**0.08**	**1.26**	±	**0.09**	0.92*	±	0.05
Molecular Layer (DHML)	0.88	±	0.05	0.87	±	0.08	0.66	±	0.05
Auditory Cortex (AudC)	0.82	±	0.03	0.73	±	0.17	**1.33*****	±	**0.05**
Medial Geniculate (MG)	0.51	±	0.16	0.97	±	0.08	**1.06**	±	**0.03**
Dorsal Subiculum (DS)	0.44	±	0.09	0.99	±	0.07	0.42	±	0.04
VH Cornu Ammonis 1 (VHCA1)	0.69	±	0.06	0.50	±	0.08	**1.14***$^{+++}$**	±	**0.02**
VH Cornu Ammonis 2 (VHCA2)	0.94	±	0.08	0.69	±	0.07	0.74	±	0.03
VH Cornu Ammonis 3 (VHCA3)	0.73	±	0.11	0.84	±	0.07	**1.04**	±	**0.04**
VH Dentate Gyrus (VHDG)	**1.07**	±	**0.09**	0.95	±	0.06	**1.01**	±	**0.03**
VH Molecular Layer (VHML)	0.84	±	0.09	0.98	±	0.08	0.99	±	0.03
Mamillary Body (MB)	0.23	±	0.03	0.99	±	0.10	0.74	±	0.05
Ventral Tegmental Area (VTA)	0.45	±	0.08	**1.20**	±	**0.12**	**1.02*****	±	**0.03**
Substantia Nigra pars Compacta (SNC)	**1.25**	±	**0.05**	0.84*	±	0.06	0.44***	±	0.04
Substantia Nigra pars Reticulata (SNR)	0.75	±	0.08	0.59	±	0.06	0.49	±	0.03
Dorsal Raphé (DR)	1.02	±	0.09	0.68	±	0.10	0.62	±	0.03
Median Raphé (MR)	**1.17**	±	**0.08**	0.87	±	0.05	0.74**	±	0.03
Ventral Tegmental Nucleus (VTg)	0.70	±	0.05	**1.22**	±	**0.08**	0.72^{++}	±	0.04
Perirhinal Cortex (PRh)	0.35	±	0.10	0.78	±	0.16	0.67	±	0.05
anterior Reticular Thalamus (RT)	0.89	±	0.09	0.42	±	0.10	**1.08^{+++}**	±	**0.03**
Entorhinal Cortex (EC)	0.94	±	0.05	0.80	±	0.06	0.65	±	0.00

*Table S8. Data shown as the Mean ± SEM variable importance to the projection (VIP) statistic. VIPs with a 95% confidence interval (CI) >1.0 are considered to be connected to the CM ("seed") region (bold). *p<0.05, **p<0.01 and ***p<0.001 significant difference from saline-treated WT mice (t-test with Bonferroni correction). $^{+}$p<0.05, $^{++}$p<0.01 and $^{+++}$p<0.001 significant difference from saline-treated Nrxn1α$^{+/-}$ mice (t-test with Bonferroni correction).*

Table S9. PLSR defined connectivity of the ventral limb of the diagonal band of Broca (VDB) in saline-treated wild-type (WT) and *Nrxn1α*$^{+/-}$ mice.

**Genotype**	**Wild-type (WT)**			***Neurexin1 α*** $^{+/-}$		
**Treatment**	**Saline**			**Saline**		
**Region**	**Mean ± SEM**			**Mean ± SEM**		
anterior Prelimbic Cortex (aPrL)	0.42	±	0.08	0.45	±	0.06
Frontal Association Area (FRA)	0.67	±	0.10	**1.38****	±	**0.05**
Dorsolateral Orbital Cortex (DLO)	0.93	±	0.06	0.95	±	0.08
Ventral Orbital Cortex (VO)	0.84	±	0.10	**1.42****	±	**0.02**
Medial Orbital Cortex (MO)	0.35	±	0.07	0.42	±	0.06
medial Prelimbic Cortex (mPrL)	**1.31**	±	**0.11**	0.49	±	0.04
Infralimbic Cortex (IL)	**1.40**	±	**0.13**	0.50	±	0.06
Nucleus Accumbens Core (NaC)	0.86	±	0.08	**1.19**	±	**0.03**
Nucleus Accumbens Shell (NaS)	0.47	±	0.09	**1.34****	±	**0.02**
Cingualte Cortex (Cg1)	**1.43**	±	**0.12**	**1.03**	±	**0.06**
Motor Cortex (M1)	0.86	±	0.08	**1.08**	±	**0.03**
Piriform Cortex (Piri)	0.56	±	0.13	**1.13**	±	**0.07**
Insular Cortex (Ins)	**1.42**	±	**0.06**	**1.25**	±	**0.02**
Ventromedial Striatum (VMST)	0.45	±	0.09	0.69	±	0.08
Dorsolateral Striatum (DLST)	0.38	±	0.06	0.99	±	0.05
Medial Septum (MS)	**1.65**	±	**0.07**	**1.47**	±	**0.03**
Lateral Septum (LS)	**1.93**	±	**0.09**	**1.20**	±	**0.02**
Horizontal Limb of the Diagonal Band of Broca (HDB)	**1.55**	±	**0.06**	**1.45**	±	**0.02**
Anteromedial Thalamus (AM)	0.79	±	0.12	**1.14**	±	**0.05**
Anteroventral Thalamus (AV)	1.02	±	0.07	0.81	±	0.05
Somatosensory Cortex (SSCTX)	0.50	±	0.07	0.66	±	0.04
Globus Pallidus (GP)	0.88	±	0.13	**1.24**	±	**0.06**
Mediodorsal Thalamus (MD)	0.54	±	0.11	0.56	±	0.06
Centromedial Thalamus (CM)	0.61	±	0.07	0.69	±	0.05
Centrolateral Thalamus (CL)	**1.40**	±	**0.07**	0.44	±	0.09
Ventrolateral Thalamus (VL)	**1.23**	±	**0.11**	0.97	±	0.05
Ventromedial Thalamus (VM)	0.88	±	0.12	0.70	±	0.09
Nucleus Reuniens (Re)	0.83	±	0.08	0.86	±	0.05
dorsal Reticular Thalamus (dRT)	0.47	±	0.04	0.79	±	0.06
ventral Reticular Thalamus (vRT)	**1.15**	±	**0.07**	0.68	±	0.08
Basolateral Amygdala (BLA)	0.38	±	0.06	0.79	±	0.06
Medial Amygdala (MeA)	0.87	±	0.09	0.74	±	0.04
Central Amygdala (CeA)	**1.51**	±	**0.05**	**1.18**	±	**0.07**
Retrosplenial Cortex (RSC)	0.67	±	0.11	0.43	±	0.05
Habenula (Hab)	**1.20**	±	**0.12**	0.84	±	0.06
Cornu Ammonis 1 (DHCA1)	0.48	±	0.04	**1.55*****	±	**0.01**
Cornu Ammonis 2 (DHCA2)	**1.09**	±	**0.09**	**1.36**	±	**0.07**
Dentate Gyrus (DHDG)	0.86	±	0.11	**1.21**	±	**0.05**
Molecular Layer (DHML)	0.44	±	0.17	0.74	±	0.07
Auditory Cortex (AudC)	0.89	±	0.12	**1.21**	±	**0.07**
Medial Geniculate (MG)	0.52	±	0.06	**1.28*****	±	**0.05**
Dorsal Subiculum (DS)	0.87	±	0.10	**1.29**	±	**0.02**
VH Cornu Ammonis 1 (VHCA1)	**1.32**	±	**0.07**	0.96	±	0.07
VH Cornu Ammonis 2 (VHCA2)	**1.23**	±	**0.10**	**1.12**	±	**0.06**
VH Cornu Ammonis 3 (VHCA3)	0.64	±	0.11	0.52	±	0.09
VH Dentate Gyrus (VHDG)	0.64	±	0.10	0.64	±	0.04
VH Molecular Layer (VHML)	**1.51**	±	**0.13**	**1.16**	±	**0.04**
Mamillary Body (MB)	0.96	±	0.12	0.81	±	0.05
Ventral Tegmental Area (VTA)	1.02	±	0.08	0.78	±	0.05
Substantia Nigra pars Compacta (SNC)	0.77	±	0.07	0.70	±	0.05
Substantia Nigra pars Reticulata (SNR)	0.83	±	0.07	**1.35*****	±	**0.03**
Dorsal Raphé (DR)	**1.03**	±	**0.09**	0.26	±	0.06
Median Raphé (MR)	0.57	±	0.12	0.43	±	0.05
Ventral Tegmental Nucleus (VTg)	0.72	±	0.11	**1.24**	±	**0.02**
Perirhinal Cortex (PRh)	0.66	±	0.08	**1.35****	±	**0.07**
anterior Reticular Thalamus (RT)	0.35	±	0.10	0.38	±	0.07
Entorhinal Cortex (EC)	0.62	±	0.13	0.34	±	0.09

*Data shown as the Mean ± SEM variable importance to the projection (VIP) statistic. VIPs with a 95% confidence interval (CI) >1.0 are considered to be connected to the VDB ("seed") region (bold). **denotes p<0.01 and ***denotes p<0.001 significant difference from saline-treated WT mice (t-test with Bonferroni correction).*

Table S10. PLSR defined connectivity of the horizontal limb of the diagonal band of Broca (HDB) in saline-treated wild-type (WT) and *Nrxn1α*$^{+/-}$ mice.

Genotype	Wild-type (WT)			*Neurexin1 α* $^{+/-}$		
Treatment	Saline			Saline		
Region	Mean	±	SEM	Mean	±	SEM
anterior Prelimbic Cortex (aPrL)	0.51	±	0.03	**1.15*****	±	**0.04**
Frontal Association Area (FRA)	0.49	±	0.11	**1.51*****	±	**0.02**
Dorsolateral Orbital Cortex (DLO)	**1.63**	±	**0.04**	**1.36**	±	**0.06**
Ventral Orbital Cortex (VO)	0.62	±	0.07	**1.03**	±	**0.06**
Medial Orbital Cortex (MO)	0.48	±	0.19	0.39	±	0.03
medial Prelimbic Cortex (mPrL)	0.55	±	0.08	0.92	±	0.09
Infralimbic Cortex (IL)	0.49	±	0.10	0.75	±	0.09
Nucleus Accumbens Core (NaC)	**1.09**	±	**0.09**	0.99	±	0.04
Nucleus Accumbens Shell (NaS)	0.81	±	0.08	**1.42*****	±	**0.02**
Cingualte Cortex (Cg1)	**1.26**	±	**0.09**	0.90	±	0.06
Motor Cortex (M1)	0.78	±	0.09	**1.14**	±	**0.04**
Piriform Cortex (Piri)	0.43	±	0.05	0.92	±	0.05
Insular Cortex (Ins)	0.65	±	0.11	0.73	±	0.06
Ventromedial Striatum (VMST)	**1.32**	±	**0.04**	0.64	±	0.09
Dorsolateral Striatum (DLST)	**1.54**	±	**0.07**	**1.34**	±	**0.04**
Medial Septum (MS)	**1.35**	±	**0.07**	**1.68**	±	**0.02**
Lateral Septum (LS)	**1.54**	±	**0.04**	**1.55**	±	**0.03**
Vertical Limb of the Diagonal Band of Broca (VDB)	**1.40**	±	**0.04**	**1.49**	±	**0.02**
Anteromedial Thalamus (AM)	0.73	±	0.15	**1.57***	±	**0.03**
Anteroventral Thalamus (AV)	0.52	±	0.10	**1.28****	±	**0.06**
Somatosensory Cortex (SSCTX)	0.78	±	0.07	0.38	±	0.03
Globus Pallidus (GP)	0.42	±	0.09	**1.17*****	±	**0.05**
Mediodorsal Thalamus (MD)	0.38	±	0.08	0.50	±	0.06
Centromedial Thalamus (CM)	**1.13**	±	**0.08**	0.51	±	0.09
Centrolateral Thalamus (CL)	0.99	±	0.08	0.38	±	0.08
Ventrolateral Thalamus (VL)	0.52	±	0.07	1.02	±	0.04
Ventromedial Thalamus (VM)	0.64	±	0.08	0.65	±	0.06
Nucleus Reuniens (Re)	0.91	±	0.09	**1.37**	±	**0.05**
dorsal Reticular Thalamus (dRT)	0.60	±	0.06	0.53	±	0.05
ventral Reticular Thalamus (vRT)	0.87	±	0.11	1.01	±	0.06
Basolateral Amygdala (BLA)	0.78	±	0.04	**1.04**	±	**0.06**
Medial Amygdala (MeA)	**1.51**	±	**0.05**	0.59	±	0.03
Central Amygdala (CeA)	**1.80**	±	**0.04**	**1.55**	±	**0.03**
Retrosplenial Cortex (RSC)	0.38	±	0.10	0.28	±	0.06
Habenula (Hab)	**1.29**	±	**0.07**	**1.05**	±	**0.06**
Cornu Ammonis 1 (DHCA1)	0.79	±	0.11	0.88	±	0.09
Cornu Ammonis 2 (DHCA2)	**1.44**	±	**0.10**	**1.23**	±	**0.04**
Dentate Gyrus (DHDG)	**1.58**	±	**0.05**	0.78	±	0.07
Molecular Layer (DHML)	0.45	±	0.05	0.65	±	0.08
Auditory Cortex (AudC)	1.01	±	0.08	**1.32**	±	**0.04**
Medial Geniculate (MG)	0.45	±	0.09	**1.07***	±	**0.06**
Dorsal Subiculum (DS)	0.66	±	0.16	0.82	±	0.06
VH Cornu Ammonis 1 (VHCA1)	0.35	±	0.13	0.73	±	0.05
VH Cornu Ammonis 2 (VHCA2)	0.37	±	0.04	0.92	±	0.04
VH Cornu Ammonis 3 (VHCA3)	0.54	±	0.14	0.53	±	0.05
VH Dentate Gyrus (VHDG)	0.92	±	0.11	0.46	±	0.04
VH Molecular Layer (VHML)	**1.46**	±	**0.05**	0.71	±	0.05
Mamillary Body (MB)	0.51	±	0.06	0.61	±	0.04
Ventral Tegmental Area (VTA)	0.44	±	0.08	0.56	±	0.04
Substantia Nigra pars Compacta (SNC)	**1.57**	±	**0.09**	0.58	±	0.03
Substantia Nigra pars Reticulata (SNR)	**1.63**	±	**0.06**	**1.32**	±	**0.03**
Dorsal Raphé (DR)	0.52	±	0.10	0.26	±	0.04
Median Raphé (MR)	0.66	±	0.07	0.65	±	0.07
Ventral Tegmental Nucleus (VTg)	0.51	±	0.12	0.69	±	0.06
Perirhinal Cortex (PRh)	0.95	±	0.09	**1.35**	±	**0.03**
anterior Reticular Thalamus (RT)	0.87	±	0.09	0.68	±	0.05
Entorhinal Cortex (EC)	0.87	±	0.10	0.47	±	0.05

*Table S10. Data shown as the Mean ± SEM variable importance to the projection (VIP) statistic. VIPs with a 95% confidence interval (CI) >1.0 are considered to be connected to the HDB ("seed") region (bold). *denotes p<0.05, **denotes p<0.01 and ***denotes p<0.001 significant difference from saline-treated WT mice (t-test with Bonferroni correction).*

Table S11. PLSR defined connectivity of the lateral septum (LS) in saline-treated wild-type (WT) and *Nrxn1α*$^{+/-}$ mice

Genotype	Wild-type (WT)			*Neurexin1 α* $^{+/-}$		
**Treatment**	**Saline**			**Saline**		
**Region**	**Mean ± SEM**			**Mean ± SEM**		
anterior Prelimbic Cortex (aPrL)	0.72	±	0.08	**1.04**	±	**0.06**
Frontal Association Area (FRA)	0.42	±	0.04	**1.29*** **	±	**0.04**
Dorsolateral Orbital Cortex (DLO)	0.77	±	0.07	**0.90**	±	**0.09**
Ventral Orbital Cortex (VO)	0.89	±	0.13	0.68	±	0.03
Medial Orbital Cortex (MO)	0.61	±	0.07	0.78	±	0.06
medial Prelimbic Cortex (mPrL)	**1.10**	±	**0.09**	**1.11**	±	**0.08**
Infralimbic Cortex (IL)	**1.07**	±	**0.11**	**1.13**	±	**0.08**
Nucleus Accumbens Core (NaC)	0.68	±	0.08	**1.02**	±	**0.05**
Nucleus Accumbens Shell (NaS)	0.56	±	0.10	**1.20** **	±	**0.04**
Cingualte Cortex (Cg1)	**1.72**	±	**0.08**	0.85	±	0.04
Motor Cortex (M1)	0.47	±	0.04	**1.14*** **	±	**0.05**
Piriform Cortex (Piri)	0.57	±	0.10	0.71	±	0.02
Insular Cortex (Ins)	0.52	±	0.11	0.88	±	0.06
Ventromedial Striatum (VMST)	**1.03**	±	**0.12**	0.49	±	0.06
Dorsolateral Striatum (DLST)	0.91	±	0.08	0.81	±	0.07
Medial Septum (MS)	**1.93**	±	**0.04**	**1.65**	±	**0.02**
Vertical Limb of the Diagonal Band of Broca (VDB)	**1.87**	±	**0.08**	**1.26**	±	**0.02**
Horizontal Limb of the Diagonal Band of Broca (HDB)	**1.65**	±	**0.04**	**1.55**	±	**0.03**
Anteromedial Thalamus (AM)	0.77	±	0.07	**1.31** **	±	**0.05**
Anteroventral Thalamus (AV)	**1.14**	±	**0.08**	0.94	±	0.08
Somatosensory Cortex (SSCTX)	0.59	±	0.06	0.64	±	0.05
Globus Pallidus (GP)	**1.08**	±	**0.11**	**1.38**	±	**0.02**
Mediodorsal Thalamus (MD)	0.52	±	0.05	0.55	±	0.06
Centromedial Thalamus (CM)	0.55	±	0.06	0.72	±	0.06
Centrolateral Thalamus (CL)	**1.02**	±	**0.06**	0.95	±	0.07
Ventrolateral Thalamus (VL)	0.73	±	0.11	**1.37** **	±	**0.02**
Ventromedial Thalamus (VM)	0.43	±	0.09	**1.02** **	±	**0.04**
Nucleus Reuniens (Re)	0.64	±	0.07	**1.30*** **	±	**0.04**
dorsal Reticular Thalamus (dRT)	0.72	±	0.04	0.46	±	0.03
ventral Reticular Thalamus (vRT)	0.39	±	0.08	**1.12*** **	±	**0.06**
Basolateral Amygdala (BLA)	0.46	±	0.06	**1.06*** **	±	**0.05**
Medial Amygdala (MeA)	**1.60**	±	**0.06**	0.65	±	0.07
Central Amygdala (CeA)	**1.68**	±	**0.08**	**1.31**	±	**0.04**
Retrosplenial Cortex (RSC)	0.85	±	0.05	0.86	±	0.03
Habenula (Hab)	0.94	±	0.11	**1.35**	±	**0.04**
Cornu Ammonis 1 (DHCA1)	0.50	±	0.07	0.82	±	0.06
Cornu Ammonis 2 (DHCA2)	**1.55**	±	**0.05**	**1.16**	±	**0.03**
Dentate Gyrus (DHDG)	**1.20**	±	**0.05**	0.81	±	0.06
Molecular Layer (DHML)	0.76	±	0.09	0.41	±	0.10
Auditory Cortex (AudC)	**1.50**	±	**0.05**	**1.20**	±	**0.04**
Medial Geniculate (MG)	0.43	±	0.05	**1.23*** **	±	**0.02**
Dorsal Subiculum (DS)	**1.23**	±	**0.10**	0.95	±	0.06
VH Cornu Ammonis 1 (VHCA1)	0.56	±	0.06	0.64	±	0.02
VH Cornu Ammonis 2 (VHCA2)	0.30	±	0.04	0.78	±	0.02
VH Cornu Ammonis 3 (VHCA3)	0.73	±	0.11	0.66	±	0.04
VH Dentate Gyrus (VHDG)	0.50	±	0.09	0.52	±	0.07
VH Molecular Layer (VHML)	**1.93**	±	**0.05**	**0.89**	±	**0.05**
Mamillary Body (MB)	0.50	±	0.18	0.64	±	0.06
Ventral Tegmental Area (VTA)	0.48	±	0.04	0.86	±	0.06
Substantia Nigra pars Compacta (SNC)	0.65	±	0.08	0.71	±	0.04
Substantia Nigra pars Reticulata (SNR)	**1.23**	±	**0.08**	**1.58**	±	**0.01**
Dorsal Raphé (DR)	0.76	±	0.13	0.75	±	0.07
Median Raphé (MR)	0.91	±	0.06	0.36	±	0.06
Ventral Tegmental Nucleus (VTg)	0.58	±	0.05	0.82	±	0.06
Perirhinal Cortex (PRh)	0.36	±	0.03	**1.22*** **	±	**0.03**
anterior Reticular Thalamus (RT)	0.58	±	0.07	0.37	±	0.04
Entorhinal Cortex (EC)	0.83	±	0.08	0.36	±	0.09

*Table S11. Data shown as the Mean ± SEM variable importance to the projection (VIP) statistic. VIPs with a 95% confidence interval (CI) >1.0 are considered to be connected to the LS ("seed") region (bold). **denotes p<0.01 and ***denotes p<0.001 significant difference from saline-treated WT mice (t-test with Bonferroni correction).*

Table S12. PLSR defined connectivity of the medial septum (MS) in saline-treated wild-type (WT) and *Nrxn1α*$^{+/-}$ mice

Genotype	Wild-type (WT)			*Neurexin1 α* $^{+/-}$		
Treatment	Saline			Saline		
Region	Mean ± SEM			Mean ± SEM		
anterior Prelimbic Cortex (aPrL)	**1.08**	±	**0.11**	0.58	±	0.05
Frontal Association Area (FRA)	0.91	±	0.08	**1.51***	±	**0.02**
Dorsolateral Orbital Cortex (DLO)	0.97	±	0.03	**1.29***	±	**0.05**
Ventral Orbital Cortex (VO)	0.70	±	0.10	0.88	±	0.08
Medial Orbital Cortex (MO)	**1.38**	±	**0.14**	0.37	±	0.05
medial Prelimbic Cortex (mPrL)	**1.19**	±	**0.10**	0.80	±	0.08
Infralimbic Cortex (IL)	0.71	±	0.10	0.75	±	0.10
Nucleus Accumbens Core (NaC)	0.44	±	0.10	0.94	±	0.04
Nucleus Accumbens Shell (NaS)	0.59	±	0.14	**1.26***	±	**0.03**
Cingualte Cortex (Cg1)	**1.54**	±	**0.09**	1.01	±	0.06
Motor Cortex (M1)	0.43	±	0.11	**1.03***	±	**0.04**
Piriform Cortex (Piri)	0.87	±	0.05	0.74	±	0.05
Insular Cortex (Ins)	0.66	±	0.14	0.86	±	0.06
Ventromedial Striatum (VMST)	**1.38**	±	**0.17**	0.51	±	0.09
Dorsolateral Striatum (DLST)	0.67	±	0.07	**1.21***	±	**0.05**
Lateral Septum (LS)	**2.05**	±	**0.05**	**1.62**	±	**0.02**
Vertical Limb of the Diagonal Band of Broca (VDB)	**1.70**	±	**0.07**	**1.48**	±	**0.03**
Horizontal Limb of the Diagonal Band of Broca (HDB)	**1.55**	±	**0.07**	**1.65**	±	**0.02**
Anteromedial Thalamus (AM)	0.58	±	0.10	**1.56*** **	±	**0.02**
Anteroventral Thalamus (AV)	0.68	±	0.08	**1.17**	±	**0.06**
Somatosensory Cortex (SSCTX)	0.60	±	0.05	0.51	±	0.03
Globus Pallidus (GP)	0.49	±	0.14	**1.30***	±	**0.05**
Mediodorsal Thalamus (MD)	0.58	±	0.06	0.31	±	0.07
Centromedial Thalamus (CM)	0.44	±	0.03	0.63	±	0.08
Centrolateral Thalamus (CL)	0.59	±	0.07	0.58	±	0.10
Ventrolateral Thalamus (VL)	**1.18**	±	**0.12**	**1.20**	±	**0.04**
Ventromedial Thalamus (VM)	0.54	±	0.10	0.88	±	0.07
Nucleus Reuniens (Re)	0.58	±	0.04	**1.16*** **	±	**0.05**
dorsal Reticular Thalamus (dRT)	0.77	±	0.08	0.51	±	0.04
ventral Reticular Thalamus (vRT)	0.42	±	0.12	0.89	±	0.06
Basolateral Amygdala (BLA)	0.71	±	0.08	0.89	±	0.05
Medial Amygdala (MeA)	**1.16**	±	**0.15**	0.53	±	0.06
Central Amygdala (CeA)	**1.89**	±	**0.08**	**1.34**	±	**0.04**
Retrosplenial Cortex (RSC)	0.40	±	0.10	0.59	±	0.04
Habenula (Hab)	**1.13**	±	**0.11**	**1.20**	±	**0.05**
Cornu Ammonis 1 (DHCA1)	0.43	±	0.05	0.90	±	0.09
Cornu Ammonis 2 (DHCA2)	**1.55**	±	**0.06**	**1.37**	±	**0.04**
Dentate Gyrus (DHDG)	0.70	±	0.06	0.90	±	0.08
Molecular Layer (DHML)	0.60	±	0.11	0.68	±	0.07
Auditory Cortex (AudC)	0.82	±	0.12	**1.47***	±	**0.03**
Medial Geniculate (MG)	0.43	±	0.07	**1.24*** **	±	**0.06**
Dorsal Subiculum (DS)	0.99	±	0.12	0.88	±	0.05
VH Cornu Ammonis 1 (VHCA1)	0.99	±	0.08	0.54	±	0.04
VH Cornu Ammonis 2 (VHCA2)	0.77	±	0.12	0.93	±	0.04
VH Cornu Ammonis 3 (VHCA3)	0.72	±	0.07	0.53	±	0.05
VH Dentate Gyrus (VHDG)	0.42	±	0.03	0.43	±	0.05
VH Molecular Layer (VHML)	**1.51**	±	**0.09**	0.92	±	0.06
Mamillary Body (MB)	**1.08**	±	**0.10**	0.65	±	0.07
Ventral Tegmental Area (VTA)	0.82	±	0.11	0.64	±	0.07
Substantia Nigra pars Compacta (SNC)	0.71	±	0.09	0.54	±	0.05
Substantia Nigra pars Reticulata (SNR)	**1.65**	±	**0.10**	**1.47**	±	**0.02**
Dorsal Raphé (DR)	0.68	±	0.11	0.39	±	0.05
Median Raphé (MR)	0.46	±	0.12	0.57	±	0.05
Ventral Tegmental Nucleus (VTg)	0.88	±	0.10	0.80	±	0.07
Perirhinal Cortex (PRh)	0.72	±	0.04	**1.53*** **	±	**0.02**
anterior Reticular Thalamus (RT)	0.47	±	0.06	0.42	±	0.03
Entorhinal Cortex (EC)	0.73	±	0.09	0.51	±	0.04

*Table S12. Data shown as the Mean ± SEM variable importance to the projection (VIP) statistic. VIPs with a 95% confidence interval (CI) >1.0 are considered to be connected to the MS ("seed") region (bold). *denotes p<0.05 and ***denotes p<0.001 significant difference from saline-treated WT mice (t-test with Bonferroni correction).*

Table S13. PLSR defined connectivity of the nucleus accumbens shell (NacS) in saline-treated wild-type (WT) and *Nrxn1α*$^{+/-}$ mice

**Genotype**	**Wild-type (WT)**			***Neurexin1 α*** $^{+/-}$		
**Treatment**	**Saline**			**Saline**		
**Region**	**Mean ± SEM**			**Mean ± SEM**		
anterior Prelimbic Cortex (aPrL)	0.65	±	0.06	0.43	±	0.06
Frontal Association Area (FRA)	0.51	±	0.13	0.93	±	0.06
Dorsolateral Orbital Cortex (DLO)	**1.15**	±	**0.09**	0.64	±	0.10
Ventral Orbital Cortex (VO)	0.53	±	0.10	0.78	±	0.07
Medial Orbital Cortex (MO)	0.86	±	0.14	0.64	±	0.06
medial Prelimbic Cortex (mPrL)	0.49	±	0.10	**1.24****	±	**0.07**
Infralimbic Cortex (IL)	0.90	±	0.09	**1.05**	±	**0.09**
Nucleus Accumbens Core (NaC)	**1.64**	±	**0.09**	**1.48**	±	**0.04**
Cingualte Cortex (Cg1)	0.81	±	0.06	**1.27***	±	**0.06**
Motor Cortex (M1)	0.55	±	0.12	0.65	±	0.04
Piriform Cortex (Piri)	**1.39**	±	**0.10**	**1.30**	±	**0.05**
Insular Cortex (Ins)	**1.65**	±	**0.07**	**1.13**	±	**0.05**
Ventromedial Striatum (VMST)	0.56	±	0.08	0.65	±	0.06
Dorsolateral Striatum (DLST)	0.54	±	0.11	0.86	±	0.07
Medial Septum (MS)	0.58	±	0.15	**1.30***	±	**0.02**
Lateral Septum (LS)	0.56	±	0.09	**1.20*****	±	**0.03**
Vertical Limb of the Diagonal Band of Broca (VDB)	0.48	±	0.10	**1.38*****	±	**0.02**
Horizontal Limb of the Diagonal Band of Broca (HDB)	0.86	±	0.09	**1.41****	±	**0.02**
Anteromedial Thalamus (AM)	**1.15**	±	**0.09**	**1.03**	±	**0.04**
Anteroventral Thalamus (AV)	0.52	±	0.06	0.57	±	0.08
Somatosensory Cortex (SSCTX)	**1.48**	±	**0.07**	**1.23**	±	**0.07**
Globus Pallidus (GP)	**1.57**	±	**0.06**	**1.06**	±	**0.04**
Mediodorsal Thalamus (MD)	0.35	±	0.04	0.45	±	0.09
Centromedial Thalamus (CM)	0.65	±	0.05	0.37	±	0.06
Centrolateral Thalamus (CL)	0.44	±	0.04	0.69	±	0.08
Ventrolateral Thalamus (VL)	0.75	±	0.09	**1.17**	±	**0.05**
Ventromedial Thalamus (VM)	1.03	±	0.07	0.82	±	0.06
Nucleus Reuniens (Re)	0.43	±	0.08	**1.18*****	±	**0.05**
dorsal Reticular Thalamus (dRT)	0.41	±	0.06	0.49	±	0.04
ventral Reticular Thalamus (vRT)	0.94	±	0.08	**1.46***	±	**0.05**
Basolateral Amygdala (BLA)	0.55	±	0.12	**1.40****	±	**0.07**
Medial Amygdala (MeA)	0.94	±	0.10	0.78	±	0.06
Central Amygdala (CeA)	0.51	±	0.07	0.97	±	0.06
Retrosplenial Cortex (RSC)	0.36	±	0.08	0.55	±	0.11
Habenula (Hab)	0.59	±	0.06	**1.05**	±	**0.07**
Cornu Ammonis 1 (DHCA1)	0.66	±	0.07	1.02	±	0.05
Cornu Ammonis 2 (DHCA2)	0.80	±	0.10	1.00	±	0.04
Dentate Gyrus (DHDG)	0.67	±	0.09	0.91	±	0.03
Molecular Layer (DHML)	**1.03**	±	**0.07**	0.84	±	0.06
Auditory Cortex (AudC)	**1.10**	±	**0.07**	0.96	±	0.06
Medial Geniculate (MG)	**1.41**	±	**0.07**	**1.09**	±	**0.03**
Dorsal Subiculum (DS)	**1.49**	±	**0.06**	**1.17**	±	**0.06**
VH Cornu Ammonis 1 (VHCA1)	0.40	±	0.10	**1.25*****	±	**0.04**
VH Cornu Ammonis 2 (VHCA2)	0.99	±	0.11	**1.15**	±	**0.03**
VH Cornu Ammonis 3 (VHCA3)	**1.19**	±	**0.13**	0.45	±	0.09
VH Dentate Gyrus (VHDG)	0.52	±	0.11	0.63	±	0.06
VH Molecular Layer (VHML)	**1.03**	±	**0.06**	0.99	±	0.04
Mamillary Body (MB)	0.70	±	0.08	0.71	±	0.05
Ventral Tegmental Area (VTA)	**1.19**	±	**0.07**	0.81	±	0.03
Substantia Nigra pars Compacta (SNC)	**1.48**	±	**0.11**	0.93	±	0.03
Substantia Nigra pars Reticulata (SNR)	0.80	±	0.09	**1.17**	±	**0.03**
Dorsal Raphé (DR)	0.46	±	0.09	**1.14*****	±	**0.04**
Median Raphé (MR)	**1.39**	±	**0.07**	0.51	±	0.05
Ventral Tegmental Nucleus (VTg)	**1.23**	±	**0.10**	**1.12**	±	**0.05**
Perirhinal Cortex (PRh)	**1.93**	±	**0.05**	**1.08**	±	**0.04**
anterior Reticular Thalamus (RT)	**1.62**	±	**0.07**	0.39	±	0.06
Entorhinal Cortex (EC)	0.66	±	0.10	0.45	±	0.09

*Table S13. Data shown as the Mean ± SEM variable importance to the projection (VIP) statistic. VIPs with a 95% confidence interval (CI) >1.0 are considered to be connected to the NacS ("seed") region (bold). *denotes p<0.05, **denotes p<0.01 and ***denotes p<0.001 significant difference from saline-treated WT mice (t-test with Bonferroni correction).*

Table S14. PLSR defined connectivity of the substantia nigra pars reticulata (SNR) in saline-treated wild-type (WT) and *Nrxn1α*$^{+/-}$ mice

Genotype	Wild-type (WT)			*Neurexin1 α* $^{+/-}$		
**Treatment**	**Saline**			**Saline**		
**Region**	**Mean**	**±**	**SEM**	**Mean**	**±**	**SEM**
anterior Prelimbic Cortex (aPrL)	0.59	±	0.04	0.44	±	0.11
Frontal Association Area (FRA)	0.60	±	0.08	**1.40*** **	±	**0.03**
Dorsolateral Orbital Cortex (DLO)	1.02	±	0.08	0.82	±	0.07
Ventral Orbital Cortex (VO)	0.59	±	0.12	0.76	±	0.03
Medial Orbital Cortex (MO)	0.76	±	0.19	0.93	±	0.06
medial Prelimbic Cortex (mPrL)	0.56	±	0.07	0.48	±	0.06
Infralimbic Cortex (IL)	1.03	±	0.08	0.49	±	0.08
Nucleus Accumbens Core (NaC)	0.99	±	0.09	**1.18**	±	**0.04**
Nucleus Accumbens Shell (NaS)	0.77	±	0.09	**1.13**	±	**0.03**
Cingualte Cortex (Cg1)	0.52	±	0.08	0.89	±	0.03
Motor Cortex (M1)	**1.14**	±	**0.10**	1.02	±	0.06
Piriform Cortex (Piri)	0.84	±	0.10	0.85	±	0.04
Insular Cortex (Ins)	0.64	±	0.09	**1.10**	±	**0.04**
Ventromedial Striatum (VMST)	**1.79**	±	**0.06**	0.62	±	0.05
Dorsolateral Striatum (DLST)	**1.72**	±	**0.05**	0.75	±	0.05
Medial Septum (MS)	**1.57**	±	**0.10**	**1.47**	±	**0.02**
Lateral Septum (LS)	**1.22**	±	**0.08**	**1.55**	±	**0.02**
Vertical Limb of the Diagonal Band of Broca (VDB)	0.77	±	0.06	**1.37*** **	±	**0.03**
Horizontal Limb of the Diagonal Band of Broca (HDB)	**1.71**	±	**0.06**	**1.30**	±	**0.04**
Anteromedial Thalamus (AM)	0.84	±	0.10	1.01	±	0.05
Anteroventral Thalamus (AV)	0.36	±	0.04	0.90	±	0.05
Somatosensory Cortex (SSCTX)	**1.07**	±	**0.08**	0.55	±	0.04
Globus Pallidus (GP)	0.80	±	0.09	**1.48*** **	±	**0.03**
Mediodorsal Thalamus (MD)	0.63	±	0.06	0.32	±	0.08
Centromedial Thalamus (CM)	0.81	±	0.08	0.36	±	0.04
Centrolateral Thalamus (CL)	0.42	±	0.03	0.66	±	0.09
Ventrolateral Thalamus (VL)	0.42	±	0.04	**1.28*** **	±	**0.04**
Ventromedial Thalamus (VM)	0.33	±	0.06	0.97	±	0.06
Nucleus Reuniens (Re)	0.62	±	0.05	**1.12*** **	±	**0.04**
dorsal Reticular Thalamus (dRT)	0.48	±	0.05	0.54	±	0.05
ventral Reticular Thalamus (vRT)	0.58	±	0.09	0.75	±	0.06
Basolateral Amygdala (BLA)	0.89	±	0.12	1.02	±	0.05
Medial Amygdala (MeA)	**1.27**	±	**0.09**	0.99	±	0.05
Central Amygdala (CeA)	**1.34**	±	**0.09**	**1.34**	±	**0.03**
Retrosplenial Cortex (RSC)	0.28	±	0.05	0.43	±	0.07
Habenula (Hab)	**1.53**	±	**0.05**	0.94	±	0.05
Cornu Ammonis 1 (DHCA1)	0.46	±	0.05	1.00	±	0.04
Cornu Ammonis 2 (DHCA2)	**2.00**	±	**0.03**	**1.59**	±	**0.02**
Dentate Gyrus (DHDG)	0.87	±	0.06	**1.42*** **	±	**0.04**
Molecular Layer (DHML)	0.38	±	0.07	0.85	±	0.08
Auditory Cortex (AudC)	0.40	±	0.05	**1.18*** **	±	**0.06**
Medial Geniculate (MG)	0.98	±	0.08	**1.38**	±	**0.03**
Dorsal Subiculum (DS)	0.42	±	0.12	**1.10** **	±	**0.03**
VH Cornu Ammonis 1 (VHCA1)	0.53	±	0.07	0.61	±	0.02
VH Cornu Ammonis 2 (VHCA2)	0.32	±	0.07	0.80	±	0.02
VH Cornu Ammonis 3 (VHCA3)	1.01	±	0.12	0.34	±	0.04
VH Dentate Gyrus (VHDG)	**1.34**	±	**0.07**	**1.14**	±	**0.05**
VH Molecular Layer (VHML)	0.95	±	0.07	**1.06**	±	**0.06**
Mamillary Body (MB)	**1.34**	±	**0.13**	0.78	±	0.06
Ventral Tegmental Area (VTA)	0.49	±	0.08	**1.09** **	±	**0.05**
Substantia Nigra pars Compacta (SNC)	**1.73**	±	**0.05**	0.73	±	0.04
Dorsal Raphé (DR)	0.98	±	0.10	1.02	±	0.05
Median Raphé (MR)	0.42	±	0.05	0.41	±	0.04
Ventral Tegmental Nucleus (VTg)	0.53	±	0.15	1.00	±	0.05
Perirhinal Cortex (PRh)	**1.06**	±	**0.10**	**1.20**	±	**0.05**
anterior Reticular Thalamus (RT)	**1.26**	±	**0.08**	0.36	±	0.06
Entorhinal Cortex (EC)	0.68	±	0.08	0.57	±	0.08

*Table S14. Data shown as the Mean ± SEM variable importance to the projection (VIP) statistic. VIPs with a 95% confidence interval (CI) >1.0 are considered to be connected to the SNR ("seed") region (bold). **denotes p<0.01 and ***denotes p<0.001 significant difference from saline-treated WT mice (t-test with Bonferroni correction).*

Table S15. PLSR defined connectivity of the globus pallidus (GP) in saline-treated wild-type (WT) and Nrxn1α$^{+/-}$ mice

Genotype	Wild-type (WT)			*Neurexin1 α* $^{+/-}$		
**Treatment**	**Saline**			**Saline**		
**Region**	**Mean ± SEM**			**Mean ± SEM**		
anterior Prelimbic Cortex (aPrL)	0.81	±	0.08	0.48	±	0.07
Frontal Association Area (FRA)	0.42	±	0.06	**1.27*** **	±	**0.06**
Dorsolateral Orbital Cortex (DLO)	0.42	±	0.11	0.82	±	0.05
Ventral Orbital Cortex (VO)	1.04	±	0.11	0.85	±	0.04
Medial Orbital Cortex (MO)	0.30	±	0.06	**1.17*** **	±	**0.09**
medial Prelimbic Cortex (mPrL)	0.64	±	0.12	0.49	±	0.06
Infralimbic Cortex (IL)	**1.29**	±	**0.10**	0.47	±	0.08
Nucleus Accumbens Core (NaC)	0.69	±	0.08	**1.04**	±	**0.03**
Nucleus Accumbens Shell (NaS)	**1.51**	±	**0.07**	**1.07**	±	**0.03**
Cingualte Cortex (Cg1)	**1.29**	±	**0.10**	0.94	±	0.04
Motor Cortex (M1)	0.50	±	0.07	0.66	±	0.04
Piriform Cortex (Piri)	**1.80**	±	**0.05**	0.83	±	0.03
Insular Cortex (Ins)	0.61	±	0.08	**1.15*** **	±	**0.04**
Ventromedial Striatum (VMST)	0.35	±	0.04	**1.07*** **	±	**0.05**
Dorsolateral Striatum (DLST)	0.38	±	0.06	0.72	±	0.05
Medial Septum (MS)	0.39	±	0.15	**1.34*** **	±	**0.04**
Lateral Septum (LS)	**1.01**	±	**0.12**	**1.37**	±	**0.02**
Vertical Limb of the Diagonal Band of Broca (VDB)	0.82	±	0.13	**1.30**	±	**0.04**
Horizontal Limb of the Diagonal Band of Broca (HDB)	0.41	±	0.08	**1.19*** **	±	**0.05**
Anteromedial Thalamus (AM)	0.80	±	0.14	**1.13**	±	**0.04**
Anteroventral Thalamus (AV)	0.84	±	0.07	0.94	±	0.06
Somatosensory Cortex (SSCTX)	**1.26**	±	**0.03**	0.58	±	0.02
Mediodorsal Thalamus (MD)	0.41	±	0.05	0.48	±	0.10
Centromedial Thalamus (CM)	0.33	±	0.04	0.47	±	0.06
Centrolateral Thalamus (CL)	**1.29**	±	**0.04**	0.51	±	0.10
Ventrolateral Thalamus (VL)	0.54	±	0.12	0.90	±	0.05
Ventromedial Thalamus (VM)	0.38	±	0.09	0.77	±	0.05
Nucleus Reuniens (Re)	0.84	±	0.07	0.81	±	0.03
dorsal Reticular Thalamus (dRT)	0.40	±	0.05	0.97	±	0.08
ventral Reticular Thalamus (vRT)	0.28	±	0.05	0.81	±	0.05
Basolateral Amygdala (BLA)	0.34	±	0.07	0.85	±	0.05
Medial Amygdala (MeA)	**1.20**	±	**0.07**	1.01	±	0.05
Central Amygdala (CeA)	0.46	±	0.11	**1.39*** **	±	**0.02**
Retrosplenial Cortex (RSC)	0.63	±	0.07	0.56	±	0.06
Habenula (Hab)	0.38	±	0.13	0.95	±	0.06
Cornu Ammonis 1 (DHCA1)	0.61	±	0.09	**1.07**	±	**0.06**
Cornu Ammonis 2 (DHCA2)	0.34	±	0.07	**1.31*** **	±	**0.07**
Dentate Gyrus (DHDG)	**1.20**	±	**0.07**	0.93	±	0.04
Molecular Layer (DHML)	1.03	±	0.10	0.66	±	0.08
Auditory Cortex (AudC)	**1.71**	±	**0.02**	**1.38**	±	**0.06**
Medial Geniculate (MG)	**1.58**	±	**0.07**	**1.52**	±	**0.04**
Dorsal Subiculum (DS)	**1.74**	±	**0.04**	**1.29**	±	**0.03**
VH Cornu Ammonis 1 (VHCA1)	0.42	±	0.13	0.95	±	0.06
VH Cornu Ammonis 2 (VHCA2)	0.81	±	0.06	0.97	±	0.05
VH Cornu Ammonis 3 (VHCA3)	**1.31**	±	**0.04**	0.85	±	0.06
VH Dentate Gyrus (VHDG)	0.48	±	0.05	0.59	±	0.05
VH Molecular Layer (VHML)	**1.49**	±	**0.07**	**1.09**	±	**0.05**
Mamillary Body (MB)	0.69	±	0.07	**1.26** **	±	**0.06**
Ventral Tegmental Area (VTA)	**1.20**	±	**0.13**	**1.28**	±	**0.04**
Substantia Nigra pars Compacta (SNC)	0.92	±	0.07	0.89	±	0.06
Substantia Nigra pars Reticulata (SNR)	0.77	±	0.09	**1.50*** **	±	**0.03**
Dorsal Raphé (DR)	0.80	±	0.06	0.42	±	0.06
Median Raphé (MR)	**1.64**	±	**0.05**	0.52	±	0.05
Ventral Tegmental Nucleus (VTg)	**1.23**	±	**0.15**	0.98	±	0.04
Perirhinal Cortex (PRh)	**1.49**	±	**0.10**	**1.43**	±	**0.05**
anterior Reticular Thalamus (RT)	**1.48**	±	**0.03**	0.75	±	0.06
Entorhinal Cortex (EC)	**1.07**	±	**0.13**	0.44	±	0.06

*Table S15. Data shown as the Mean ± SEM variable importance to the projection (VIP) statistic. VIPs with a 95% confidence interval (CI) >1.0 are considered to be connected to the GP ("seed") region (bold). **denotes p<0.01 and **denotes p<0.001 significant difference from saline-treated WT mice (t-test with Bonferroni correction).*

Table S16. PLSR defined connectivity of the central amygdala (CeA) in saline-treated wild-type (WT) and *Nrxn1α*$^{+/-}$ mice

Genotype	Wild-type (WT)			*Neurexin1 α* $^{+/-}$		
**Treatment**	**Saline**			**Saline**		
**Region**	**Mean ± SEM**			**Mean ± SEM**		
anterior Prelimbic Cortex (aPrL)	0.81	±	0.10	**1.24**	±	**0.06**
Frontal Association Area (FRA)	0.47	±	0.06	**1.70*** **	±	**0.02**
Dorsolateral Orbital Cortex (DLO)	**1.69**	±	**0.07**	**1.45**	±	**0.03**
Ventral Orbital Cortex (VO)	0.49	±	0.06	1.02	±	0.06
Medial Orbital Cortex (MO)	0.77	±	0.10	0.59	±	0.04
medial Prelimbic Cortex (mPrL)	0.61	±	0.10	0.59	±	0.06
Infralimbic Cortex (IL)	0.37	±	0.05	0.44	±	0.08
Nucleus Accumbens Core (NaC)	0.84	±	0.09	0.77	±	0.04
Nucleus Accumbens Shell (NaS)	0.48	±	0.06	**1.03** **	±	**0.05**
Cingualte Cortex (Cg1)	**1.44**	±	**0.06**	0.89	±	0.03
Motor Cortex (M1)	0.45	±	0.05	**1.03* **	±	**0.02**
Piriform Cortex (Piri)	0.52	±	0.12	0.85	±	0.08
Insular Cortex (Ins)	0.42	±	0.11	0.69	±	0.03
Ventromedial Striatum (VMST)	0.98	±	0.09	0.61	±	0.06
Dorsolateral Striatum (DLST)	1.01	±	0.08	**1.14**	±	**0.05**
Medial Septum (MS)	**1.75**	±	**0.08**	**1.43**	±	**0.03**
Lateral Septum (LS)	**1.65**	±	**0.08**	**1.36**	±	**0.04**
Vertical Limb of the Diagonal Band of Broca (VDB)	**1.41**	±	**0.06**	**1.28**	±	**0.06**
Horizontal Limb of the Diagonal Band of Broca (HDB)	**1.87**	±	**0.04**	**1.60**	±	**0.02**
Anteromedial Thalamus (AM)	0.51	±	0.15	**1.50** **	±	**0.03**
Anteroventral Thalamus (AV)	0.62	±	0.11	**1.57*** **	±	**0.03**
Somatosensory Cortex (SSCTX)	0.49	±	0.07	0.46	±	0.03
Globus Pallidus (GP)	0.51	±	0.10	**1.43*** **	±	**0.03**
Mediodorsal Thalamus (MD)	0.76	±	0.09	0.89	±	0.06
Centromedial Thalamus (CM)	1.02	±	0.07	0.41	±	0.11
Centrolateral Thalamus (CL)	**1.18**	±	**0.12**	0.48	±	0.04
Ventrolateral Thalamus (VL)	**1.40**	±	**0.06**	0.65	±	0.03
Ventromedial Thalamus (VM)	**1.33**	±	**0.11**	0.50	±	0.03
Nucleus Reuniens (Re)	0.84	±	0.05	**1.09**	±	**0.06**
dorsal Reticular Thalamus (dRT)	0.72	±	0.09	**1.31* **	±	**0.06**
ventral Reticular Thalamus (vRT)	0.86	±	0.12	0.53	±	0.04
Basolateral Amygdala (BLA)	0.74	±	0.06	**1.13**	±	**0.06**
Medial Amygdala (MeA)	**1.28**	±	**0.07**	0.54	±	0.02
Retrosplenial Cortex (RSC)	0.74	±	0.07	0.79	±	0.08
Habenula (Hab)	0.74	±	0.08	0.74	±	0.06
Cornu Ammonis 1 (DHCA1)	**1.14**	±	**0.11**	0.81	±	0.06
Cornu Ammonis 2 (DHCA2)	**1.17**	±	**0.09**	**1.40**	±	**0.03**
Dentate Gyrus (DHDG)	**1.26**	±	**0.09**	0.79	±	0.08
Molecular Layer (DHML)	0.40	±	0.09	0.94	±	0.08
Auditory Cortex (AudC)	**1.38**	±	**0.09**	**1.34**	±	**0.05**
Medial Geniculate (MG)	0.69	±	0.09	1.01	±	0.07
Dorsal Subiculum (DS)	0.95	±	0.11	0.77	±	0.04
VH Cornu Ammonis 1 (VHCA1)	0.77	±	0.09	0.47	±	0.03
VH Cornu Ammonis 2 (VHCA2)	0.65	±	0.07	0.63	±	0.03
VH Cornu Ammonis 3 (VHCA3)	0.51	±	0.11	0.74	±	0.07
VH Dentate Gyrus (VHDG)	0.77	±	0.07	0.49	±	0.04
VH Molecular Layer (VHML)	**1.49**	±	**0.05**	0.85	±	0.04
Mamillary Body (MB)	0.58	±	0.10	0.65	±	0.07
Ventral Tegmental Area (VTA)	0.53	±	0.11	0.55	±	0.04
Substantia Nigra pars Compacta (SNC)	**1.16**	±	**0.04**	0.63	±	0.05
Substantia Nigra pars Reticulata (SNR)	**1.34**	±	**0.09**	**1.40**	±	**0.03**
Dorsal Raphé (DR)	0.83	±	0.11	0.50	±	0.06
Median Raphé (MR)	0.87	±	0.10	0.80	±	0.05
Ventral Tegmental Nucleus (VTg)	0.69	±	0.06	0.79	±	0.03
Perirhinal Cortex (PRh)	0.53	±	0.09	**1.29*** **	±	**0.05**
anterior Reticular Thalamus (RT)	0.39	±	0.09	**1.27*** **	±	**0.05**
Entorhinal Cortex (EC)	0.72	±	0.04	0.65	±	0.03

*Table S16. Data shown as the Mean ± SEM variable importance to the projection (VIP) statistic. VIPs with a 95% confidence interval (CI) >1.0 are considered to be connected to the CeA ("seed") region (bold). *denotes p<0.05, **denotes p<0.01 and ***denotes p<0.001 significant difference from saline-treated WT mice (t-test with Bonferroni correction).*

Table S17. PLSR defined connectivity of the dorsal hippocampus cornu ammonis 2 (DHCA2) in saline-treated wild-type (WT) and *Nrxn1α*$^{+/-}$ mice.

Genotype	Wild-type (WT)			*Neurexin1 α* $^{+/-}$		
**Treatment**	**Saline**			**Saline**		
**Region**	**Mean ± SEM**			**Mean ± SEM**		
anterior Prelimbic Cortex (aPrL)	0.66	±	0.05	0.53	±	0.05
Frontal Association Area (FRA)	**1.06**	±	**0.10**	**1.65**	±	**0.02**
Dorsolateral Orbital Cortex (DLO)	**1.05**	±	**0.07**	**1.32**	±	**0.06**
Ventral Orbital Cortex (VO)	0.92	±	0.09	**1.07**	±	**0.08**
Medial Orbital Cortex (MO)	0.68	±	0.13	0.45	±	0.06
medial Prelimbic Cortex (mPrL)	0.75	±	0.08	0.65	±	0.05
Infralimbic Cortex (IL)	0.73	±	0.09	0.65	±	0.05
Nucleus Accumbens Core (NaC)	**1.21**	±	**0.08**	0.91	±	0.05
Nucleus Accumbens Shell (NaS)	0.84	±	0.10	0.97	±	0.03
Cingualte Cortex (Cg1)	0.59	±	0.11	0.89	±	0.06
Motor Cortex (M1)	**1.11**	±	**0.08**	0.95	±	0.05
Piriform Cortex (Piri)	0.52	±	0.05	0.97	±	0.06
Insular Cortex (Ins)	0.68	±	0.09	0.89	±	0.05
Ventromedial Striatum (VMST)	**1.28**	±	**0.06**	0.83	±	0.13
Dorsolateral Striatum (DLST)	**1.59**	±	**0.05**	0.90	±	0.05
Medial Septum (MS)	**1.48**	±	**0.07**	**1.43**	±	**0.02**
Lateral Septum (LS)	**1.52**	±	**0.05**	**1.19**	±	**0.03**
Vertical Limb of the Diagonal Band of Broca (VDB)	1.03	±	0.08	**1.43**	±	**0.05**
Horizontal Limb of the Diagonal Band of Broca (HDB)	**1.52**	±	**0.08**	**1.27**	±	**0.03**
Anteromedial Thalamus (AM)	0.88	±	0.08	**1.14**	±	**0.04**
Anteroventral Thalamus (AV)	0.48	±	0.05	**1.28*** **	±	**0.06**
Somatosensory Cortex (SSCTX)	0.94	±	0.05	0.41	±	0.03
Globus Pallidus (GP)	0.48	±	0.09	**1.33*** **	±	**0.06**
Mediodorsal Thalamus (MD)	0.50	±	0.02	0.85	±	0.04
Centromedial Thalamus (CM)	0.93	±	0.05	0.64	±	0.05
Centrolateral Thalamus (CL)	0.61	±	0.02	0.57	±	0.08
Ventrolateral Thalamus (VL)	0.55	±	0.07	0.84	±	0.07
Ventromedial Thalamus (VM)	0.45	±	0.07	0.60	±	0.10
Nucleus Reuniens (Re)	0.89	±	0.06	0.80	±	0.07
dorsal Reticular Thalamus (dRT)	0.65	±	0.05	0.83	±	0.06
ventral Reticular Thalamus (vRT)	0.70	±	0.08	0.66	±	0.05
Basolateral Amygdala (BLA)	**1.12**	±	**0.10**	0.66	±	0.06
Medial Amygdala (MeA)	**1.33**	±	**0.04**	0.67	±	0.04
Central Amygdala (CeA)	**1.17**	±	**0.09**	**1.37**	±	**0.03**
Retrosplenial Cortex (RSC)	0.53	±	0.07	0.43	±	0.06
Habenula (Hab)	**1.24**	±	**0.07**	0.67	±	0.05
Cornu Ammonis 1 (DHCA1)	0.73	±	0.01	**1.04*** **	±	**0.03**
Dentate Gyrus (DHDG)	**1.15**	±	**0.04**	**1.61**	±	**0.02**
Molecular Layer (DHML)	0.53	±	0.07	**1.55*** **	±	**0.03**
Auditory Cortex (AudC)	0.81	±	0.04	**1.47*** **	±	**0.08**
Medial Geniculate (MG)	0.80	±	0.06	**1.35* **	±	**0.08**
Dorsal Subiculum (DS)	0.59	±	0.11	0.86	±	0.04
VH Cornu Ammonis 1 (VHCA1)	0.73	±	0.09	0.47	±	0.02
VH Cornu Ammonis 2 (VHCA2)	0.34	±	0.05	0.63	±	0.03
VH Cornu Ammonis 3 (VHCA3)	**1.27**	±	**0.11**	0.45	±	0.03
VH Dentate Gyrus (VHDG)	**1.45**	±	**0.08**	0.88	±	0.08
VH Molecular Layer (VHML)	**1.19**	±	**0.05**	0.92	±	0.06
Mamillary Body (MB)	0.71	±	0.11	0.81	±	0.04
Ventral Tegmental Area (VTA)	0.69	±	0.08	0.89	±	0.07
Substantia Nigra pars Compacta (SNC)	**1.60**	±	**0.07**	0.63	±	0.06
Substantia Nigra pars Reticulata (SNR)	**1.97**	±	**0.02**	**1.62**	±	**0.03**
Dorsal Raphé (DR)	0.60	±	0.07	0.71	±	0.12
Median Raphé (MR)	0.78	±	0.07	0.54	±	0.05
Ventral Tegmental Nucleus (VTg)	0.72	±	0.08	0.86	±	0.05
Perirhinal Cortex (PRh)	0.85	±	0.06	**1.43* **	±	**0.08**
anterior Reticular Thalamus (RT)	0.95	±	0.05	0.67	±	0.05
Entorhinal Cortex (EC)	0.92	±	0.05	0.49	±	0.04

*Table S17. Data shown as the Mean ± SEM variable importance to the projection (VIP) statistic. VIPs with a 95% confidence interval (CI) >1.0 are considered to be connected to the DHCA2 ("seed") region (bold). *denotes p<0.05 and ***denotes p<0.001 significant difference from saline-treated WT mice (t-test with Bonferroni correction).*

Table S18. PLSR defined connectivity of the median raphé (MR) in saline-treated wild-type (WT) and *Nrxn1α*$^{+/-}$ mice

Genotype	Wild-type (WT)			*Neurexin1 α* $^{+/-}$		
Treatment	Saline			Saline		
Region	Mean ± SEM			Mean ± SEM		
anterior Prelimbic Cortex (aPrL)	**1.48**	±	**0.06**	0.89	±	0.09
Frontal Association Area (FRA)	0.66	±	0.08	**1.14***	±	**0.05**
Dorsolateral Orbital Cortex (DLO)	0.76	±	0.10	**1.55****	±	**0.04**
Ventral Orbital Cortex (VO)	0.89	±	0.08	**1.42*****	±	**0.03**
Medial Orbital Cortex (MO)	0.30	±	0.05	**1.20****	±	**0.10**
medial Prelimbic Cortex (mPrL)	0.67	±	0.10	0.79	±	0.10
Infralimbic Cortex (IL)	0.55	±	0.06	0.82	±	0.08
Nucleus Accumbens Core (NaC)	0.54	±	0.05	**1.35*****	±	**0.05**
Nucleus Accumbens Shell (NaS)	**1.31**	±	**0.08**	0.64	±	0.06
Cingualte Cortex (Cg1)	0.66	±	0.07	0.49	±	0.06
Motor Cortex (M1)	0.74	±	0.08	0.95	±	0.10
Piriform Cortex (Piri)	**1.37**	±	**0.09**	0.48	±	0.05
Insular Cortex (Ins)	0.76	±	0.07	**1.17**	±	**0.06**
Ventromedial Striatum (VMST)	0.43	±	0.05	0.68	±	0.08
Dorsolateral Striatum (DLST)	0.78	±	0.06	**1.53*****	±	**0.04**
Medial Septum (MS)	0.36	±	0.12	0.70	±	0.07
Lateral Septum (LS)	0.78	±	0.07	0.50	±	0.07
Vertical Limb of the Diagonal Band of Broca (VDB)	0.49	±	0.11	0.55	±	0.06
Horizontal Limb of the Diagonal Band of Broca (HDB)	0.61	±	0.08	0.78	±	0.09
Anteromedial Thalamus (AM)	**1.36**	±	**0.07**	**1.28**	±	**0.05**
Anteroventral Thalamus (AV)	0.52	±	0.12	**1.34*****	±	**0.03**
Somatosensory Cortex (SSCTX)	**1.30**	±	**0.02**	**1.32**	±	**0.09**
Globus Pallidus (GP)	**1.57**	±	**0.06**	0.57	±	0.05
Mediodorsal Thalamus (MD)	**1.11**	±	**0.05**	**1.38**	±	**0.06**
Centromedial Thalamus (CM)	**1.11**	±	**0.07**	0.81	±	0.05
Centrolateral Thalamus (CL)	**1.60**	±	**0.04**	0.99	±	0.10
Ventrolateral Thalamus (VL)	0.43	±	0.15	0.82	±	0.10
Ventromedial Thalamus (VM)	0.54	±	0.11	0.80	±	0.11
Nucleus Reuniens (Re)	**1.53**	±	**0.03**	0.65	±	0.07
dorsal Reticular Thalamus (dRT)	**1.28**	±	**0.05**	0.55	±	0.04
ventral Reticular Thalamus (vRT)	0.78	±	0.08	0.93	±	0.07
Basolateral Amygdala (BLA)	0.94	±	0.07	0.71	±	0.06
Medial Amygdala (MeA)	**1.35**	±	**0.04**	**1.36**	±	**0.07**
Central Amygdala (CeA)	0.75	±	0.11	0.86	±	0.06
Retrosplenial Cortex (RSC)	0.66	±	0.09	0.96	±	0.04
Habenula (Hab)	0.35	±	0.04	0.72	±	0.08
Cornu Ammonis 1 (DHCA1)	1.02	±	0.10	0.53	±	0.05
Cornu Ammonis 2 (DHCA2)	0.64	±	0.08	0.60	±	0.08
Dentate Gyrus (DHDG)	**1.41**	±	**0.05**	0.49	±	0.05
Molecular Layer (DHML)	0.48	±	0.08	**1.18*****	±	**0.06**
Auditory Cortex (AudC)	**1.69**	±	**0.03**	0.67	±	0.06
Medial Geniculate (MG)	**1.24**	±	**0.08**	0.88	±	0.09
Dorsal Subiculum (DS)	**1.26**	±	**0.06**	1.01	±	0.06
VH Cornu Ammonis 1 (VHCA1)	0.41	±	0.08	0.70	±	0.06
VH Cornu Ammonis 2 (VHCA2)	0.55	±	0.11	**1.08**	±	**0.06**
VH Cornu Ammonis 3 (VHCA3)	0.45	±	0.08	0.92	±	0.05
VH Dentate Gyrus (VHDG)	0.82	±	0.07	**1.24**	±	**0.07**
VH Molecular Layer (VHML)	**1.53**	±	**0.02**	**1.07**	±	**0.04**
Mamillary Body (MB)	0.30	±	0.04	**1.18*****	±	**0.06**
Ventral Tegmental Area (VTA)	**1.07**	±	**0.09**	**1.37**	±	**0.04**
Substantia Nigra pars Compacta (SNC)	0.37	±	0.04	0.88	±	0.06
Substantia Nigra pars Reticulata (SNR)	0.38	±	0.06	0.55	±	0.04
Dorsal Raphé (DR)	0.61	±	0.09	0.63	±	0.08
Ventral Tegmental Nucleus (VTg)	**1.30**	±	**0.10**	**1.08**	±	**0.05**
Perirhinal Cortex (PRh)	**1.60**	±	**0.05**	0.66	±	0.05
anterior Reticular Thalamus (RT)	1.01	±	0.06	**1.34**	±	**0.05**
Entorhinal Cortex (EC)	**1.06**	±	**0.10**	**1.31**	±	**0.07**

*Table S18. Data shown as the Mean ± SEM variable importance to the projection (VIP) statistic. VIPs with a 95% confidence interval (CI) >1.0 are considered to be connected to the MR ("seed") region (bold). *denotes p<0.05, **denotes p<0.01 and ***denotes p<0.001 significant difference from saline-treated WT mice (t-test with Bonferroni correction).*
